# Paramagnetic solid-state NMR assignment and novel chemical conversion of the aldehyde group to dihydrogen *ortho* ester and hemiacetal moieties in copper(ii)- and cobalt(ii)-pyridinecarboxaldehyde complexes†

**DOI:** 10.1039/d1ra02512k

**Published:** 2021-06-09

**Authors:** Ayelén F. Crespi, Verónica M. Sánchez, Daniel Vega, Ana L. Pérez, Carlos D. Brondino, Yamila Garro Linck, Paul Hodgkinson, Enrique Rodríguez-Castellón, Juan M. Lázaro-Martínez

**Affiliations:** Universidad de Buenos Aires – CONICET, Facultad de Farmacia y Bioquímica, Instituto de Química y Metabolismo del Fármaco (IQUIMEFA) Ciudad Autónoma de Buenos Aires Argentina lazarojm@ffyb.uba.ar; Centro de Simulación Computacional para Aplicaciones Tecnológicas, CSC-CONICET Ciudad Autónoma de Buenos Aires Argentina; Universidad Nacional de General San Martín San Martín Buenos Aires Argentina; Comisión Nacional de Energía Atómica San Martín Buenos Aires Argentina; Facultad de Bioquímica y Ciencias Biológicas, Universidad Nacional del Litoral – CONICET, Ciudad Universitaria Santa Fe Argentina; FaMAF & IFEG-CONICET, Universidad Nacional de Córdoba Córdoba Argentina; Department of Chemistry, Durham University Durham UK; Facultad de Ciencias, Universidad de Málaga Málaga Spain

## Abstract

The complex chemical functionalization of aldehyde moieties in Cu(ii)- and Co(ii)-pyridinecarboxaldehyde complexes was studied. X-ray studies demonstrated that the aldehyde group (R**C**HO) of the four pyridine molecules is converted to dihydrogen *ortho* ester (R**C**(OCH_3_)(OH)_2_) and hemiacetal (R**C**H(OH)(OCH_3_)) moieties in both 4-pyridinecarboxaldehyde copper and cobalt complexes. In contrast, the aldehyde group is retained when the 3-pyridinecarboxaldehyde ligand is complexed with cobalt. In the different copper complexes, similar paramagnetic ^1^H resonance lines were obtained in the solid state; however, the connectivity with the carbon structure and the ^1^H vicinities were done with 2D ^1^H–^13^C HETCOR, ^1^H–^1^H SQ/DQ and proton spin diffusion (PSD) experiments. The strong paramagnetic effect exerted by the cobalt center prevented the observation of ^13^C NMR signals and chemical information could only be obtained from X-ray experiments. 2D PSD experiments in the solid state were useful for the proton assignments in both Cu(ii) complexes. The combination of X-ray crystallography experiments with DFT calculations together with the experimental results obtained from EPR and solid-state NMR allowed the assignment of NMR signals in pyridinecarboxaldehyde ligands coordinated with copper ions. In cases where the crystallographic information was not available, as in the case of the 3-pyridinecarboxaldehyde Cu(ii) complex, the combination of these techniques allowed not only the assignment of NMR signals but also the study of the functionalization of the substituent group.

## Abstract 2

The complex chemical functionalization of the aldehyde group was elucidated in copper and cobalt complexes for 4- and 3-pyridinecarboxaldehyde ligands.

## Graphical abstract

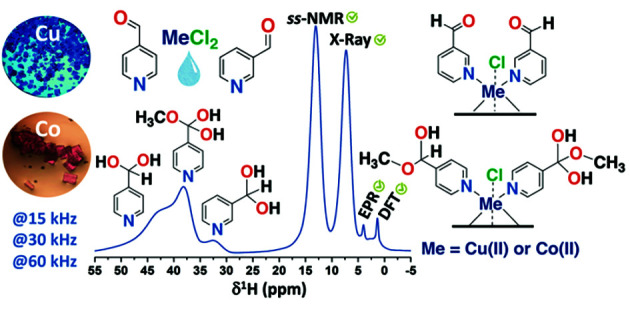

## Introduction

The search for new heterocyclic ligands to obtain novel metal complexes has become a field of great interest due to the wide applications of coordination chemistry in many areas such as medicine, hydrometallurgy and biotechnology. In particular, nitrogen heterocycles such as imidazole and pyridine molecules containing carbonyl or its hydrated groups known as *gem*-diols are widely used, due to their coordination properties,^1–4^ especially in catalysis and environmental chemistry. Working with this kind of system is a challenge since the instability of *gem*-diol^5–10^ creates uncertainty over the form of the ligand, hence careful characterization is particularly important.^9,11–14^ Taking into account the key role of NMR spectroscopy in characterizing the chemical structure of new compounds, copper and cobalt complexes containing this type of ligand are a challenge for this technique.

The study of systems containing paramagnetic centers has proved useful in a variety of fields ranging from the structure elucidation of metalloproteins^15–18^ to the characterization of catalytic organometallic complexes^9,19–21^ and Metal Organic Frameworks (MOFs).^22^ In this context, solid-state Nuclear Magnetic Resonance (ss-NMR) is a powerful tool for structural characterization of organic/inorganic compounds, providing information about connectivity and interaction between atoms in both crystalline and non-crystalline samples.^23–26^ However, the large paramagnetic shifts and short relaxation times affect the observation of the resonance signals in NMR spectra.^15,27–30^ The result of interactions in a molecule/system bearing unpaired electrons is a signal broadening and an additional nuclear shielding which scatters the lines over a wider parts per million scale in comparison with diamagnetic compounds.^31^ All these difficulties make the characterization of a paramagnetic sample by NMR techniques a challenge. In recent years, several experimental approaches have been proposed to overcome these obstacles both in static solids and under magic-angle spinning (MAS) conditions.^31–38^ The development of predictive and interpretative quantum chemical calculations is gradually changing this scenario,^30,39,40^ but there are still greater uncertainties on calculated shifts in these systems.^41^ Recently, some authors have achieved a near-complete assignment of the ss-NMR resonance signals in phenolic oximate,^30,40^ chloro-1,4,8,11-tetraazacyclotetradecane (cyclam)^27^ and 2,4-dithiohydantoin copper(ii) complexes^42^ as well as in Ni(ii)-acetylacetonate^28^ and Ru(iii) complexes^39^ through different strategies combining experimental NMR and DFT theoretical calculations. Most of these strategies refer to calculated EPR parameters, but none of them involves a real comparison with experimental EPR data, as the orientation lines, in order to provide confidence in the results obtained by quantum chemical calculations of the *g* tensor and hyperfine coupling.

In this work, a synergistic strategy combining experimental ss-NMR, single-crystal X-ray diffraction and EPR with theoretical DFT calculations was used to study the complex chemical transformation of aldehyde moieties to dihydrogen *ortho* ester and hemiacetal groups in 4-pyridinecarboxaldehyde ligands in copper and cobalt complexes. Additionally, we demonstrate how 2D ss-NMR experiments were useful to assign the spectroscopic signals, even when the interaction of copper ions with the organic ligands affected the ^1^H and ^13^C chemical shifts. For the 3-pyridinecarboxaldehyde ligand, the same studies were conducted.

## Results and discussion

### Single-crystal X-ray diffraction studies

**Fig. 1:**
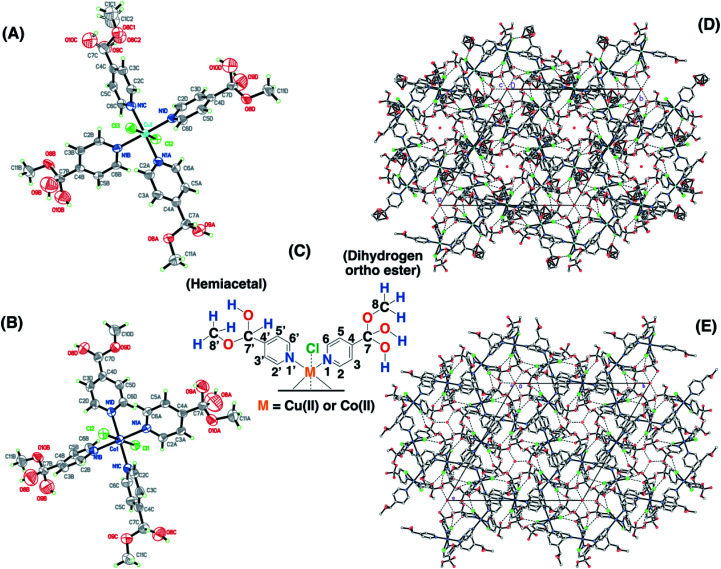
Crystal structures of 4-pyridinecarboxaldehyde copper (SC–4P–Cu–M) (A) and cobalt (SC–4P–Co–M) (B) complexes. Chemical representation of the ligands present in the X-ray structures and the numbering used throughout the manuscript (C). The displacement ellipsoids for the non-H atoms in the figure were drawn at the 50% probability level. Crystal packings for the copper (D) and cobalt complexes (E).

Single crystals for the copper and cobalt complexes were obtained from the 4-pyridinecarboxaldehyde compounds and the corresponding CuCl_2_/methanol (SC–4P–Cu–M) or CoCl_2_/methanol solutions (SC–4P–Co–M), respectively. Interestingly, in these complexes, the Cu^2+^ and Co^2+^ ions are coordinated to four N atoms (N1A, N1B, N1C and N1D) of the pyridine ligands and two Cl ones (Cl2 and Cl3) forming an octahedron (Fig. 1). The crystal packing can be described as units of Cu(C_7_H_9_NO_3_)_2_(C_7_H_9_NO_2_)_2_Cl_2_ or Co(C_7_H_9_NO_3_)_2_(C_7_H_9_NO_2_)_2_Cl_2_ stacked along the *c* axis. This stacking pattern creates channels around each of the 3-fold axes, in which electron density was observed. The electron density in each channel was modelled as oxygen atoms (O2W on 0, 0, *z* and O1W, O3W and O4 on 1/3, 2/3, *z* in the Cu(ii)-complex or O1W, O2W O3W and O4W in the Co(ii) complex) taking into account the presence of water molecules on each 3-fold axis.

Surprisingly, the aldehyde group was found to be converted into two of each dihydrogen *ortho* ester (C_7_H_9_NO_3_) and hemiacetal forms (C_7_H_9_NO_2_) at the four positions of the pyridinic ring of the ligand during crystallisation. This chemical conversion was the result of the hydration of the aldehyde group to render either *gem*-diol or hemiacetal moieties followed by the oxidation to the corresponding dihydrogen *ortho* ester moiety in either the copper or the cobalt solution.

**Fig. 2:**
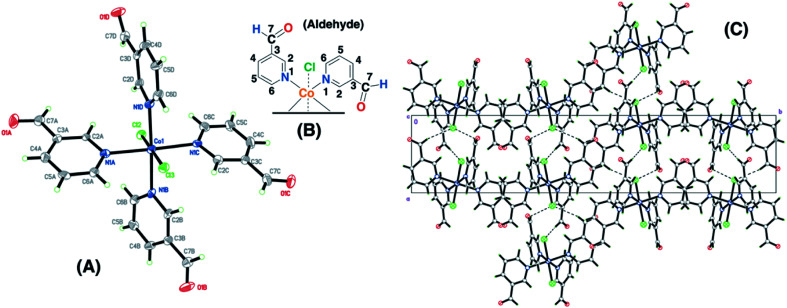
Crystal structure for the cobalt complex with 3-pyridinecarboxaldehyde (SC–3P–Co–M) (A). Chemical representation of the ligands present in the X-ray structure and the numbering used throughout the manuscript (B). The displacement ellipsoids for the non-H atoms in the figure were drawn at the 50% probability level. Crystal packing for the cobalt complex (C).

The Co(ii)–3-pyridinecarboxaldehyde complex (SC–3P–Co–M) showed the same coordination behavior for Co^2+^ as in the other 4-pyridinecarboxaldehyde Cu(ii) and Co(ii) complexes; however, in this case, the aldehyde group remained unchanged. The crystal packing can be described as chain of Co(C_6_H_5_NO)_4_Cl_2_ units running along the *a* axis. The chain is defined by two H-bond interactions with Cl2 (C6B–H6B⋯Cl2 and C6C–H6C⋯Cl2) and two interactions with Cl3 (C6A–H6A⋯Cl3 and C6D–H6D⋯Cl3) (Fig. 2). For cobalt ions, the aldehyde form crystallizes when the complex is prepared in methanol. Moreover, single-crystals for the copper complex of 3-pyridinecarboxaldehyde obtained with three different copper salts were reported and in the three cases the aldehyde form is obtained.^43–45^ For that reason, it was assumed that, under our experimental conditions, the aldehyde group was also present for the following experiments in the non-crystalline copper complex (NC–3P–Cu–M), considering the same isomorphic structure for the copper(ii) complex as in the 4-pyridinecarboxaldehyde Co/Cu complexes.

The functional groups located at the fourth position of the pyridinic ligand (hemiacetal or dihydrogen *ortho* ester groups) presented weakly interactions within the crystallographic structure in comparison to the atoms next to the metal ions (copper or cobalt) and the positional disorder was modelled by enlarged thermal ellipsoids. The disorder was evident in both single-crystals obtained for the Cu(ii) and Co(ii) complexes using the 4-pyridinecarboxaldehyde considering that the ortep plots surrounding these functional groups were enlarged. In addition, one of the terminal hemiacetal group (R–CH(OH)(OCH_3_)) in the copper complex was modeled as two disordered parts (O8C1, O9C, C1C1 and O8C2, O10C, C1C2 as part 1 and 2 respectively) refined with isotropic thermal parameters as it is shown in Fig. 1A. Additionally, sp^3^ or sp^2^ hybridization character of the terminal groups in the 4- or 3-pyridinecaboxaldehyde molecules, respectively, also contributed the higher disorder within the crystallographic structure for the hemiacetal and dihydrogen *ortho* ester groups in comparison with the aldehyde group.

### EPR studies

**Fig. 3:**
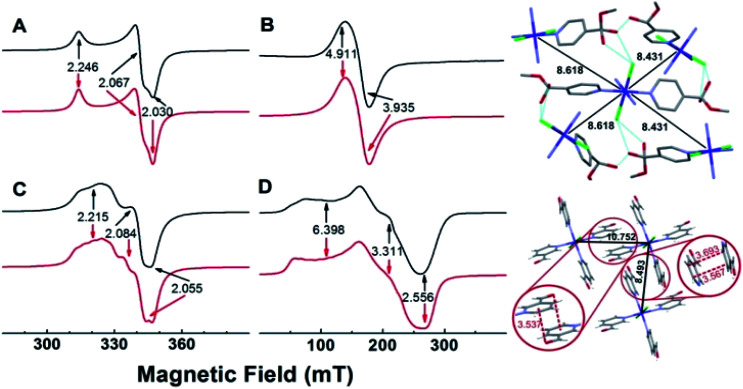
Experimental (black solid lines) and simulated EPR spectra (red solid lines) of SC–4P–Cu–M (A) and SC–4P–Co–M (B) NC–3P–Cu–M (C) and SC–3P–Co–M (D) complexes. Principal *g* values obtained by simulation are indicated with arrows. Right panel, crystal lattice view of both cobalt compounds showing the chemical pathways linking the closest metal centers (upper, SC–4P–Co–M; lower, SC–3P–Co–M); relevant distances are indicated in angstroms.

EPR spectra of powdered samples of the copper complexes obtained with either 4- or 3-pyridinecarboxaldehyde at room temperature and of the cobalt complexes obtained at 10 K, together with simulations, are shown in Fig. 3. The EPR spectrum of SC–4P–Cu–M shows nearly an axial symmetry (*g*_1,2,3_ = 2.246, 2.067, 2.030, Fig. 3A) with no evidence of hyperfine structure due to the copper nucleus (*I* = 3/2), which indicates the presence of weak, but non-negligible, isotropic exchange interactions between metal centers (*J* > *A*, where *J* and *A* are the isotropic exchange and hyperfine splitting constants, respectively).^46^

In contrast, the EPR spectrum of the NC–3P–Cu–M sample (non-crystalline Cu(ii) complex obtained from 3-pyridinecarboxaldehyde and CuCl_2_/methanol) showed the presence of a partially resolved hyperfine structure at *g*_‖_ (Fig. 3C). The simulation of this spectrum, assuming uncoupled copper centers and coincidental *g*- and *A*- tensors, showed an acceptable level of agreement with the experimental spectrum (*g*_1,2,3_ = 2.215, 2.084, 2.055, *A*_1,2,3_ = 117, non-detectable, 161 MHz). The fact that the hyperfine structure with the copper nucleus was partially resolved suggests the presence of, though very weak (*J* < *A*), non-negligible isotropic exchange interactions between copper ions.^46^ For both copper complexes, the *g*-values observed are in line with those of copper centers coordinated to four N-atoms in a square planar geometry, and indicate a ground state of a d_*x*^2^−*y*^2^_ type.

The experimental *g* values obtained for both copper compounds are well predicted by computational calculations performed on a single copper center (Tables S13 and S14†). Calculations also predicted *g*- and *A*-tensor orientations in line with those expected for copper(ii) centers in nearly square planar coordination with increased misalignment between both tensors upon metal site distortion (see Fig. S25†). The calculations for NC–3P–Cu–M yielded *A* values that were higher than the experimental ones, which is in line with the above discussed isotropic exchange phenomenon that causes the partial collapse of the hyperfine structure of the Cu(ii) ion centers.

The 4-pyridinecarboxaldehyde cobalt complex (SC–4P–Co–M) is characterized by a broad resonance at ∼160 mT (*g*_‖,⊥_ = 4.911, 3.935) and a peak-to-peak linewidth of ∼24 mT (Fig. 3B). This spectrum is in line with high-spin Co(ii) ions (*S* = 3/2) in octahedral coordination with ZFS > 0, in which the detected EPR transitions occur within the ground doublet.^47–49^ As with SC–4P–Cu–M (Fig. 3A), the lack of a hyperfine structure with the cobalt nucleus (*I* = 7/2)^50–52^ suggests the presence of inter-cobalt exchange interactions that are sufficiently strong to collapse such structure. Interestingly, the EPR spectrum of SC–3P–Co–M (Fig. 3D), as also observed in the SC–4P–Cu–M complex (Fig. 3C), showed a partially collapsed hyperfine structure with the cobalt nucleus (*I* = 7/2). This spectrum is indicative of Co(ii) ions having a high spin configuration with ZFS > 0 (*g*_1,2,3_ = 6.398, 3.311, 2.556, *A*_1,2,3_ = 1292, 421, 159 MHz).

Since isotropic exchange interactions are significantly lowered when the substituent of the pyridinic ligand in both copper and cobalt complexes is changed, we carried out a detailed comparison of the chemical pathways linking the nearest metal centers in the four compounds to determine the structural features responsible for such a change. As shown in Fig. 3 (upper right panel), the metal centers in SC–4P–Cu–M and SC–4P–Co–M complexes (Cu–Cu distances of 8.459 Å and 8.689 Å; Co–Co distances of 8.431 Å and 8.618 Å) are linked by a chemical pathway that involves the covalent interaction provided by both the dihydrogen *ortho* ester and the hemiacetal functionalization in each pyridine molecule, and a hydrogen bond mediated by the chloride ligand and the hydroxyl moiety of these groups. The same analysis performed on the SC–3P–Co–M and NC–3P–Cu–M complexes (lower right panel in Fig. 3, the nearest Co(ii) ions are situated at 8.493 Å and 10.752 Å; similar distances and chemical links are expected for the Cu(ii) ion derivative by analogy with the metal complexes with 4-pyridinecarboxaldehyde) shows that intermetal interactions are solely mediated by π–π and the –CO–π interactions in the solid structure (Fig. 3). Thus, it is concluded that the presence of the Cl–HO hydrogen bond-mediated pathway in the SC–4P–Cu–M and SC–4P–Co–M complexes, despite its considerable length and number of atoms involved, is essential to transmit exchange interactions in both complexes, irrespective of the type of metal ion or magnetic ground state.

### Solid-state NMR assignment in Cu(ii)–4-pyridinecarboxaldehyde

**Fig. 4:**
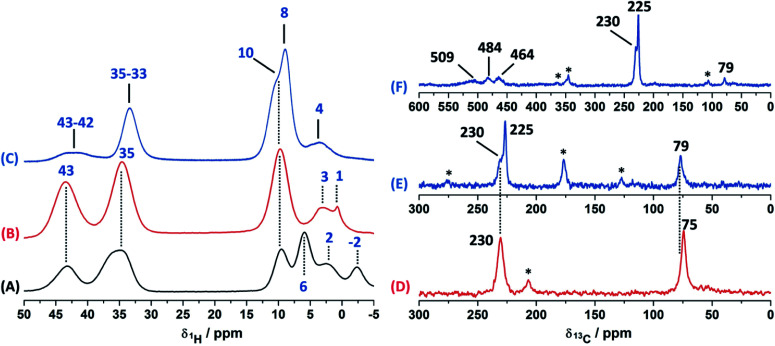
^1^H MAS ss-NMR spectra for the NC–4P–Cu–W (A), NC–4P–Cu–M (B) and SC–4P–Cu–M (C) (MAS rate: 60 kHz). ^13^C CP-MAS ss-NMR spectra for the NC–4P–Cu–M ((D) contact time = 1.5 ms and a MAS rate of 10 kHz) and SC–4P–Cu–M with different experimental conditions ((E) contact time = 2 ms and a MAS rate of 15 kHz; (F) contact time = 500 μs and a MAS rate of 18 kHz). Asterisks indicate spinning side bands.

In order to study the paramagnetic shifts on the Cu(ii)–4-pyridinecaboxaldehyde complex, 1D ss-NMR experiments were done (Fig. 4). The interactions between protons and carbon atoms with the unpaired electron of the copper center produced a strong paramagnetic shift rendering NMR spectra with a non-conventional chemical shift range, as in diamagnetic systems. Due to these interactions, the elucidation of the chemical functionalization of the organic ligands is complex and needs to be complemented and analyzed by different 2D ss-NMR experiments and X-ray results. Different synthetic procedures were done in order to achieve the assignment of some of the ^1^H-NMR signals obtained in 1D ss-NMR experiments. With this aim, three samples were prepared as indicated: NC–4P–Cu–W (non-crystalline Cu(ii) complex obtained from 4-pyridinecarboxaldehyde and CuCl_2_/water), NC–4P–Cu–M (non-crystalline Cu(ii) complex obtained from 4-pyridinecarboxaldehyde and CuCl_2_/methanol) and SC–4P–Cu–M (single crystals Cu(ii) complex obtained from 4-pyridinecarboxaldehyde and CuCl_2_/methanol).

The ^1^H MAS ss-NMR spectra for the different complexes showed that the copper centers produced a clear paramagnetic shift of the NMR signals to 34 and 43 ppm (Fig. 4). Some other NMR signals were present at a ^1^Hδ values between −2 to 10 ppm. The NC–4P–Cu–W samples shows more ^1^H resonance signals than the NC–4P–Cu–M sample at low frequency values (−2 to 6 ppm). Moreover, both NC–4P–Cu–M and NC–4P–Cu–W complexes showed the same ^13^C CP-MAS spectra, which indicates that the chemical moieties within the solids are the same. Hence the differences between the ^1^H MAS spectra of the NC–4P–Cu–W and NC–4P–Cu–M samples were ascribed to the presence of water molecules that remained in the surface or within the crystal structure in copper complexes at δ^1^H = 6, 2 and −2 ppm and δ^1^H = 3–1 ppm, respectively. These signals are reduced in number if the Cu(ii) complex is synthesized in methanol (NC–4P–Cu–M) (δ^1^H = 3–1 ppm). In this work, ^1^H resonance signals were assigned to water molecules on the basis that did not show any correlations to the carbon structure of the metal complex in the 2D ^1^H–^13^C HETCOR experiments at different contact times. Similar shift values have been previously reported for water molecules in many different systems.^7,53,54^ Particularly, Gul-E-Noor *et al.* assigned proton resonances of water molecules in the range of δ^1^H = 15–4 ppm for Cu_3_(BTC)_2_ MOF structures.^55,56^

The main difficulty in studying the chemical functionalization of the ligand in the Cu(ii) complex obtained in water is that single crystals for structural characterization could not be obtained. On the other hand, the Cu(ii) complex synthesized in methanol renders single crystals in which the ligands around the copper centers together with the chemical functionalization of the aldehyde group can unequivocally be elucidated by X-ray techniques (Fig. 1). The ^13^C CP-MAS spectrum for the SC–4P–Cu–M sample showed higher intensity resonance signals in comparison with the other Cu(ii) complexes (NC–4P–Cu–M or NC–4P–Cu–W); however, it is difficult to obtain accurate information due to the paramagnetic effect exerted by the copper centers on the pyridine ligands. The ^13^C resonances at 230 and 79 ppm were also present in the non-crystalline Cu(ii) complexes (NC–4P–Cu–M or NC–4P–Cu–W). It is noteworthy that for the correct observation of all the ^13^C resonance signals, the cross-polarization contact time needed to optimized, due to rapid relaxation associated with the copper ions. For instance, the ^13^C signals at 450–510 ppm were only observed at short contact times (100–500 μs), but the intensity of the signal at 79 ppm was highly affected under these experimental conditions. In order to enhance the intensity of the ^13^C NMR signal at 79 ppm, the contact time was set to 2 ms with the concomitant disappearance of the high frequency resonances (Fig. 4).

Considering the crystallographic information obtained for the SC–4P–Cu–M or SC–4P–Co–M and the hydration studies (ESI†) performed in methanol, it can be inferred that the *gem*-diol form is the first functional group transformation that the aldehyde group undergoes, followed by the transformation to the hemiacetal form, which is finally oxidized to the corresponded dihydrogen *ortho* ester groups during the single crystal formation (Fig. 1). For the non-crystalline Cu(ii) complexes (NC–4P–M/W), the formation of the corresponding solids (either in water or in methanol) occurred spontaneously by mixing the ligands and the copper ions in suitable proportions. In this sense, the ^1^H-MAS ss-NMR spectra for the non-crystalline Cu(ii)-complexes produced the ^1^H resonance signals at 35 and 43 ppm and were assigned to the Py–CH(OH)(OC**H**_3_) and Py–CH(O**H**)(OCH_3_) hydrogens of the hemiacetal group, respectively. When single-crystals were analyzed (SC–4P–Cu–M), the intensity of the ^1^H-NMR signal at 43 ppm was reduced with the concomitant slight shift of the signal from 35 to 33 ppm and the observation of a new resonance signal at 8 ppm. The increase of the dihydrogen *ortho* ester form during the single crystal formation allowed the assignment of the ^1^H resonance signals.

Particularly, the signal at 8 ppm was assigned to the OH group of the dihydrogen *ortho* ester moiety in the SC–4P–Cu–M sample from the evolution of the non-crystalline Cu(ii) complex (NC–4P–Cu–M) to the corresponding single-crystal Cu(ii) sample according to X-ray information and solution-state NMR studies (ESI†). Apparently, the hydrogens of the methoxy groups in both functionalizations resonated at δ^1^H = 33–35 ppm. The reduction of the signal at δ^1^H = 42–43 ppm (assigned to the hemiacetal hydrogen, RC**H**(OCH_3_)(OH)) was reduced due to the oxidation of the hemiacetal moieties to the dihydrogen *ortho* ester groups. Also, the presence of two resonances at 42 and 43 ppm may be associated to the disorder of the hemiacetal group that it was also demonstrated from the X-ray results (Fig. 1).

**Fig. 5:**
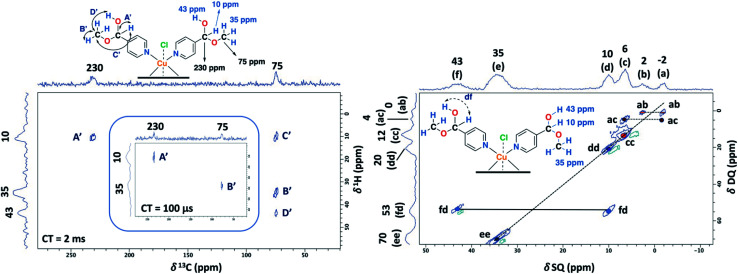
2D ^1^H–^13^C HETCOR spectrum for the NC–4P–Cu–M sample with a contact time (CT) of 2 ms. The insert shows the 2D spectrum of the same sample with a CT of 100 μs (MAS rate: 15 kHz) (left). 2D ^1^H–^1^H SQ/DQ ss-NMR spectrum for the NC–4P–Cu–W sample with two rotor periods of DQ recoupling (MAS rate: 60 kHz) (right).

To aid unambiguous assignment, 2D ^1^H–^13^C HETCOR and ^1^H–^1^H SQ/DQ NMR experiments were performed in the NC–4P–Cu–W and NC–4P–Cu–M samples (Fig. 5). The 2D spectra show clear and well resolved correlations between carbon atoms and their bonded or nearby protons getting the same results for both samples. The correlation between carbon atoms and chemically bonded protons at a contact time of 100 μs are indicated A′ and B′ (Fig. 5). At a contact time of 2 ms, additional long-range heteronuclear interactions were observed. Moreover, the 2D ^1^H–^1^H SQ/DQ ss-NMR spectrum shows the occurrence of an interaction between the hydrogen atoms of the hemiacetal and the hydroxyl groups together with the interaction among the hydrogen atoms assigned to the water molecules present in the NC–4P–Cu–W sample (Fig. 5). These correlations confirm the assignments for those resonances in the 1D ^1^H and ^13^C ss-NMR spectra associate to the hemiacetal structure in both NC–4P–Cu–M and NC–4P–Cu–W samples (Table 1).

**Table 1:** NMR assignment of the Cu(ii) complex obtained from 4-pyridinecarboxaldehyde and CuCl_2_/methanol (SC–4P–Cu–M)

Site^a^	Chemical shift ^1^H/^13^C (ppm)	Evidence for ^1^H/^13^C assignment
2–6	n.o./509, 484, 464	—
2′–6′		
7	—/225	Peak A in HETCOR exp.
		Peak D in HETCOR exp.
8	33/79	Peak E in PSD exp.
		Peaks B–D in HETCOR exp.
–OH (dihydrogen *ortho* ester)	8/—	Peak E in PSD exp.
		Peaks A and C in HETCOR exp.
7′	10/230	Peak df in SQ/DQ exp.
		Peak A′ in HETCOR exp.
		Peak F′ in PSD exp.
8′	35/75	Peaks B′ and D′ in HETCOR exp.
		Peak E′ in PSD exp.
–OH (hemiacetal)	42–43/—	Peak fd in SQ/DQ exp.
		Peak F′ in PSD exp.
		Peak D′ in HETCOR exp.

a The numbering corresponds to those in Fig. 1. n.o.: non-observed.

**Fig. 6:**
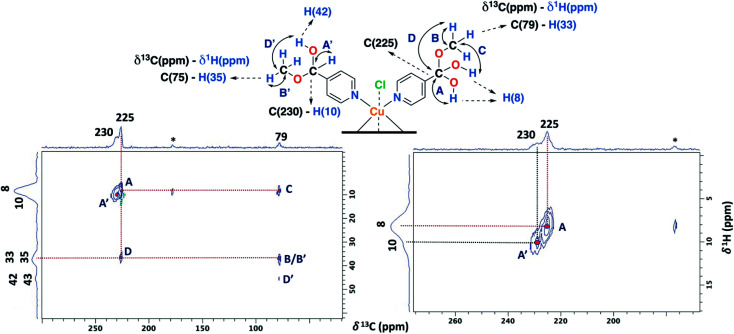
2D ^1^H–^13^C HETCOR spectrum for the SC–4P–Cu–M sample with a contact time of 1.3 ms (MAS rate: 15 kHz).

The ss-NMR analysis of the SC–4P–Cu–M sample was complex due to the presence of different dihydrogen *ortho* ester and hemiacetal pyridine ligands per copper ion (Fig. 1). However, new heteronuclear correlations were observed in the 2D HETCOR experiment for the crystalline sample in comparison with the non-crystalline complexes (Fig. 6). In particular, the carbon atom with a resonance signal at δ^13^C = 225 ppm clearly interacts with the nearby hydrogen atoms resonating at δ^1^H = 8 ppm and δ^1^H = 33 ppm using a contact time of 1.3 ms (correlations A′ and D′). This interaction was completely different from the one with δ^13^C = 230 ppm corresponding to the carbon atom of the hemiacetal moiety (correlation A′). Moreover, the signal at 79 ppm might be composed of two kinds of carbon atoms, one interacting with the hydrogen atoms at 8 and 33 ppm (correlations C and B) and the other with the hydrogen atoms at 33 and 43 ppm (B′ and D′). In this sense, the correlations observed for the hemiacetal moieties are still present together with the new signals corresponding to the dihydrogen *ortho* ester groups (Table 1). When the contact time was 50 μs the only signal observed was at 230 ppm with its bounded proton at 10 ppm. With the increasing in the contact time to 500 μs the signal at 225 ppm can be observed with a nearby proton at 8 ppm (Fig. S27†). Additionally, it is important to mention that carbons of the pyridine ring were not observed in the 2D ^1^H–^13^C HETCOR due to the inefficient of the cross-polarization process. Moreover, the low frequency regions of the ^13^C CP-MAS spectra did not present resonance signals in the ^13^C chemical shift range from −50 to −1000 ppm for the different samples. Also, the contact time and number of scans were varied in order to improve the adquisition of the spectra.

**Fig. 7:**
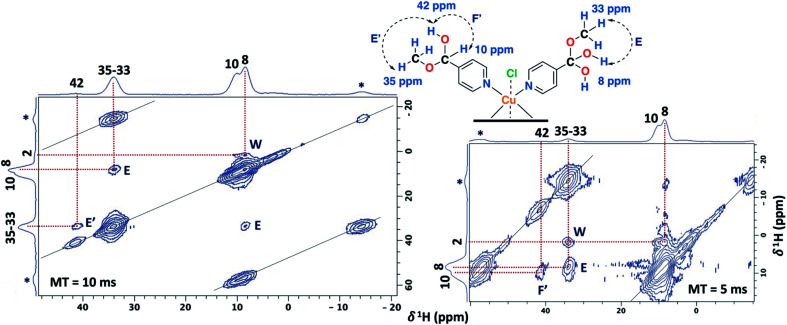
2D PSD ss-NMR spectra for the SC–4P–Cu–M sample with a mixing time (MT) of 10 (left) and 5 ms (right). (MAS rate: 32 kHz).

The 2D PSD experiments were particularly important for the correct assignment of the proton signal considering the inherent uncertainty of the DFT calculations for the proton chemical shifts (Fig. 7 and Table 1). The most informative results were obtained with mixing times of 5 and 10 ms, compared to optimal mixing times for diamagnetic compounds which are usually around 50–100 ms.^57^ This will reflect the shortening of ^1^H coherence lifetimes induced by the copper ions with a ^1^H spin-lattice relaxation time of 40 ms for the SC–4P–M sample. At a mixing time of 10 ms, the main NOE interactions were E and E′ associated to the dihydrogen *ortho* ester and hemiacetal groups, respectively. On the other hand, correlation F′ was only observed at 5 ms mixing time, allowing the assignment of the signal corresponding to the hemiacetal moieties as in the 2D ^1^H–^1^H SQ/DQ ss-NMR spectrum (Fig. 5). Surprisingly, interactions between the hydrogen atoms of water molecules and the hydrogens of the methoxy and hydroxy groups in the dihydrogen *ortho* ester ligands were also observed (correlation W).

Finally, the solid Cu(ii)- and Co(ii)-complexes obtained were dissolved in different deuterated solvents, but NMR spectra obtained were uninformative. Copper and cobalt metal ions are a strong source of relaxation, which affects the entire resonance signals of the ligands located either next or far away from the paramagnetic center when the complex is in solution (Figs. S11–S18, S23 and S24†).^58,59^

### Solid-state NMR assignment in Cu(ii)–3-pyridinecarboxaldehye

**Fig. 8:**
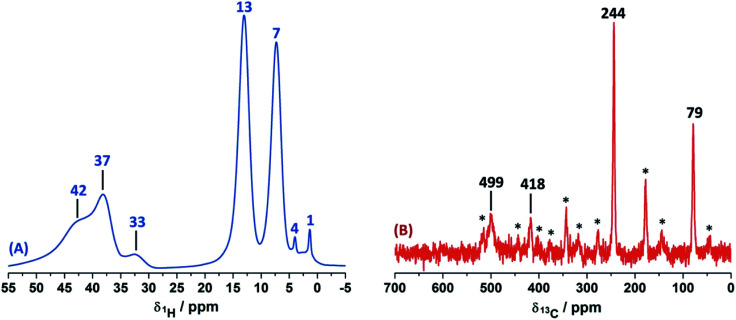
^1^H MAS (MAS rate: 32 kHz) (A) and ^13^C CP-MAS ss-NMR spectra (B) for the Cu(ii)-complex obtained from 3-pyridinecarboxaldehyde and CuCl_2_ in methanol (contact time = 500 μs; MAS rate of 15 kHz). Asterisks indicate spinning side bands.

The 1D ss-NMR results for the Cu(ii)–3-pyridinecarboxaldehyde complex are shown in Fig. 8. Once again, similar paramagnetic NMR chemical shifts are present. However, the chemical functionalization of the ligand differs in both copper complexes. For the 3-pyridinecarboxaldehyde ligand, the X-ray structure was only obtained for the cobalt(ii) complex and extrapolated the same isomorphic structure for the copper(ii) complex for the ss-NMR analysis according to EPR results and previous results.^43–45^

**Fig. 9:**
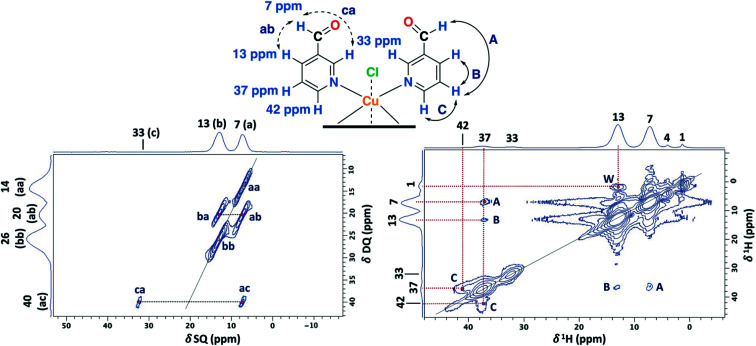
2D ^1^H–^1^H SQ/DQ with two rotor periods of DQ recoupling (left) and 2D PSD with a mixing time of 2.5 ms (right) ss-NMR spectra for the NC–3P–Cu–M sample (MAS rate: 32 kHz) (right).

As before, the ^1^H resonance signals at 1 and 4 ppm can be readily assigned to water molecules. In order to establish proximity between protons, different 2D homonuclear experiments were done (Fig. 9). In the 2D ^1^H–^1^H SQ/DQ spectrum, only the signal at 7 ppm presents different interaction with the nearby protons at 13 and 33 ppm. Taking into account this evidence, the signal at 7 ppm was ascribed to the aldehyde proton signal as a starting point for the assignment of the NMR signals. Due to the position of the carbonyl group of the aldehyde in the pyridine ring, H_7_ (δ^1^H = 7 ppm) should correlate with H_2_, H_4_ and probably with H_5_ (δ^1^H = 32, 13 and 37 ppm, respectively). H_7_ and H_4_ presented a strong autocorrelation signal at δDQ = 14 (aa) and 26 ppm (bb) respectively (Fig. 9). Besides, two correlations between the hydrogen atom of the aldehyde and aromatic protons can be observed: δDQ = 20 ppm (H_7_–H_4_, depicted as ab) and 40 ppm (H_7_–H_2_, depicted as ac). As expected, proton spin diffusion experiments (2D PSD spectrum with a mixing time of 2.5 ms) evidenced interactions between the nearby proton close to the aldehyde hydrogen (Fig. 9). For the NC–3P–Cu–M the ^1^H spin-lattice relaxation time was 10 ms. Once again, the use of 2D PSD experiments with a very short mixing time allowed in the assignment and the connectivity within the ^1^H signals. The interaction between H_4_–H_7_ cannot be well resolved in the PSD experiments due to the overlapping with the intense autocorrelation of H_4_.

**Fig. 10:**
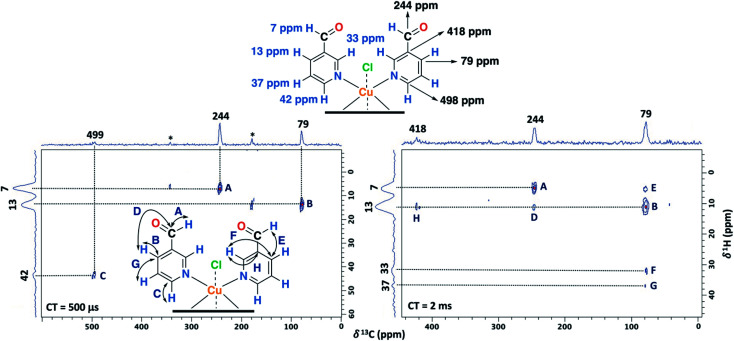
2D ^1^H–^13^C HETCOR spectra for the NC–3P–Cu–M sample with a contact time (CT) of 500 μs (left) and 2 ms (right). MAS rate: 15 kHz.

Furthermore, having identified short- and long-range interactions from the 2D ^1^H–^13^C HETCOR spectra at different contact times (Fig. 10), assignment of the observed resonances was carried out in complement to the previous results from the 2D homonuclear experiments (Table 2).

**Table 2:** NMR assignment of the Cu(ii) complex obtained from 3-pyridinecarboxaldehyde and CuCl_2_/methanol (NC–3P–Cu–M)

Site^a^	Chemical shift ^1^H/^13^C (ppm)	Evidence for ^1^H/^13^C assignment
2	33/n.o.	Peak ca in SQ/DQ exp.
3	—/418	Peak H in HETCOR exp.
4	13/79	Peak ba in SQ/DQ exp.
		Peaks B and D–G in HETCOR exp.
5	37/n.o.	Peaks A–C in PSD exp.
		Peak G in HETCOR exp.
6	42/498	Peak C in PSD exp.
		Peak C in HETCOR exp.
7	7/244	Peak ac in SQ/DQ exp.
		Peak ab in SQ/DQ exp.
		Peaks A and D in HETCOR exp.

a The numbering corresponds to those in Fig. 2 n.o.: non-observed.

Experimentally, only four peaks were observed in the ^13^C CP-MAS spectrum corresponding to six different carbon atom types of the ligand (Fig. 8). The 2D HETCOR results show that three of these signals presented heteronuclear correlations at short contact times (correlations A–C, Fig. 10). Knowing that the transfer of magnetization is limited to short distances at short contact times, the protons that correlated with these carbons at 500 μs were assumed to be directly bonded. The remaining resonance signals of the ^13^C CP MAS spectra can be readily assigned in complement the 2D SQ/DQ experiment (Fig. 8). Particularly, the carbon at 79 ppm was crucial in the assignment considering the high number of long-range interactions in the 2D HETCOR experiment at a contact time of 2 ms. It is important to mention that two carbons of the 3-pyridinecarboxaldehyde ligands were not observed in the ^13^C CP-MAS experiments.

### DFT calculations

In order to interpret the paramagnetic contributions to the total NMR chemical shifts for the Cu(ii) complexes of the 3- and 4- pyridinecarboxaldehyde (NC–3P–Cu–M and SC–4P–Cu–M samples), DFT calculations were done. Initial geometry optimizations were performed using a variety of functionals and basis set. Based on the agreement between computed and observed distances in the X-ray data, a two-step methodology optimization using UB3LYP and PBE0-D3 level of DFT was chosen for the geometry optimizations (Table S15†). For the analysis of the NC–3P–Cu–M system the information was obtained for the SC–3P–Co–M sample.

**Fig. 11:**
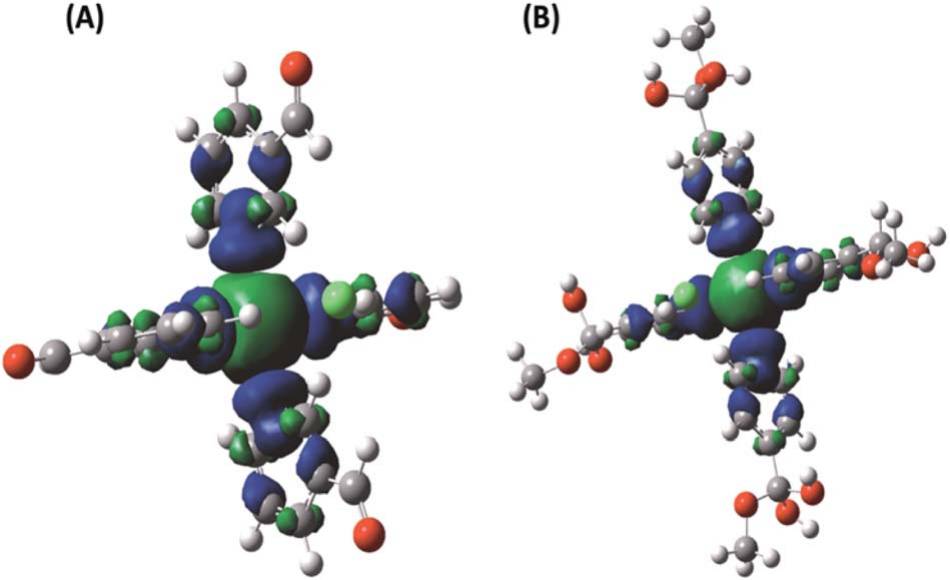
The total spin density for the Cu(ii)–3-pyridinecarboxaldehyde (A) and Cu(ii)–4-pyridinecarboxaldehyde (B) is displayed employing an isovalue for the surface density of 0.0004 a.u. Positive and negative values are shown in blue and green colors, respectively.

The paramagnetic NMR shift calculations were performed by using a well-established methodology. The spin–orbit terms (*A*_orb_) were neglected in all calculations of the A tensor of the lighter elements due to their small effect (up to approximately *δ* = 2–4 ppm for ^13^C) and the significant CPU time required for their calculation. A percentage of Hartree–Fock (HF) exchange was included in the calculations, since this is crucial to reduce the extent of spin delocalization.^30^ The total spin density (PBE0-D3 level) was computed, and the spin delocalization is illustrated in Fig. 11. In both complexes, the spin density is localized mainly in the Cu–N bonds, which involve two of the axial ligands and two of the ligands in the equatorial plane. The C atoms at the pyridine ring carry the highest spin density, in contrast to C_7_ and C_8_ (Fig. 1 and 2) in which it is vanishingly small.

^1^H-NMR resonance signals are highly sensitive to electron and spin delocalization within the paramagnetic metal–ligand complex, which leads to broadened signals at difficult-to-predict frequencies. These shifts are also difficult to predict in electronic structure calculations; calculated ^1^H chemical shifts for delocalized electronic systems, such as pyridine-metal complexes, can differ by about 100 ppm from experimental values being a typical percentage range for calculations employing a reasonable exchange–correlation functional.^31,39^ The distance between the paramagnetic Cu–Cu metal center atoms determined from the X-ray structure on the solid state is around 8–11 Å. Even though the complexes are far apart (as evidenced by the high spin delocalization degree), a ferromagnetic coupling would be present within the solid which was not considered in our calculations.^30^

The nuclei involved in the pyridine ring, C_2_–C_6_ (Fig. 1 and 2), showed the most pronounced paramagnetic shifts in both complexes, consistent with the high spin density present in these atoms (Fig. 11). As expected, the contact shift (*g*_e_*A*_FC_) is the dominant contributions to the shielding constant (Table S14†).^30,60^ In order to obtain NMR nuclear shielding constants to compare the experimental values, values corresponding to chemically equivalent atoms had to be averaged. Fig. S26† shows the variation of shifts between sites for ^13^C nuclei, which were found to be dependent on dihedral angles. Thus, the size and complexity of these systems made their optimization challenging. However, as it can be seen, there is practically no dispersion of the data; in fact, differences were observed only in those cases where the dihedral angle varied in one degree.

The ^13^C NMR calculations support the NMR assignment of NC–3P–Cu–M and SC–4P–Cu–M samples; Tables 3 and 4 show reasonable agreement between experimental and computational results considering the high degree of spin delocalization present in these compounds (Fig. 11). In both systems, there are resonances that are not observed experimentally: δ^13^C = −139, −140, −142 and −144 ppm in SC–4P–Cu–M sample, and δ^13^C = 150 and 553 ppm in NC–3P–Cu–M sample. This difference could be ascribed to the proximity of these nuclei to the paramagnetic center, resulting in very short *T*_1_ and *T*_2_ relaxation times. The complete assignments are shown in Tables 3 and 4.

**Table 3:** Calculated and experimental ^13^C chemical shifts for SC–4P–Cu–M sample (expressed in ppm relative to TMS) and the difference between experimental and calculated shifts (*Δ*). The distance to the copper center is shown for each carbon. As the substituent groups of the heterocyclic ring are different in the two complexes, they are presented separately. The numbering corresponds to that in Fig. 1 a

Signal	4-Dihydrogen *ortho* ester moiety δ^13^C NMR (ppm)		4-Hemiacetal moiety δ^13^C NMR (ppm)	
	Exp./calc., *Δ*	Distance to Cu(ii) (Å)	Exp./calc., *Δ*	Distance to Cu(ii) (Å)
C_2_	482/397, 85	2.9	464/293, 171	2.9
C_3_	n.o./−144	4.3	n.o./−142	4.3
C_4_	510/314, 196	4.8	511/315, 196	4.8
C_5_	n.o./−139	4.3	n.o./−140	4.3
C_6_	482/307, 175	2.9	464/304, 160	2.9
C_7_	225/236, −11	6.4	—	—
C_7′_	—	—	231/250, −19	6.4
C_8_/C_8′_	80/43, 37	8.3	80/50, 30	8.3

a n.o.: non-observed.

**Table 4:** Calculated and experimental ^13^C chemical shifts for NC–3P–Cu–M (expressed in ppm relative to TMS) and difference between experimental and calculated shifts (*Δ*). The distance to the copper center is shown for each carbon. The numbering corresponds to that in Fig. 2 a

Carbon signal	Cu(ii)–3-Pyridinecarboxaldehyde δ^13^C NMR (ppm)	
	Exp./calc., *Δ*	Distance to Cu(ii) (Å)
C_2_	n.o./150	2.9
C_3_	418/518, −100	4.3
C_4_	79/37, 42	4.8
C_5_	n.o./553	4.3
C_6_	498/353, 145	2.9
C_7_	244/225, 19	5.4

a n.o.: non-observed.

## Experimental

### Materials

3-Pyridinecarboxyaldehyde (98%), 4-pyridinecarboxyaldehyde (97%), copper chloride (CuCl_2_·2H_2_O, 99.95%), cobalt chloride (CoCl_2_·6H_2_O, 98%), deuterium oxide (D_2_O, 99.9 atom %D), dimethylsulfoxide-d_6_ (DMSO-d_6_, 99.96 atom %D) and methanol-d_4_ (CD_3_OD, >99.8 atom %D) were purchased from Sigma-Aldrich and used without further purification.

### General procedure for the synthesis of copper and cobalt complexes with pyridinecarboxaldehyde

Copper chloride or cobalt chloride (0.194 mmol) were dissolved in 0.8 mL of methanol and added with stirring to saturation to achieve 4.036 mmol of 3- or 4-pyridinecarboxaldehyde (initial mixture).

For single-crystal materials, 15 mL of methanol were also added to the initial mixture. Reaction mixtures turned either dark blue or dark pink upon formation of the copper or cobalt complexes, respectively. Metal complex solutions were stored at 2 °C until single crystals appeared. After that, crystals were filtered, washed with methanol and vacuum-dried. The reaction yields for Cu(ii)–4-pyridinecarboxaldehyde obtained with CuCl_2_ (SC–4P–Cu–M), Co(ii)–4-pyridinecarboxaldehyde obtained with CoCl_2_ (SC–4P–Co–M) and Co(ii)- with 3-pyridinecarboxaldehyde with CoCl_2_ (SC–3P–Co–M) were 15%, 15% and 16%, respectively.

For non-crystalline materials, 3 mL of methanol were also added to the initial mixture. Reaction mixtures turned dark blue upon formation of the complex. Metal complex solutions were stored at room temperature until the solid appeared after 30 min. The solid was then filtered, washed with methanol and vacuum-dried. The reaction yields for Cu(ii)–4-pyridinecarboxaldehyde obtained with CuCl_2_ (NC–4P–Cu–M) and Cu(ii)–3-pyridinecarboxaldehyde obtained with CuCl_2_ (NC–3P–Cu–M) were 15% and 20%, respectively. Particularly, the copper complex for the 4-pyridinecarboxaldehyde can be synthetized replacing methanol for water (NC–4P–Cu–W) (yield: 25%).

### Single-crystal X-ray crystallography studies

Single-crystal X-ray diffraction data were collected at 100 or 293 K, using a Bruker D8 Quest ECO diffractometer (Bruker AXS). The strategy employed for the collection and reduction of data followed standard the procedures implemented in the APEX3 Control Software (Bruker AXS). Different reflections were collected for each sample (specified in the ESI†).

### EPR measurements

X-Band CW-EPR measurements were performed in a Bruker EMX-Plus spectrometer equipped with an Oxford helium continuous-flow cryostat and a rectangular cavity at a field modulation frequency of 100 kHz. EPR spectra were simulated with the EasySpin toolbox based on MATLAB®.^61^ Samples for EPR spectroscopy were prepared by grinding single crystals of the compounds and placed inside 4 mm inner diameter EPR quartz tubes. EPR spectra of the copper(ii) were obtained at room temperature while spectra for the cobalt(ii) compounds were obtained at 10 K to slow down electronic relaxation.

### Solid-state NMR studies

Solid-state Nuclear Magnetic Resonance (ss-NMR) data were acquired with a Bruker Avance-III HD spectrometer equipped with a 14.1 T narrow bore magnet operating at Larmor frequencies of 600.09 MHz and 150.91 MHz for ^1^H and ^13^C, respectively. Powdered samples were packed into 3.2 and 2.5 mm ZrO_2_ rotors and rotated at room temperature at magic angle spinning (MAS) rates of 15 or 32 kHz, respectively. Particularly, the ^1^H experiments performed at a MAS rate of 60 kHz were done at Larmor frequencies of 499.7 MHz for ^1^H using a 1.3 mm ZrO_2_ rotor. ^13^C CP-MAS (cross-polarization and magic angle spinning) experiments were done in a 3.2 mm MAS probe. Different contact times during CP were used (0.1–2.0 ms) in order to obtain the highest signal-to-noise ratio in each scan and in the total time of the experiments, with number of scans between 2000 and 10 000. The SPINAL64 sequence (small phase incremental alternation with 64 steps) was used for heteronuclear decoupling during acquisition.^62^ The 2D ^1^H–^13^C HETCOR experiment in the solid state was recorded following the sequence presented by van Rossum *et al.*^63^ in a 3.2 MAS probe. The contact times for the CP were varied from 100 μs to 2 ms in order to sense near or far away interactions between ^13^C and ^1^H, respectively. ^1^H-MAS and 2D homonuclear correlation experiments (2D PSD and 2D SQ/DQ) were recorded in either a 2.5 or a 1.3 mm MAS probe as indicated for each sample. The 2D SQ/DQ MAS spectra were recorded using the back-to-back (BaBa) pulse sequence with excitation and reconversion times of two rotor period.^64^ Chemical shifts for ^13^C and ^1^H (expressed in ppm) are relative to adamantane (δ^13^C = 38.5 ppm) and (CH_3_)_4_Si (δ^1^H = 0 ppm), respectively.

### Quantum chemical calculations

#### Geometry optimization

All DFT calculations were performed with the Gaussian 09 package.^65^ In order to obtain a precise geometry and electronic structure for the NMR calculations, the geometry optimizations were performed under a two-step scheme. Firstly, starting from the X-ray complex structure and leaving the N–Cu bonds fixed, a geometry optimization was carried out using the unrestricted B3LYP (Becke three- parameter Lee–Yang–Parr)^66,67^ exchange correlation functional with the LANL2DZ basis set for Cu and 6-31++G** for C, H, O, N and Cl.^68,69^ This setting led to an optimized complex within a non-computationally demanding scheme that maintained the experimental X-ray structure.

The second geometry optimization was performed departing from the obtained complex following the more accurate DFT methodology implemented by Bühl *et al.*^30^ and Dawson *et al.*:^40^ the PBE0 hybrid functional^70–72^ with a D3 van der Waals correction was employed for all atoms, and an augmented Wachters basis set^73,74^ for Cu(ii) (8s7p4d) and the 6-31++G** basis set for C, H, O, N and Cl were used.

Particularly, for the Cu(ii)–3-pyridinecarboxaldehyde metal complex, the X-ray structure could not be determined experimentally. Therefore, the starting structure for this complex was created with the results obtained by ESR, in which copper is coordinated by four pyridine rings and two chlorine atoms; and the metal–ligand bond distances are those determined from the Co(ii)–3-pyridinecarboxaldehyde X-ray structure. This strategy was proposed considering that both M(II)–3-pyridinecarboxaldehyde (M = Cu or Co) and M(II)–4-pyridinecarboxaldehyde (M = Cu or Co) have the same square planar coordination.

#### NMR chemical shifts and EPR parameters

NMR chemical shifts were calculated with Gaussian 09 and EPR parameters (*g*- and *A*-tensors) and analyzed with the ORCA 4.0.1 software (SCF convergence and fine integration Grid, Grid5 option).^75,76^ The *σ*_orb_, *g* and *A* tensors were computed at the PBE0-D3 level using a 9s7p4d basis set on Cu(ii),^30,77^ and the IGLO-II basis on the ligands.^30,40,78–84^ The *σ*_orb_ calculations employed gauge-including atomic orbitals and fine integration grids as implemented in Gaussian 09. All NMR and EPR properties were computed using the same functional/basis-set combinations.

The chemical shifts (*δ*) were reported relative to the reference compound, typically tetramethylsilane (TMS), for ^1^H and ^13^C according to:

1 *δ* = *σ*_iso(orb)_(TMS) − *σ*_iso(orb)_

where the isotropic orbital shielding of the reference compound is computed using the same methodology.

Magnetic shielding tensors *σ* were computed as follows:^29,39,40,81,85^2
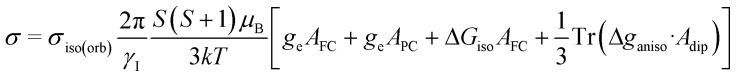
where *σ*_iso(orb)_ is the isotropic orbital shielding, *S* is the effective spin, *μ*_B_ is the Bohr magneton, *kT* is the thermal energy, *g*_e_ is the free-electron and *γ*_I_ is the gyromagnetic ratio of the nucleus. *A*_FC_ and *A*_PC_ are the isotropic Fermi-contact and pseudo-contact term arising from spin–orbit corrections to the *A*-tensor, *A*_dip_ is the anisotropic traceless spin-dipolar contribution and Δ*g*_iso_ is the isotropic part of the *g*-tensor.

## Conclusions

In this work, we studied in detail the chemical functionalization of copper complexes derived from 4- and 3-pyridinecarboxaldehyde by means of different spectroscopic techniques and theoretical calculations. Also, the cobalt complexes were studied.

Two different copper samples were analyzed for the 4-pyridinecarboxaldehyde ligand. For the non-crystalline copper complex (NC–4P–Cu–M), the hemiacetal group was the first chemical transformation of the aldehyde group present in the solid sample according to hydration NMR studies for the free-ligand in the solution state in deuterated methanol. Then, single crystal sample was obtained for the copper complex (SC–4P–Cu–M), which allowed to study the chemical transformation from the non-crystalline to the crystalline state by the combination of X-ray crystallography and solid-state NMR techniques. Crystal structures of 4-pyridinecarboxaldehyde copper (SC–4P–Cu–M) and cobalt complexes (SC–4P–Co–M) showed interesting results concerning the hemiacetal and dihydrogen *ortho* ester moieties observed at the fourth position of the pyridinic rings. Moreover, the ^1^H-MAS ss-NMR spectra at 60 kHz show that the chemical evolution from the non-crystalline to the single-crystal state give rise to a new signal at 8 ppm and the concomitant reduction of the signal at 42 ppm ascribed to the oxidation of the hemiacetal group to the dihydrogen *ortho* ester group.

For the 3-pyridinecarboxaldehyde ligand only single crystals for the cobalt complex were obtained and the aldehyde moiety was present at the third position of the pyridinic ring. In this sense, EPR was particularly important in order to consider the geometry and the same isomorphic structure for the copper(ii) system in the 3-pyridinecarboxaldehyde as in the 4-pyridinecarboxaldehyde Co/Cu complexes.

In the different copper complexes (NC–4P–Cu–M, SC–4P–Cu–M and NC–3P–Cu–M), similar paramagnetic ^1^H resonance lines were obtained; however, the connectivity with the carbon structure and the ^1^H vicinities were done with 2D ^1^H–^13^C HETCOR, ^1^H–^1^H SQ/DQ and PSD experiments. Particularly, the ^1^H spin diffusion experiments in the solid state were especially useful for the proton assignments in the Cu(ii) complexes using very short mixing times (1–10 ms) according with the ^1^H spin-lattice relaxation times for the samples (10–40 ms).

The paramagnetic effects exerted by the copper ions were completely different in the pyridinic systems allowing a more detailed assignment for the aromatic ring in the 3-pyridinecarboxaldehyde than in the 4-pyridinecarboxaldehyde due to the inefficient of the cross-polarization process used for the acquisition of the ^13^C CP-MAS and 2D ^1^H–^13^C HETCOR experiments.

DFT calculations helped to predict paramagnetic shifts in fairly good agreement and provide a basis for the interpretation of the position of the signals in the spectrum. Theoretical calculations of EPR parameters (Cu *g*-tensors) were in excellent agreement with the experimental data. For the copper complexes in this study, it was confirmed that the orientation of each of the ligands had a significant influence on the magnetic shielding tensors. Despite being a bulky and complicated system, the chemical shielding values for ^13^C nuclei were obtained. In this kind of systems an accurate modelling for solids with a complex structure and bearing bulky molecules is still a challenge. Further works are needed to improve the calculation methodology for the metal complexes described and to establish a better relationship between spin distributions, magnetic shielding tensors and crystal structure.

## Conflicts of interest

There are no conflicts to declare.

## Supplementary Material

RA-011-D1RA02512K-s001

RA-011-D1RA02512K-s002

## References

[cit1] Barszcz B. (2005). Coordination properties of didentate N,O heterocyclic alcohols and aldehydes towards Cu(II), Co(II), Zn(II) and Cd(II) ions in the solid state and aqueous solution. Coord. Chem. Rev..

[cit2] Casas J. S., Castiñeiras A., Rodríguez-Argüelles M. C., Sánchez A., Sordo J., Vázquez-López A., Vázquez-López E. M. (2000). Diorganotin(IV) complexes of imidazole-2-carbaldehyde thiosemicarbazone (H2ImTSC). The crystal and molecular structures of the “free” ligand and of [SnMe2(ImTSC)]. J. Chem. Soc., Dalton Trans..

[cit3] Woo H. Y., So H., Pope M. T. (1996). Trimetallo derivatives of lacunary 9-tungstosilicate heteropolyanions. 2. Isotropic NMR shifts in pyridine-type ligands coordinated to the paramagnetic 9-tungsto-3-cuprio(II)silicate anion. J. Am. Chem. Soc..

[cit4] Wang L., Song B., Khalife S., Li Y., Ming L. J., Bai S., Xu Y., Yu H., Wang M., Wang H., Li X. (2020). Introducing Seven Transition Metal Ions into Terpyridine-Based Supramolecules: Self-Assembly and Dynamic Ligand Exchange Study. J. Am. Chem. Soc..

[cit5] Lázaro Martínez J. M., Romasanta P. N., Chattah A. K., Buldain G. Y. (2010). NMR Characterization of Hydrate and Aldehyde Forms of Imidazole-2-carboxaldehyde and Derivatives. J. Org. Chem..

[cit6] Crespi A. F., Vega D., Chattah A. K., Monti G. A., Buldain G. Y., Lázaro-Martínez J. M. (2016). gem-Diol and Hemiacetal Forms in Formylpyridine and Vitamin-B6-Related Compounds: Solid-State NMR and Single-Crystal X-ray Diffraction Studies. J. Phys. Chem. A.

[cit7] Crespi A. F., Byrne A. J., Vega D., Chattah A. K., Monti G. A., Lázaro-Martínez J. M. (2018). Generation and Stability of the gem-Diol Forms in Imidazole Derivatives Containing Carbonyl Groups. Solid-State NMR and Single-Crystal X-ray Diffraction Studies. J. Phys. Chem. A.

[cit8] Crespi A. F. , Campodall'Orto V. and Lázaro-Martínez J. M., in Diols Synthesis and Reactions, ed. E. Ballard, Nova Science Publishers, Inc., New York, 2020, pp. 1–38

[cit9] Tasiopoulos A. J., Perlepes S. P. (2008). Diol-type ligands as central ‘players’ in the chemistry of high-spin molecules and single-molecule magnets. Dalton Trans..

[cit10] Giannopoulos D. P., Cunha-Silva L., Ballesteros-Garrido R., Ballesteros R., Abarca B., Escuer A., Stamatatos T. C. (2016). New structural motifs in Mn cluster chemistry from the ketone/gem-diol and bis(gem-diol) forms of 2,6-di-(2-pyridylcarbonyl)pyridine: {MnII4MnIII2} and {MnII4MnIII6} complexes. RSC Adv..

[cit11] Bravo-García L., Barandika G., Bazán B., Urtiaga M. K., Arriortua M. I. (2015). Thermal stability of ionic nets with CuII ions coordinated to di-2-pyridyl ketone: Reversible crystal-to-crystal phase transformation. Polyhedron.

[cit12] Efthymiou C. G., Raptopoulou C. P., Psycharis V., Tasiopoulos A. J., Escuer A., Perlepes S. P., Papatriantafyllopoulou C. (2013). Copper(II)/di-2-pyridyl ketone chemistry: A triangular cluster displaying antisymmetric exchange *versus* an 1D coordination polymer. Polyhedron.

[cit13] Liu J. L., Lin W. Q., Chen Y. C., Gõmez-Coca S., Aravena D., Ruiz E., Leng J. D., Tong M. L. (2013). CuII-GdIII cryogenic magnetic refrigerants and Cu8Dy9 single-molecule magnet generated by *in situ* reactions of picolinaldehyde and acetylpyridine: Experimental and theoretical study. Chem.–Eur. J..

[cit14] Wang H.-S., Yang F.-J., Long Q.-Q., Huang Z.-Y., Chen W., Pan Z.-Q. (2017). Syntheses, crystal structures, and magnetic properties of a family of heterometallic octanuclear [Cu6Ln2] (Ln = Dy(III), Tb(III), Ho(III), Er(III), and Gd(III)) complexes. New J. Chem..

[cit15] Bertarello A., Benda L., Sanders K. J., Pell A. J., Knight M. J., Pelmenschikov V., Gonnelli L., Felli I. C., Kaupp M., Emsley L., Pierattelli R., Pintacuda G. (2020). Picometer Resolution Structure of the Coordination Sphere in the Metal-Binding Site in a Metalloprotein by NMR. J. Am. Chem. Soc..

[cit16] Bertini I., Emsley L., Lelli M., Luchinat C., Mao J., Pintacuda G. (2010). Ultrafast MAS solid-state NMR permits extensive 13C and 1H detection in paramagnetic metalloproteins. J. Am. Chem. Soc..

[cit17] Bertini I., Luchinat C., Parigi G., Pierattelli R. (2008). Perspectives in paramagnetic NMR of metalloproteins. Dalton Trans..

[cit18] Cerofolini L., Staderini T., Giuntini S., Ravera E., Fragai M., Parigi G., Pierattelli R., Luchinat C. (2018). Long-range paramagnetic NMR data can provide a closer look on metal coordination in metalloproteins. J. Biol. Inorg Chem..

[cit19] Savva M., Skordi K., Fournet A. D., Thuijs A. E., Christou G., Perlepes S. P., Papatriantafyllopoulou C., Tasiopoulos A. J. (2017). Heterometallic MnIII4Ln2 (Ln = Dy, Gd, Tb) Cross-Shaped Clusters and Their Homometallic MnIII4MnII2 Analogues. Inorg. Chem..

[cit20] Efthymiou C. G., Raptopoulou C. P., Psycharis V., Tasiopoulos A. J., Escuer A., Perlepes S. P., Papatriantafyllopoulou C. (2013). Copper(II)/di-2-pyridyl ketone chemistry: A triangular cluster displaying antisymmetric exchange *versus* an 1D coordination polymer. Polyhedron.

[cit21] Baute D., Arieli D., Neese F., Zimmermann H., Weckhuysen B. M., Goldfarb D. (2004). Carboxylate binding in copper histidine complexes in solution and in zeolite Y: X- and W-band pulsed EPR/ENDOR combined with DFT calculations. J. Am. Chem. Soc..

[cit22] Lehr M., Paschelke T., Trumpf E., Vogt A. M., Näther C., Sönnichsen F. D., McConnell A. J. (2020). A Paramagnetic NMR Spectroscopy Toolbox for the Characterisation of Paramagnetic/Spin-Crossover Coordination Complexes and Metal–Organic Cages. Angew. Chem., Int. Ed..

[cit23] Uldry A.-C., Griffin J. M., Yates J. R., Pérez-Torralba M., María M. D. S., Webber A. L., Beaumont M. L. L., Samoson A., Claramunt R. M., Pickard C. J., Brown S. P. (2008). Quantifying weak hydrogen bonding in uracil and 4-cyano-4’-ethynylbiphenyl: a combined computational and experimental investigation of NMR chemical shifts in the solid state. J. Am. Chem. Soc..

[cit24] V Dudenko D., Williams P. A., Hughes C. E., Antzutkin O. N., Velaga S. P., Brown S. P., Harris K. D. M. (2013). Exploiting the Synergy of Powder X- ray Diffraction and Solid-State NMR Spectroscopy in Structure Determination of Organic Molecular Solids. J. Phys. Chem. C.

[cit25] Higashi K., Yamamoto K., Pandey M. K., Mroue K. H., Moribe K., Yamamoto K., Ramamoorthy A. (2014). Insights into atomic-level interaction between mefenamic acid and Eudragit EPO in a supersaturated solution by high-resolution magic-angle spinning NMR spectroscopy. Mol. Pharm..

[cit26] Mafra L., Santos S. M., Siegel R., Alves I., Almeida Paz F. A., Dudenko D., Spiess H. W. (2012). Packing Interactions in Hydrated and Anhydrous Forms of the Antibiotic Ciprofloxacin: a Solid-State NMR, X-ray Diffraction, and Computer Simulation Study. J. Am. Chem. Soc..

[cit27] Kumara Swamy S. K., Karczmarska A., Makowska-Janusik M., Kassiba A., Dittmer J. (2013). Solid-state NMR correlation experiments
and distance measurements in paramagnetic metalorganics exemplified by Cu-cyclam. ChemPhysChem.

[cit28] Rouf S. A., Jakobsen V. B., Mareš J., Jensen N. D., McKenzie C. J., Vaara J., Nielsen U. G. (2017). Assignment of solid-state 13C and 1H NMR spectra of paramagnetic Ni(II) acetylacetonate complexes aided by first-principles computations. Solid State Nucl. Magn. Reson..

[cit29] Ke Z., Jamieson L. E., Dawson D. M., Ashbrook S. E., Bühl M. (2019). NMR chemical shifts of urea loaded copper benzoate. A joint solid-state NMR and DFT study. Solid State Nucl. Magn. Reson..

[cit30] Bühl M., Ashbrook S. E., Dawson D. M., Doyle R. A., Hrobárik P., Kaupp M., Smellie I. A. (2016). Paramagnetic NMR of Phenolic Oxime Copper Complexes: A Joint Experimental and Density Functional Study. Chem.–Eur. J..

[cit31] Bertini I. , Luchinat C., Parigi G. and Ravera E., in NMR of Paramagnetic Molecules, ed. I. Bertini, C. Luchinat, G. Parigi and E. B. T. Ravera, Elsevier, Boston, 2017, pp. 127–150

[cit32] Hodgkinson P. (2020). NMR crystallography of molecular organics. Prog. Nucl. Magn. Reson. Spectrosc..

[cit33] Magusin P. C. M. M. , Seymour I. D., Pecher O. and Grey C. P., in Modern Methods in Solid-state NMR: A Practitioner's Guide, ed. P. Hodgkinson, The Royal Society of Chemistry, 2018, pp. 322–355

[cit34] Kervern G., Pintacuda G., Zhang Y., Oldfield E., Roukoss C., Kuntz E., Herdtweck E., Basset J. M., Cadars S., Lesage A., Copéret C., Emsley L. (2006). Solid-state NMR of a paramagnetic DIAD-FeII catalyst: Sensitivity, resolution enhancement, and structure-based assignments. J. Am. Chem. Soc..

[cit35] Shaibat M. A., Casabianca L. B., Wickramasinghe N. P., Guggenheim S., De Dios A. C., Ishii Y. (2007). Characterization of polymorphs and solid-state reactions for paramagnetic systems by 13C solid-state NMR and *ab initio* calculations. J. Am. Chem. Soc..

[cit36] Ishii Y., Wickramasinghe N. P., Chimon S. (2003). A new approach in 1D and 2D 13C high-resolution solid-state NMR spectroscopy of paramagnetic organometallic complexes by very fast magic-angle spinning. J. Am. Chem. Soc..

[cit37] Wickramasinghe N. P., Shaibat M., Ishii Y. (2005). Enhanced sensitivity and resolution in 1H solid-state NMR spectroscopy of paramagnetic complexes under very fast magic angle spinning. J. Am. Chem. Soc..

[cit38] Pell A. J., Pintacuda G., Grey C. P. (2019). Paramagnetic NMR in solution and the solid state. Prog. Nucl. Magn. Reson. Spectrosc..

[cit39] Rastrelli F., Bagno A. (2010). Predicting the 1H and 13C NMR spectra of paramagnetic Ru(III) complexes by DFT. Magn. Reson. Chem..

[cit40] Dawson D. M., Ke Z., Mack F. M., Doyle R. A., Bignami G. P. M., Smellie I. A., Bühl M., Ashbrook S. E. (2017). Calculation and experimental measurement of paramagnetic NMR parameters of phenolic oximate Cu(II) complexes. Chem. Commun..

[cit41] Bühl M., van Mourik T. (2011). NMR spectroscopy: Quantum-chemical calculations. Wiley Interdiscip. Rev.: Comput. Mol. Sci..

[cit42] Ahmedova A., Marinova P., Paradowska K., Marinov M., Wawer I., Mitewa M. (2010). Structure of 2,4-dithiohydantoin complexes with copper and nickel: Solid-state NMR as verification method. Polyhedron.

[cit43] Cruz-Enriquez A., Baez-Castro A., Höpfl H., Parra-Hake M., Campos-Gaxiola J. J. (2012). Tetrakis(μ-acetato-κ2 O:O’)-bis[(3-
pyridinecarboxaldehyde-κN’)]dicopper(II)(Cu-Cu). Acta Crystallogr., Sect. E: Struct. Rep. Online.

[cit44] Himoto K., Horii T., Syoji T., Okubo T., Maekawa M., Kuroda-Sowa T. (2018). A new semiconducting coordination polymer consisting of copper(I)-iodide and 3-pyridinecarboxaldehyde. Inorg. Chem. Commun..

[cit45] Saravanabharathi D., Nethaji M., Samuelson A. G. (2002). Is copper(I) really soft? Probing the hardness of Cu(I) with pyridinecarboxaldehyde ligands. Proc.–Indian Acad. Sci., Chem. Sci..

[cit46] Rizzi A. C., Neuman N. I., González P. J., Brondino C. D. (2016). EPR as a tool for study of isolated and coupled paramagnetic centers in coordination compounds and macromolecules of biological interest. Eur. J. Inorg. Chem..

[cit47] Rizzi A. C., Brondino C. D., Calvo R., Baggio R., Garland M. T., Rapp R. E. (2003). Structure and magnetic properties of layered high-spin Co(II)(L-threonine)_2_(H_2_O)_2_. Inorg. Chem..

[cit48] Rosa V., Gonzalez P. J., Avilés T., Gomes P. T., Welter R., Rizzi A. C., Passeggi M. C. G., Brondino C. D. (2006). Synthesis, solid-state structures, and EPR spectroscopic studies on polycrystalline and single-crystal samples of α-diimine cobalt(II) complexes. Eur. J. Inorg. Chem..

[cit49] Tamayo A., Casabó J., Escriche L., González P., Lodeiro C., Rizzi A. C., Brondino C. D., Passeggi M. C. G., Kivekäs R., Sillanpää R. (2007). Structural and EPR studies on single-crystal and polycrystalline samples of copper(II) and cobalt(II) complexes with N2S2-based macrocyclic ligands. Inorg. Chem..

[cit50] Neuman N. I., Winkler E., Peña O., Passeggi M. C. G., Rizzi A. C., Brondino C. D. (2014). Magnetic properties of weakly exchange-coupled high spin Co(II) ions in pseudooctahedral coordination evaluated by single crystal X-band EPR spectroscopy and magnetic measurements. Inorg. Chem..

[cit51] Mothilal K. K., Karunakaran C., Sambasiva Rao P., Murugesan R. (2003). Single crystal EPR of Cu(II) doped [Co(tbz)2(NO3)(H2O)]NO3: Probe into copper-thiabendazole interaction. Spectrochim. Acta, Part A.

[cit52] Goñi A., Lezama L. M., Rojo T., Foglio M. E., Valdivia J. A., Barberis G. E. (1998). ESR of Co^2+^ in (NH_4_)_2_NiCo1–(SO_4_)_2_·6H_2_O. Phys. Rev. B: Condens. Matter Mater. Phys..

[cit53] Gun’ko V. M., Savina I. N., Mikhalovsky S. V. (2013). Cryogels: Morphological, structural and adsorption characterisation. Adv. Colloid Interface Sci..

[cit54] Travlou N. A., Algarra M., Alcoholado C., Cifuentes-Rueda M., Labella A. M., Lazaro-Martínez J. M., Rodríguez-Castellon E., Bandosz T. J. (2018). Carbon quantum dot surface-chemistry-dependent ag release governs the high antibacterial activity of Ag-metal-organic framework composites. ACS Appl. Bio Mater..

[cit55] Gul-E-Noor F., Jee B., Pöppl A., Hartmann M., Himsl D., Bertmer M. (2011). Effects of varying water adsorption on a Cu3(BTC)2 metal-organic framework (MOF) as studied by 1H and 13C solid-state NMR spectroscopy. Phys. Chem. Chem. Phys..

[cit56] Gul-E-Noor F., Michel D., Krautscheid H., Haase J., Bertmer M. (2013). Time dependent water uptake in Cu_3_(btc)_2_ MOF: Identification of different water adsorption states by ^1^H MAS NMR. Microporous Mesoporous Mater..

[cit57] Ramamoorthy A., Xu J. (2013). 2D 1H/1H RFDR and NOESY NMR experiments on a membrane-bound antimicrobial peptide under magic angle spinning. J. Phys. Chem. B.

[cit58] Koval I. A., Der Van Schilden K., Schuitema A. M., Gamez P., Belle C., Pierre J. L., Luken M., Krebs B., Roubeau O., Reedijk J. (2005). Proton NMR spectroscopy and magnetic properties of a solution-stable dicopper(II) complex bearing a single μ-hydroxo bridge. Inorg. Chem..

[cit59] Baum T. H., Larson C. E., May G. (1992). Ligand-stabilized copper(I) hexafluoroacetylacetonate complexes: NMR spectroscope and the nature of the copper-alkene bond. J. Organomet. Chem..

[cit60] Novotný J., Sojka M., Komorovsky S., Nečas M., Marek R. (2016). Interpreting the Paramagnetic NMR Spectra of Potential Ru(III) Metallodrugs: Synergy between Experiment and Relativistic DFT Calculations. J. Am. Chem. Soc..

[cit61] Stoll S., Schweiger A. (2006). EasySpin, a comprehensive software package for spectral simulation and analysis in EPR. J. Magn. Reson..

[cit62] Fung B. M., Khitrin A. K., Ermolaev K. (2000). An improved broadband decoupling sequence for liquid crystals and solids. J. Magn. Reson..

[cit63] van Rossum B.-J., Förster H., de Groot H. J. M. (1997). High-Field and High-Speed CP-MAS 13C NMR Heteronuclear Dipolar-Correlation Spectroscopy of Solids with Frequency-Switched Lee–Goldburg Homonuclear Decoupling. J. Magn. Reson..

[cit64] Feike M., Demco D. E., Graf R., Gottwald J., Hafner S., Spiess H. W. (1996). Broadband multiple-quantum NMR spectroscopy. J. Magn. Reson., Ser. A.

[cit65] Frisch G. E. M. J. , Trucks G. W., Schlegel H. B., Scuseria V. B., Robb M. A., Cheeseman J. R., Scalmani G., Mennucci M. C. B., Petersson G. A., Nakatsuji H., Li G. X., Hratchian H. P., Izmaylov A. F., Bloino J., Zheng R., Sonnenberg J. L., Hada M., Ehara M., Toyota K., Fukuda O. K., Hasegawa J., Ishida M., Nakajima T., Honda Y., Nakai F. H., Vreven T., Montgomery Jr J. A., Peralta J. E., Ogliaro V. N., Bearpark M., Heyd J. J., Brothers E., Kudin K. N., Staroverov K. R., Keith T., Kobayashi R., Normand J., Rendell N. A., Burant J. C., Iyengar S. S., Tomasi J., Cossi M., Rega V. B., Millam J. M., Klene M., Knox J. E., Cross J. B., Adamo O. Y. C., Jaramillo J., Gomperts R., Stratmann R. E., Austin R. L. A. J., Cammi R., Pomelli C., Ochterski J. W., Martin P. S., Morokuma K., Zakrzewski V. G., Voth G. A., Dannenberg J. B. J. J., Dapprich S., Daniels A. D., Farkas O., Foresman D. J. F., Ortiz J. V. and Cioslowski J., Gaussian09, Revision E.01, Gaussian Inc. Wallingford CT, 2013

[cit66] Becke A. D. (1993). Density-functional thermochemistry. III. The role of exact exchange. J. Chem. Phys..

[cit67] Lee C., Yang W., Parr R. G. (1988). Development of the Colic-Salvetti correlation-energy formula into a functional of the electron density. Phys. Rev. B: Condens. Matter Mater. Phys..

[cit68] Hariharan P. C., Pople J. A. (1973). The influence of polarization functions on molecular orbital hydrogenation energies. Theor. Chim. Acta.

[cit69] Hehre W. J., Ditchfield K., Pople J. A. (1972). Self-consistent molecular orbital methods. XII. Further extensions of gaussian-type basis sets for use in molecular orbital studies of organic molecules. J. Chem. Phys..

[cit70] Becke A. D., Johnson E. R. (2005). Exchange-hole dipole moment and the dispersion interaction. J. Chem. Phys..

[cit71] Grimme S., Antony J., Ehrlich S., Krieg H. (2010). A consistent and accurate *ab initio* parametrization of density functional dispersion correction (DFT-D) for the 94 elements H-Pu. J. Chem. Phys..

[cit72] Johnson E. R., Becke A. D. (2006). A post-Hartree-Fock model of intermolecular interactions: Inclusion of higher-order corrections. J. Chem. Phys..

[cit73] Hay P. J. (1977). Gaussian basis sets for molecular calculations. The representation of 3d orbitals in transition-metal atoms. J. Chem. Phys..

[cit74] Wachters A. J. H. (1970). Gaussian Basis Set for Molecular Wavefunctions Containing Third-Row Atoms. J. Chem. Phys..

[cit75] Neese F. (2012). The ORCA program system. Wiley Interdiscip. Rev.: Comput. Mol. Sci..

[cit76] Neese F. (2018). Software update: the ORCA program system, version 4.0. Wiley Interdiscip. Rev.: Comput. Mol. Sci..

[cit77] Munzarova M., Kaupp M. (1999). A critical validation of density functional and coupled-cluster approaches for the calculation of EPR hyperfine coupling constants in transition metal complexes. J. Phys. Chem. A.

[cit78] Toomsalu E., Burk P. (2015). Critical test of some computational methods for prediction of NMR 1H and 13C chemical shifts. J. Mol. Model..

[cit79] Arbuznikov A. V., Kaupp M., Malkin V. G., Reviakine R., Malkina O. L. (2002). Validation study of meta-GGA functionals and of a model exchange-correlation potential in density functional calculations of EPR parameters. Phys. Chem. Chem. Phys..

[cit80] Golubeva E. N., Gromov O. I., Zhidomirov G. M. (2011). Cu(II)-alkyl chlorocomplexes: Stable compounds or transients? DFT prediction of their structure and EPR parameters. J. Phys. Chem. A.

[cit81] Hrobárik P., Reviakine R., Arbuznikov A. V., Malkina O. L., Malkin V. G., Köhler F. H., Kaupp M. (2007). Density functional calculations of NMR shielding tensors for paramagnetic systems with arbitrary spin multiplicity: Validation on 3d metallocenes. J. Chem. Phys..

[cit82] Jamróz M. K., Bak J., Gliński J. A., Koczorowska A., Wawer I. (2009). Molecular structure of actein: 13C CPMAS NMR, IR, X-ray diffraction studies and theoretical DFT-GIAO calculations. J. Mol. Struct..

[cit83] Kujawski J., Doskocz M., Popielarska H., Myka A., Drabinska B., Kruk J., Bernard M. K. (2013). Interactions between indazole derivative and magnesium cations-NMR investigations and theoretical calculations. J. Mol. Struct..

[cit84] Mirzaei M., Hadipour N. L. (2008). Study of hydrogen bonds in N-methylacetamide by DFT calculations of oxygen, nitrogen, and hydrogen solid-state NMR parameters. Struct. Chem..

[cit85] Moon S., Patchkovskii S. (2005). First Principles Calculations of Paramagnetic NMR Shifts. Chem. Inf..

