# Supplementary material for: Paramagnetic solid-state NMR assignment and novel chemical conversion of the aldehyde group to dihydrogen ortho ester and hemiacetal moieties in copper(ii)- and cobalt(ii)-pyridinecarboxaldehyde complexes
Source: RSC Adv. 2021 Jun 9;11(33):20216–31. doi: 10.1039/d1ra02512k (PMC9033980; doi:10.1039/d1ra02512k)
Supplement: RA-011-D1RA02512K-s001 [file RA-011-D1RA02512K-s001.pdf]

Electronic Supplementary Material (ESI) for RSC Advances.
This journal is © The Royal Society of Chemistry 2021

‘Electronic Supplementary Information’

Paramagnetic solid-state NMR assignment and novel chemical conversion of aldehyde group to dihydrogen ortho ester and hemiacetal moieties in copper(II)- and cobalt(II)-pyridinecarboxaldehyde complexes

Ayelén F. Crespi,[a] Verónica M. Sánchez,[b,c] Daniel Vega,[c,d] Ana L. Pérez,[e] Carlos D. Brondino,[e] Yamila Garro Linck,[f] Paul Hodgkinson,[g] Enrique Rodríguez-Castellón[h] and Juan M. Lázaro-Martínez[a,*]

[a.] Universidad de Buenos Aires - CONICET, Facultad de Farmacia y Bioquímica, Instituto de Química y Metabolismo del Fármaco (IQUIMEFA). Ciudad Autónoma de Buenos Aires, Argentina. E-mail: lazarojm@ffyb.uba.ar
[b.] Centro de Simulación Computacional para Aplicaciones Tecnológicas, CSC-CONICET, Ciudad Autónoma de Buenos Aires, Argentina.
[c.] Universidad Nacional de General San Martín, San Martín, Buenos Aires, Argentina.
[d.] Comisión Nacional de Energía Atómica, San Martín, Buenos Aires, Argentina.
[e.] Universidad Nacional del Litoral - CONICET, Facultad de Bioquímica y Ciencias Biológicas, Ciudad Universitaria, Santa Fe, Argentina.
[f.] Universidad Nacional de Córdoba, FaMAF & IFEG-CONICET, Córdoba, Argentina.
[g.] Durham University, Department of Chemistry, Durham, United Kingdom.
[h.] Universidad de Málaga, Facultad de Ciencias, Málaga, Spain.

Table of Contents:

Content	Page
1. Single-crystal X-ray results (4-pyridinecarboxaldehyde with $CuCl_2/CH_3OH$) (Figures S1 and S2; Tables S1-S4)	S3
2. Single-crystal X-ray results (4-pyridinecarboxaldehyde with $CoCl_2/CH_3OH$) (Figures S3 and S4; Tables S5-S8)	S16
3. Single-crystal X-ray results (3-pyridinecarboxaldehyde with $CoCl_2/CH_3OH$) (Figures S5 and S6; Tables S9-S12)	S28
4. Solution-state NMR results for the 4-pyridinecarboxaldehyde (Figures S7-S18)	S36
5. Solution-state NMR results for the 3-pyridinecarboxaldehyde (Figures S18-S24)	S45
6. Coordinates for the DFT calculations	S50
7. EPR parameters and its contribution to pNMR shielding tensor (Tables S13-S15 and Figures S25-S26)	S53
8. Solid-state NMR - 2D ^1H-^{13}C HETCOR spectra for the single crystals obtained with 4-pyridinecarboxaldehyde and $CuCl_2$ in methanol with different contact times (Figure S27)	S58

Single-crystal X-ray Diffraction Results:

***Cu(II)-4-pyridinecarboxaldehyde (CIF FILE: AC12_100K_2_0m, CCDC 2047187)**

Crystal system is trigonal, R-3, unit cell a: 24.4486(3), c: 29.1012(8) Å. 50978 reflections were collected, 6886 independents, Rint: 0.056. The structure was solved using program SHELXS-97[1] and refined using the full-matrix LS procedure with SHELXL-2014/7.[2] Anisotropic displacement parameters were employed for non-hydrogen atoms. All H atoms were located at the expected positions and they were refined using a riding model. H atoms for water molecule were located in the Fourier difference density map. LS weights of the form $w=1/[\sigma^2(F_o^2) + (0.1586\ P)^2 + 154.4517\ P]$ where $P = (F_o^2 + 2F_c^2)/3$, were employed. $R[F^2>2\sigma(F^2)] = 0.0857$, $wR(F^2) = 0.2445$. One of the terminal R-CH(OH)(OCH3) hemiacetal group was modeled as two disordered parts (O8C1,O9C,C1C1 and O8C2,O10C,C1C2 as part 1 and 2 respectively) refined with isotropic thermal parameters. Solvent molecules located in channels were also refined with isotropic thermal parameters.

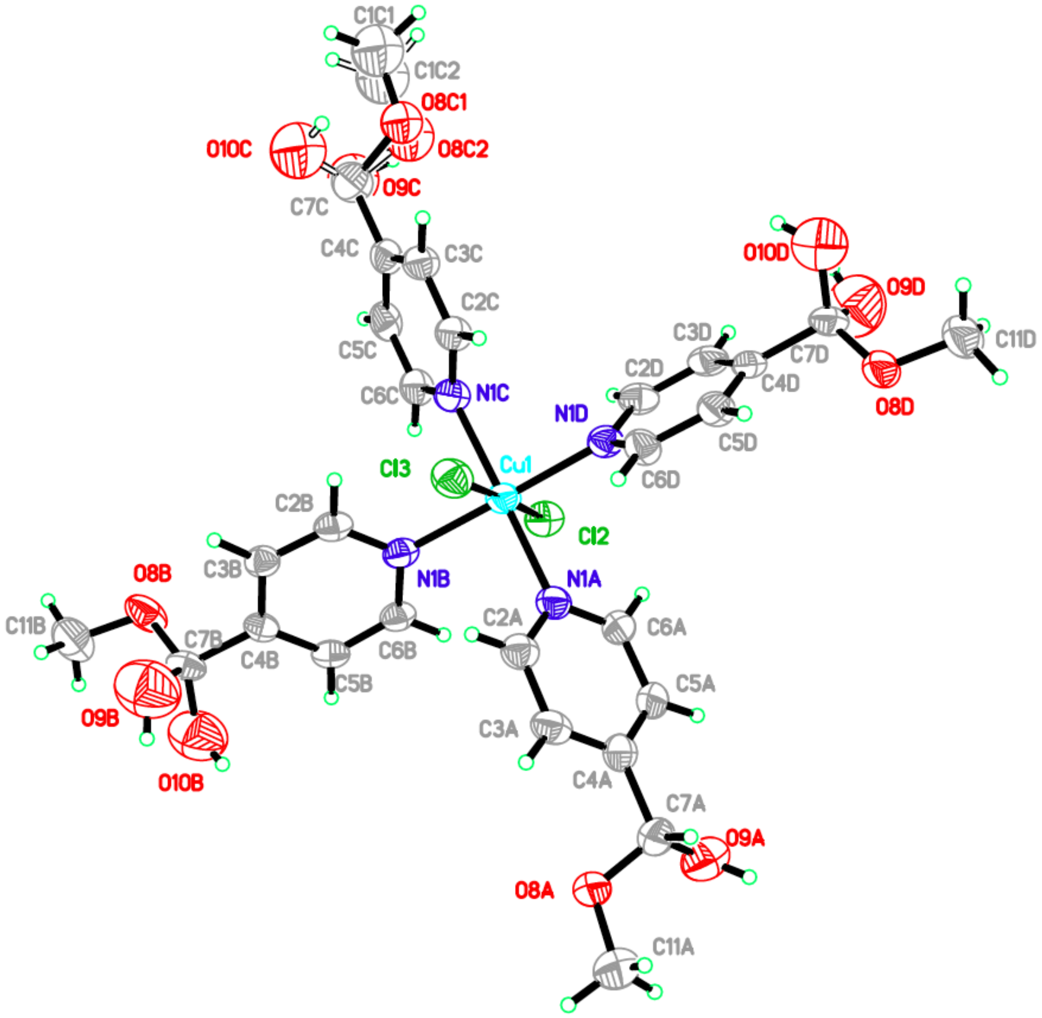

Figure S1. Crystal structure and numbering scheme for the copper complex for the 4-pyridinecarboxaldehyde with $CuCl_2/CH_3OH$. The displacement ellipsoids for the non-H atoms in the figure were drawn at the 50% probability level.

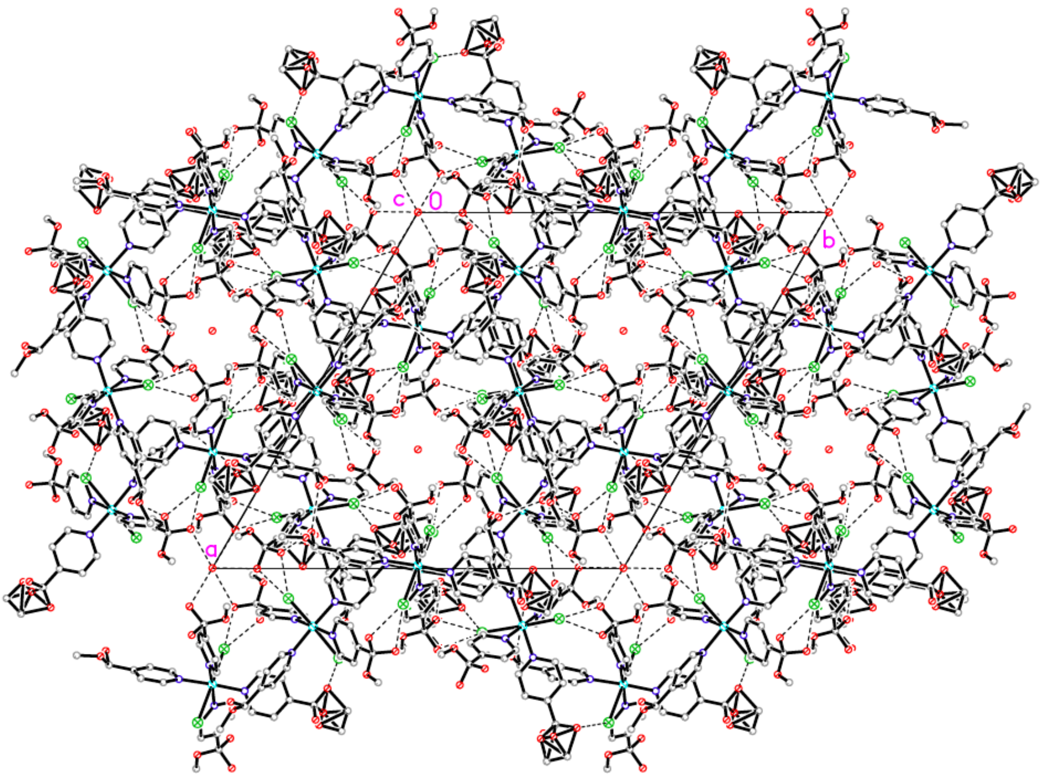

Figure S2. Crystal packing structure for the copper complex for the 4-pyridinecarboxaldehyde with $CuCl_2/CH_3OH$. The displacement ellipsoids for the non-H atoms in the figure were drawn at the 50% probability level.

Table S1. Crystal data and structure refinement for 4-pyridinecarboxaldehyde with $CuCl_2/CH_3OH$ (**AC12_100K_2_0m)**

Identification code	**AC12_100K_2_0m (CCDC 2047187)**
Empirical formula	C28 H35 Cl2 Cu N4 O11
Formula weight	738.04
Temperature	100(2) K
Wavelength	0.71073 Å

Crystal system	Trigonal	
Space group	R -3 :H	
Unit cell dimensions	a = 24.4486(3) Å	α= 90°.
	b = 24.4486(3) Å	β= 90°.
	c = 29.1012(8) Å	γ= 120°.
Volume	15064.3(6) Å^3	
Z	18	
Density (calculated)	1.464 Mg/m^3	
Absorption coefficient	0.874 mm^{-1}	
F(000)	6876	
Crystal size	0.5 x 0.5 x 0.3 mm^3	
Theta range for data collection	2.099 to 26.423°.	
Index ranges	-30<=h<=30, -30<=k<=30, -36<=l<=36	
Reflections collected	50978	
Independent reflections	6886 [R(int) = 0.0562]	
Completeness to theta = 25.242°	99.9 %	
Refinement method	Full-matrix least-squares on F^2	
Data / restraints / parameters	6886 / 0 / 417	
Goodness-of-fit on F^2	1.052	
Final R indices [I>2sigma(I)]	R1 = 0.0859, wR2 = 0.2450	
R indices (all data)	R1 = 0.1076, wR2 = 0.2739	
Extinction coefficient	n/a	
Largest diff. peak and hole	1.799 and -1.177 e.Å^{-3}	

Table S2. Bond lengths [Å] and angles [°] for 4-pyridinecarboxaldehyde with $CuCl_2/CH_3OH$ (**AC12_100K_2_0m)**

Cu(1)-N(1B)	2.018(4)
Cu(1)-N(1D)	2.025(4)
Cu(1)-N(1C)	2.033(4)
Cu(1)-N(1A)	2.038(4)
Cu(1)-Cl(2)	2.6922(15)

N(1A)-C(2A)	1.340(7)
N(1A)-C(6A)	1.341(7)
C(2A)-C(3A)	1.380(7)
C(2A)-H(2A)	0.9500
O(1W)-O(3W)	1.354(12)
C(3A)-C(4A)	1.397(8)
C(3A)-H(3A)	0.9500
C(4A)-C(5A)	1.376(8)
C(4A)-C(7A)	1.522(8)
C(5A)-C(6A)	1.385(7)
C(5A)-H(5A)	0.9500
C(6A)-H(6A)	0.9500
C(7A)-O(8A)	1.379(7)
C(7A)-O(9A)	1.407(8)
C(7A)-H(7A)	1.0000
O(8A)-C(11A)	1.415(8)
O(9A)-H(9A)	0.8400
C(11A)-H(8A1)	0.9800
C(11A)-H(8A2)	0.9800
C(11A)-H(8A3)	0.9800
N(1B)-C(2B)	1.343(7)
N(1B)-C(6B)	1.348(6)
C(2B)-C(3B)	1.374(7)
C(2B)-H(2B)	0.9500
C(3B)-C(4B)	1.392(6)
C(3B)-H(3B)	0.9500
C(4B)-C(5B)	1.386(7)
C(4B)-C(7B)	1.505(7)
C(5B)-C(6B)	1.377(7)
C(5B)-H(5B)	0.9500
C(6B)-H(6B)	0.9500
C(7B)-O(9B)	1.341(9)
C(7B)-O(10B)	1.365(8)
C(7B)-O(8B)	1.385(6)
O(8B)-C(11B)	1.419(8)
O(9B)-H(9B)	0.8400
O(10B)-H(10)	0.8400
C(11B)-H(8B1)	0.9800
C(11B)-H(8B2)	0.9800
C(11B)-H(8B3)	0.9800

N(1C)-C(2C)	1.327(6)
N(1C)-C(6C)	1.352(6)
C(2C)-C(3C)	1.375(7)
C(2C)-H(2C)	0.9500
C(3C)-C(4C)	1.388(7)
C(3C)-H(3C)	0.9500
C(4C)-C(5C)	1.393(8)
C(4C)-C(7C)	1.511(8)
C(5C)-C(6C)	1.360(7)
C(5C)-H(5C)	0.9500
C(6C)-H(6C)	0.9500
C(7C)-O(8C2)	1.325(15)
C(7C)-O(9C)	1.356(13)
C(7C)-O(10C)	1.436(18)
C(7C)-O(8C1)	1.437(10)
N(1D)-C(6D)	1.336(6)
N(1D)-C(2D)	1.336(6)
C(2D)-C(3D)	1.369(8)
C(2D)-H(2D)	0.9500
C(3D)-C(4D)	1.367(7)
C(3D)-H(3D)	0.9500
C(4D)-C(5D)	1.389(7)
C(4D)-C(7D)	1.514(7)
C(5D)-C(6D)	1.385(7)
C(5D)-H(5D)	0.9500
C(6D)-H(6D)	0.9500
C(7D)-O(8D)	1.364(6)
C(7D)-O(9D)	1.396(9)
C(7D)-O(10D)	1.397(9)
O(8D)-C(11D)	1.426(7)
O(9D)-H(9D4)	0.8400
O(10D)-H(8D5)	0.8400
C(11D)-H(8D1)	0.9800
C(11D)-H(8D2)	0.9800
C(11D)-H(8D3)	0.9800
O(9C)-H(9C1)	0.8400
O(10C)-H(10C)	0.8400
C(1C1)-O(8C1)	1.403(18)
C(1C1)-H(1CA)	0.9800
C(1C1)-H(1CB)	0.9800

C(1C1)-H(1CC)	0.9800
C(1C2)-O(8C2)	1.48(3)
C(1C2)-H(1CD)	0.9800
C(1C2)-H(1CE)	0.9800
C(1C2)-H(1CF)	0.9800
N(1B)-Cu(1)-N(1D)	176.00(16)
N(1B)-Cu(1)-N(1C)	89.92(16)
N(1D)-Cu(1)-N(1C)	89.94(16)
N(1B)-Cu(1)-N(1A)	89.84(16)
N(1D)-Cu(1)-N(1A)	90.06(16)
N(1C)-Cu(1)-N(1A)	176.56(18)
N(1B)-Cu(1)-Cl(2)	92.69(12)
N(1D)-Cu(1)-Cl(2)	91.32(12)
N(1C)-Cu(1)-Cl(2)	92.12(13)
N(1A)-Cu(1)-Cl(2)	91.32(13)
C(2A)-N(1A)-C(6A)	117.9(4)
C(2A)-N(1A)-Cu(1)	120.6(4)
C(6A)-N(1A)-Cu(1)	121.4(3)
N(1A)-C(2A)-C(3A)	122.5(5)
N(1A)-C(2A)-H(2A)	118.8
C(3A)-C(2A)-H(2A)	118.8
C(2A)-C(3A)-C(4A)	119.6(5)
C(2A)-C(3A)-H(3A)	120.2
C(4A)-C(3A)-H(3A)	120.2
C(5A)-C(4A)-C(3A)	117.6(5)
C(5A)-C(4A)-C(7A)	121.4(5)
C(3A)-C(4A)-C(7A)	120.9(5)
O(1W)#1-O(3W)-O(1W)	180.0
C(4A)-C(5A)-C(6A)	119.6(5)
C(4A)-C(5A)-H(5A)	120.2
C(6A)-C(5A)-H(5A)	120.2
N(1A)-C(6A)-C(5A)	122.8(5)
N(1A)-C(6A)-H(6A)	118.6
C(5A)-C(6A)-H(6A)	118.6
O(8A)-C(7A)-O(9A)	113.0(5)
O(8A)-C(7A)-C(4A)	106.6(4)
O(9A)-C(7A)-C(4A)	112.7(5)
O(8A)-C(7A)-H(7A)	108.1
O(9A)-C(7A)-H(7A)	108.1
C(4A)-C(7A)-H(7A)	108.1

C(7A)-O(8A)-C(11A)	112.7(5)
C(7A)-O(9A)-H(9A)	109.5
O(8A)-C(11A)-H(8A1)	109.5
O(8A)-C(11A)-H(8A2)	109.5
H(8A1)-C(11A)-H(8A2)	109.5
O(8A)-C(11A)-H(8A3)	109.5
H(8A1)-C(11A)-H(8A3)	109.5
H(8A2)-C(11A)-H(8A3)	109.5
C(2B)-N(1B)-C(6B)	117.9(4)
C(2B)-N(1B)-Cu(1)	121.2(3)
C(6B)-N(1B)-Cu(1)	120.9(3)
N(1B)-C(2B)-C(3B)	123.2(4)
N(1B)-C(2B)-H(2B)	118.4
C(3B)-C(2B)-H(2B)	118.4
C(2B)-C(3B)-C(4B)	118.7(4)
C(2B)-C(3B)-H(3B)	120.7
C(4B)-C(3B)-H(3B)	120.7
C(5B)-C(4B)-C(3B)	118.4(4)
C(5B)-C(4B)-C(7B)	122.0(4)
C(3B)-C(4B)-C(7B)	119.7(4)
C(6B)-C(5B)-C(4B)	119.6(4)
C(6B)-C(5B)-H(5B)	120.2
C(4B)-C(5B)-H(5B)	120.2
N(1B)-C(6B)-C(5B)	122.2(5)
N(1B)-C(6B)-H(6B)	118.9
C(5B)-C(6B)-H(6B)	118.9
O(9B)-C(7B)-O(10B)	98.7(5)
O(9B)-C(7B)-O(8B)	113.5(6)
O(10B)-C(7B)-O(8B)	116.8(5)
O(9B)-C(7B)-C(4B)	109.8(5)
O(10B)-C(7B)-C(4B)	111.3(5)
O(8B)-C(7B)-C(4B)	106.6(4)
C(7B)-O(8B)-C(11B)	113.3(4)
C(7B)-O(9B)-H(9B)	109.5
C(7B)-O(10B)-H(10)	109.5
O(8B)-C(11B)-H(8B1)	109.5
O(8B)-C(11B)-H(8B2)	109.5
H(8B1)-C(11B)-H(8B2)	109.5
O(8B)-C(11B)-H(8B3)	109.5
H(8B1)-C(11B)-H(8B3)	109.5

H(8B2)-C(11B)-H(8B3)	109.5
C(2C)-N(1C)-C(6C)	117.8(4)
C(2C)-N(1C)-Cu(1)	121.4(3)
C(6C)-N(1C)-Cu(1)	120.8(3)
N(1C)-C(2C)-C(3C)	123.0(4)
N(1C)-C(2C)-H(2C)	118.5
C(3C)-C(2C)-H(2C)	118.5
C(2C)-C(3C)-C(4C)	119.5(5)
C(2C)-C(3C)-H(3C)	120.2
C(4C)-C(3C)-H(3C)	120.2
C(3C)-C(4C)-C(5C)	117.2(5)
C(3C)-C(4C)-C(7C)	120.8(5)
C(5C)-C(4C)-C(7C)	122.0(5)
C(6C)-C(5C)-C(4C)	119.9(5)
C(6C)-C(5C)-H(5C)	120.0
C(4C)-C(5C)-H(5C)	120.0
N(1C)-C(6C)-C(5C)	122.5(5)
N(1C)-C(6C)-H(6C)	118.7
C(5C)-C(6C)-H(6C)	118.7
O(8C2)-C(7C)-O(10C)	114.0(11)
O(9C)-C(7C)-O(8C1)	108.3(7)
O(8C2)-C(7C)-C(4C)	107.3(8)
O(9C)-C(7C)-C(4C)	111.1(7)
O(10C)-C(7C)-C(4C)	110.7(8)
O(8C1)-C(7C)-C(4C)	105.3(5)
C(6D)-N(1D)-C(2D)	118.2(4)
C(6D)-N(1D)-Cu(1)	120.4(3)
C(2D)-N(1D)-Cu(1)	121.4(3)
N(1D)-C(2D)-C(3D)	122.8(5)
N(1D)-C(2D)-H(2D)	118.6
C(3D)-C(2D)-H(2D)	118.6
C(4D)-C(3D)-C(2D)	119.6(5)
C(4D)-C(3D)-H(3D)	120.2
C(2D)-C(3D)-H(3D)	120.2
C(3D)-C(4D)-C(5D)	118.3(5)
C(3D)-C(4D)-C(7D)	119.5(4)
C(5D)-C(4D)-C(7D)	122.2(5)
C(6D)-C(5D)-C(4D)	119.1(5)
C(6D)-C(5D)-H(5D)	120.5
C(4D)-C(5D)-H(5D)	120.5

N(1D)-C(6D)-C(5D)	122.0(4)
N(1D)-C(6D)-H(6D)	119.0
C(5D)-C(6D)-H(6D)	119.0
O(8D)-C(7D)-O(9D)	111.5(5)
O(8D)-C(7D)-O(10D)	112.7(5)
O(9D)-C(7D)-O(10D)	104.9(6)
O(8D)-C(7D)-C(4D)	107.3(4)
O(9D)-C(7D)-C(4D)	109.7(5)
O(10D)-C(7D)-C(4D)	110.7(5)
C(7D)-O(8D)-C(11D)	114.2(4)
C(7D)-O(9D)-H(9D4)	109.5
C(7D)-O(10D)-H(8D5)	109.5
O(8D)-C(11D)-H(8D1)	109.5
O(8D)-C(11D)-H(8D2)	109.5
H(8D1)-C(11D)-H(8D2)	109.5
O(8D)-C(11D)-H(8D3)	109.5
H(8D1)-C(11D)-H(8D3)	109.5
H(8D2)-C(11D)-H(8D3)	109.5
C(7C)-O(9C)-H(9C1)	109.5
C(7C)-O(10C)-H(10C)	109.5
O(8C1)-C(1C1)-H(1CA)	109.5
O(8C1)-C(1C1)-H(1CB)	109.5
H(1CA)-C(1C1)-H(1CB)	109.5
O(8C1)-C(1C1)-H(1CC)	109.5
H(1CA)-C(1C1)-H(1CC)	109.5
H(1CB)-C(1C1)-H(1CC)	109.5
C(1C1)-O(8C1)-C(7C)	110.8(9)
O(8C2)-C(1C2)-H(1CD)	109.5
O(8C2)-C(1C2)-H(1CE)	109.5
H(1CD)-C(1C2)-H(1CE)	109.5
O(8C2)-C(1C2)-H(1CF)	109.5
H(1CD)-C(1C2)-H(1CF)	109.5
H(1CE)-C(1C2)-H(1CF)	109.5
C(7C)-O(8C2)-C(1C2)	109.9(14)

Symmetry transformations used to generate equivalent atoms:

#1 -x+2/3,-y+4/3,-z+1/3

Table S3. Torsion angles [°] for 4-pyridinecarboxaldehyde with $CuCl_2/CH_3OH$ (**AC12_100K_2_0m)**

C(6A)-N(1A)-C(2A)-C(3A)	-0.3(7)
Cu(1)-N(1A)-C(2A)-C(3A)	177.5(4)
N(1A)-C(2A)-C(3A)-C(4A)	-1.0(8)
C(2A)-C(3A)-C(4A)-C(5A)	1.2(7)
C(2A)-C(3A)-C(4A)-C(7A)	-178.8(5)
C(3A)-C(4A)-C(5A)-C(6A)	-0.2(8)
C(7A)-C(4A)-C(5A)-C(6A)	179.9(5)
C(2A)-N(1A)-C(6A)-C(5A)	1.4(8)
Cu(1)-N(1A)-C(6A)-C(5A)	-176.4(4)
C(4A)-C(5A)-C(6A)-N(1A)	-1.2(8)
C(5A)-C(4A)-C(7A)-O(8A)	-143.2(5)
C(3A)-C(4A)-C(7A)-O(8A)	36.8(7)
C(5A)-C(4A)-C(7A)-O(9A)	-18.6(7)
C(3A)-C(4A)-C(7A)-O(9A)	161.4(5)
O(9A)-C(7A)-O(8A)-C(11A)	68.5(8)
C(4A)-C(7A)-O(8A)-C(11A)	-167.1(7)
C(6B)-N(1B)-C(2B)-C(3B)	-0.6(7)
Cu(1)-N(1B)-C(2B)-C(3B)	179.3(4)
N(1B)-C(2B)-C(3B)-C(4B)	0.8(7)
C(2B)-C(3B)-C(4B)-C(5B)	0.0(7)
C(2B)-C(3B)-C(4B)-C(7B)	179.9(4)
C(3B)-C(4B)-C(5B)-C(6B)	-0.9(7)
C(7B)-C(4B)-C(5B)-C(6B)	179.2(4)
C(2B)-N(1B)-C(6B)-C(5B)	-0.4(7)
Cu(1)-N(1B)-C(6B)-C(5B)	179.6(4)
C(4B)-C(5B)-C(6B)-N(1B)	1.2(7)
C(5B)-C(4B)-C(7B)-O(9B)	121.9(6)
C(3B)-C(4B)-C(7B)-O(9B)	-58.0(7)
C(5B)-C(4B)-C(7B)-O(10B)	13.6(7)
C(3B)-C(4B)-C(7B)-O(10B)	-166.3(5)
C(5B)-C(4B)-C(7B)-O(8B)	-114.8(5)
C(3B)-C(4B)-C(7B)-O(8B)	65.3(5)
O(9B)-C(7B)-O(8B)-C(11B)	-55.9(7)
O(10B)-C(7B)-O(8B)-C(11B)	58.0(7)
C(4B)-C(7B)-O(8B)-C(11B)	-176.9(5)
C(6C)-N(1C)-C(2C)-C(3C)	-1.0(8)
Cu(1)-N(1C)-C(2C)-C(3C)	178.5(4)
N(1C)-C(2C)-C(3C)-C(4C)	-0.3(8)
C(2C)-C(3C)-C(4C)-C(5C)	0.7(8)

C(2C)-C(3C)-C(4C)-C(7C)	-179.4(5)
C(3C)-C(4C)-C(5C)-C(6C)	0.2(8)
C(7C)-C(4C)-C(5C)-C(6C)	-179.7(5)
C(2C)-N(1C)-C(6C)-C(5C)	1.9(8)
Cu(1)-N(1C)-C(6C)-C(5C)	-177.6(4)
C(4C)-C(5C)-C(6C)-N(1C)	-1.5(8)
C(3C)-C(4C)-C(7C)-O(8C2)	73.2(11)
C(5C)-C(4C)-C(7C)-O(8C2)	-106.9(10)
C(3C)-C(4C)-C(7C)-O(9C)	154.0(7)
C(5C)-C(4C)-C(7C)-O(9C)	-26.1(9)
C(3C)-C(4C)-C(7C)-O(10C)	-51.8(10)
C(5C)-C(4C)-C(7C)-O(10C)	128.0(9)
C(3C)-C(4C)-C(7C)-O(8C1)	37.0(8)
C(5C)-C(4C)-C(7C)-O(8C1)	-143.2(6)
C(6D)-N(1D)-C(2D)-C(3D)	0.4(7)
Cu(1)-N(1D)-C(2D)-C(3D)	-178.7(4)
N(1D)-C(2D)-C(3D)-C(4D)	0.0(8)
C(2D)-C(3D)-C(4D)-C(5D)	-0.4(7)
C(2D)-C(3D)-C(4D)-C(7D)	-179.9(5)
C(3D)-C(4D)-C(5D)-C(6D)	0.4(7)
C(7D)-C(4D)-C(5D)-C(6D)	179.9(5)
C(2D)-N(1D)-C(6D)-C(5D)	-0.5(8)
Cu(1)-N(1D)-C(6D)-C(5D)	178.7(4)
C(4D)-C(5D)-C(6D)-N(1D)	0.1(8)
C(3D)-C(4D)-C(7D)-O(8D)	-140.8(5)
C(5D)-C(4D)-C(7D)-O(8D)	39.7(7)
C(3D)-C(4D)-C(7D)-O(9D)	-19.5(7)
C(5D)-C(4D)-C(7D)-O(9D)	161.0(5)
C(3D)-C(4D)-C(7D)-O(10D)	95.8(6)
C(5D)-C(4D)-C(7D)-O(10D)	-83.7(7)
O(9D)-C(7D)-O(8D)-C(11D)	57.7(7)
O(10D)-C(7D)-O(8D)-C(11D)	-59.9(7)
C(4D)-C(7D)-O(8D)-C(11D)	177.9(5)
O(9C)-C(7C)-O(8C1)-C(1C1)	58.3(12)
C(4C)-C(7C)-O(8C1)-C(1C1)	177.2(9)
O(10C)-C(7C)-O(8C2)-C(1C2)	-76.6(17)
C(4C)-C(7C)-O(8C2)-C(1C2)	160.4(13)

Symmetry transformations used to generate equivalent atoms:

#1 -x+2/3,-y+4/3,-z+1/3

Table S4. Hydrogen bonds for 4-pyridinecarboxaldehyde with $CuCl_2/CH_3OH$ (**AC12_100K_2_0m)** [Å and °].

D-H...A	d(D-H)	d(H...A)	d(D...A)	<(DHA)
C(2A)-H(2A)...Cl(3)	0.95	2.75	3.365(5)	123.1
C(6A)-H(6A)...Cl(2)	0.95	2.83	3.368(5)	116.8
C(6A)-H(6A)...O(8C2^b)#2	0.95	2.47	3.279(16)	143.7
C(7A)-H(7A)...Cl(2)#2	1.00	2.92	3.868(6)	159.3
O(9A)-H(9A)...O(9B)#3	0.84	1.70	2.532(9)	169.5
C(11A)-H(8A2)...Cl(3)#4	0.98	2.89	3.826(8)	160.9
C(2B)-H(2B)...Cl(3)	0.95	2.87	3.396(5)	116.1
C(6B)-H(6B)...Cl(2)	0.95	2.94	3.450(5)	115.1
C(6B)-H(6B)...O(8B)#4	0.95	2.56	3.352(6)	140.6
O(9B)-H(9B)...Cl(2)#4	0.84	2.06	2.887(8)	167.8
O(10B)-H(10)...Cl(2)#4	0.84	2.69	3.151(7)	115.8
C(11B)-H(8B2)...Cl(2)#5	0.98	2.76	3.700(7)	161.8
C(2C)-H(2C)...Cl(3)	0.95	2.87	3.470(5)	121.9
C(2C)-H(2C)...O(8D)#6	0.95	2.63	3.300(6)	127.7
C(6C)-H(6C)...Cl(2)	0.95	2.85	3.388(5)	117.3
C(2D)-H(2D)...Cl(2)	0.95	2.76	3.340(5)	119.8
C(6D)-H(6D)...Cl(3)	0.95	2.76	3.409(5)	125.8
O(9D)-H(9D4)...Cl(3)#6	0.84	2.11	2.873(8)	151.6
C(11D)-H(8D2)...Cl(3)#2	0.98	2.88	3.785(7)	153.8
O(10C^b)-H(10C^b)...O(10C^b)#7 / 0.84		2.38	3.14(3)	150.2
C(1C2^b)-H(1CD^b)...Cl(2)#6	0.98	2.77	3.61(3)	144.5
C(1C2^b)-H(1CE^b)...O(9D)#8	0.98	2.53	3.40(3)	146.6

Symmetry transformations used to generate equivalent atoms:

#1 -x+2/3,-y+4/3,-z+1/3 #2 x-y+1,x+1,-z #3 -y+4/3,x-y+5/3,z-1/3

#4 y-1/3,-x+y+1/3,-z+1/3 #5 x-y+2/3,x+1/3,-z+1/3

#6 y-1,-x+y,-z #7 -x-1/3,-y+4/3,-z+1/3 #8 -y+2/3,x-y+4/3,z+1/3

***Co(II)-4-pyridinecarboxaldehyde (CIF FILE: AC35_2_0m, CCDC 2047188)**

Crystal system is trigonal, R-3, unit cell a: 24.0755(8), c: 29.5779(10) Å. 64451 reflections were collected, 6727 independents, Rint: 0.030. The structure was solved using program SHELXS-97[1] and refined using the full-matrix LS procedure with SHELXL-2014/7.[2] Anisotropic displacement parameters were employed for non-hydrogen atoms. All H atoms were located at the expected positions and they were refined using a riding model. H atoms for water molecule were located in the Fourier difference density map. LS weights of the form $w=1/[\sigma^2(F_o^2) + (0.1514\ P)^2 + 199.56\ P]$ where $P = (F_o^2 + 2F_c^2)/3$, were employed. $R[F^2>2\sigma(F^2)] = 0.1016$, $wR(F^2) = 0.2669$. Solvent molecules located in channels were refined with isotropic thermal parameters.

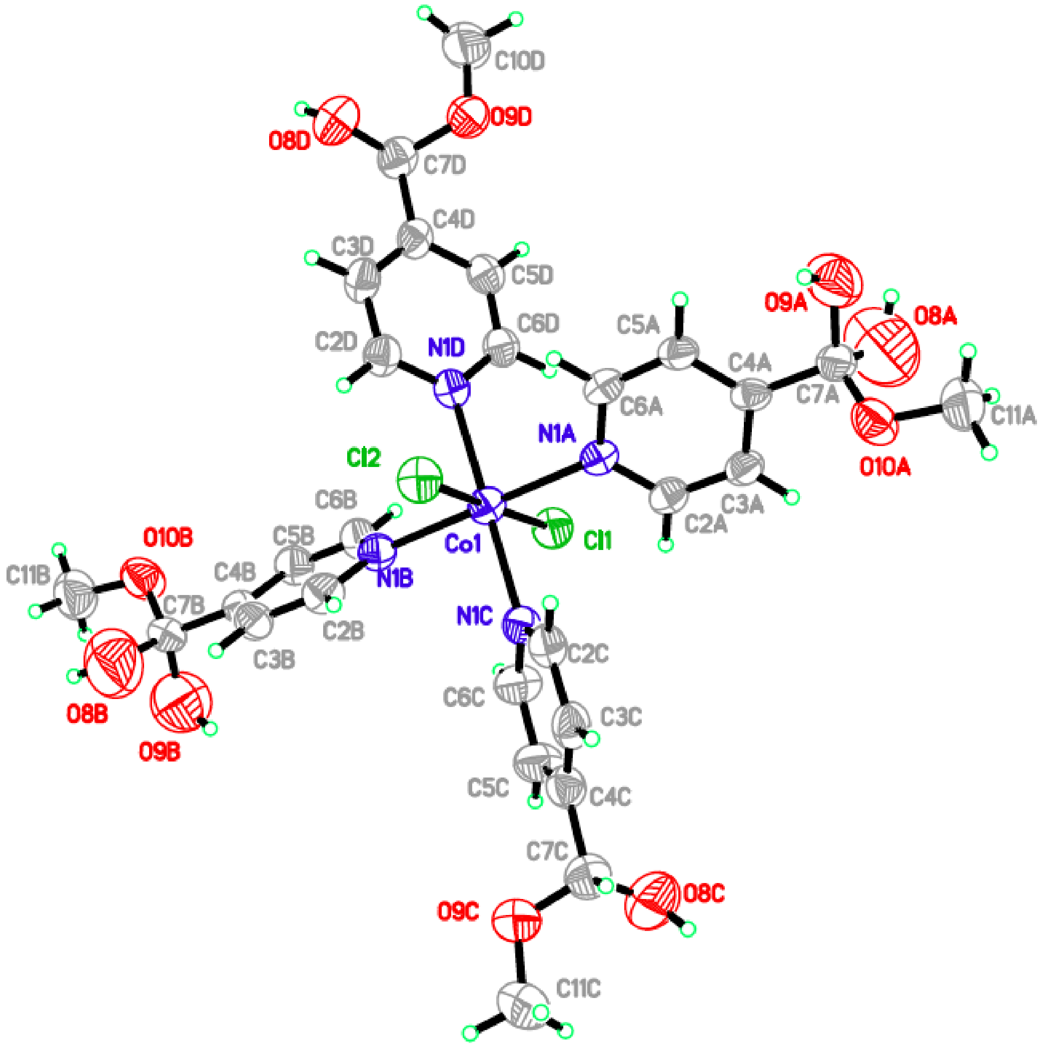

Figure S3. Crystal structure and numbering scheme for the cobalt complex for the 4-pyridinecarboxaldehyde with $CoCl_2/CH_3OH$. The displacement ellipsoids for the non-H atoms in the figure were drawn at the 50% probability level.

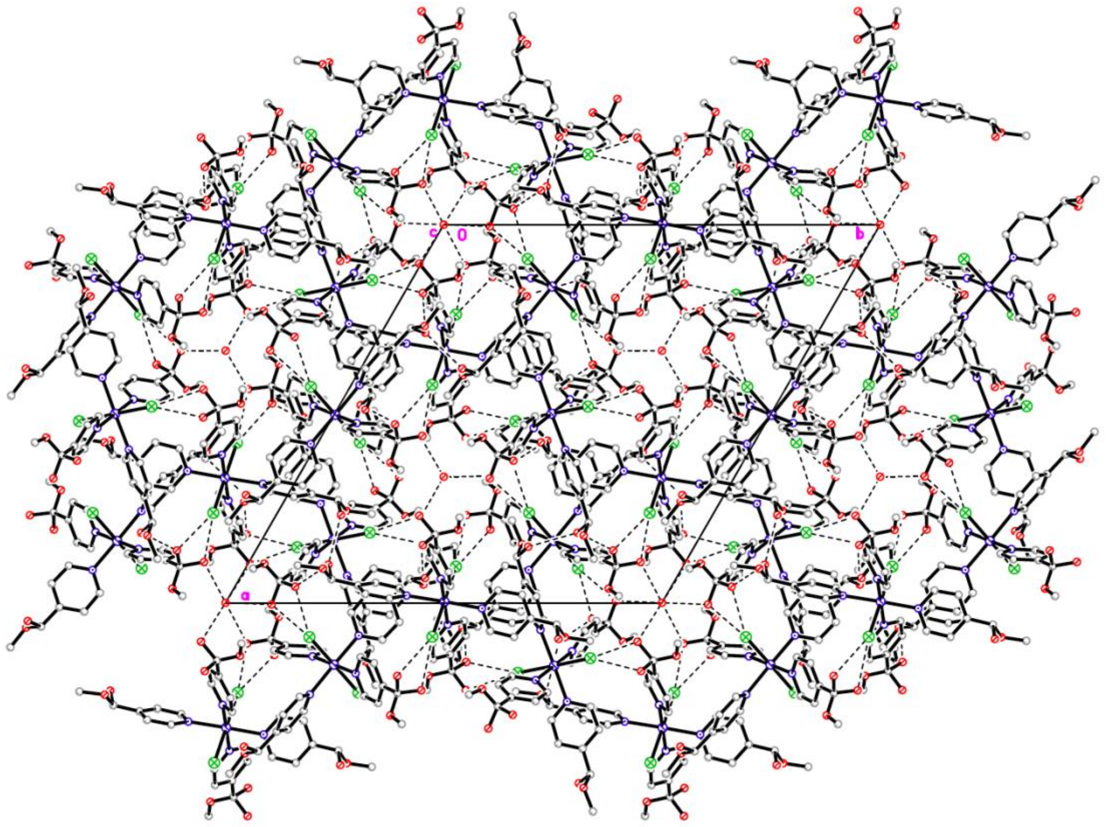

Figure S4. Crystal packing structure for the cobalt complex for the 4-pyridinecarboxaldehyde with $CoCl_2/CH_3OH$. The displacement ellipsoids for the non-H atoms in the figure were drawn at the 50% probability level.

Table S5. Crystal data and structure refinement for 4-pyridinecarboxaldehyde with $CoCl_2/CH_3OH$ (**AC35_2_0m)**

Identification code	**AC35_2_0m (CCDC 2047188)**	
Empirical formula	C28 H36 Cl2 Co N4 O10.92	
Formula weight	733.10	
Temperature	100(2) K	
Wavelength	0.71073 Å	
Crystal system	Trigonal	
Space group	R -3 :H	
Unit cell dimensions	a = 24.0755(8) Å	α= 90°.
	b = 24.0755(8) Å	β= 90°.
	c = 29.5779(10) Å	γ= 120°.
Volume	14847.3(11) $Å^3$	
Z	18	
Density (calculated)	1.476 Mg/m^3	
Absorption coefficient	0.745 mm^{-1}	
F(000)	6846	
Crystal size	0.4 x 0.4 x 0.2 mm^3	
Theta range for data collection	2.066 to 26.403°.	
Index ranges	-30<=h<=30, -30<=k<=30, -36<=l<=36	
Reflections collected	64451	
Independent reflections	6727 [R(int) = 0.0300]	
Completeness to theta = 25.242°	99.3 %	
Refinement method	Full-matrix least-squares on F^2	
Data / restraints / parameters	6727 / 0 / 417	
Goodness-of-fit on F^2	1.052	
Final R indices [I>2sigma(I)]	R1 = 0.1016, wR2 = 0.2669	
R indices (all data)	R1 = 0.1291, wR2 = 0.3032	
Extinction coefficient	n/a	
Largest diff. peak and hole	1.115 and -1.156 e.$Å^{-3}$	

Table S6. Bond lengths [pm] and angles [°] for 4-pyridinecarboxaldehyde with $CoCl_2/CH_3OH$ (**AC35_2_0m**).

Co(1)-N(1A)	2.146(4)
Co(1)-N(1B)	2.158(4)
Co(1)-N(1C)	2.159(4)
Co(1)-N(1D)	2.174(4)
Co(1)-Cl(2)	2.4500(15)
Co(1)-Cl(1)	2.5023(14)
N(1A)-C(2A)	1.340(6)
N(1A)-C(6A)	1.346(6)
C(2A)-C(3A)	1.377(8)
C(2A)-H(30)	0.9500
C(3A)-C(4A)	1.386(7)
C(3A)-H(35)	0.9500
C(4A)-C(5A)	1.388(7)
C(4A)-C(7A)	1.505(8)
C(5A)-C(6A)	1.375(7)
C(5A)-H(5A)	0.9500
C(6A)-H(34)	0.9500
C(7A)-O(8A)	1.313(12)
C(7A)-O(10A)	1.362(7)
C(7A)-O(9A)	1.372(8)
O(8A)-H(8A)	0.8400
O(9A)-H(9A)	0.8400
O(10A)-C(11A)	1.422(8)
C(11A)-H(32)	0.9800
C(11A)-H(33)	0.9800
C(11A)-H(31)	0.9800
N(1B)-C(2B)	1.341(7)
N(1B)-C(6B)	1.342(7)
C(2B)-C(3B)	1.371(8)
C(2B)-H(2B)	0.9500
C(3B)-C(4B)	1.371(8)
C(3B)-H(21)	0.9500
C(4B)-C(5B)	1.387(7)
C(4B)-C(7B)	1.515(8)
C(5B)-C(6B)	1.376(8)
C(5B)-H(29)	0.9500
C(6B)-H(19)	0.9500
C(7B)-O(8B)	1.332(11)

C(7B)-O(9B)	1.344(10)
C(7B)-O(10B)	1.351(7)
O(8B)-H(8B)	0.8400
O(9B)-H(9B)	0.8400
O(10B)-C(11B)	1.406(8)
C(11B)-H(26)	0.9800
C(11B)-H(28)	0.9800
C(11B)-H(27)	0.9800
N(1C)-C(6C)	1.328(7)
N(1C)-C(2C)	1.347(7)
C(2C)-C(3C)	1.375(8)
C(2C)-H(9)	0.9500
C(3C)-C(4C)	1.388(9)
C(3C)-H(10C)	0.9500
C(4C)-C(5C)	1.390(8)
C(4C)-C(7C)	1.504(9)
C(5C)-C(6C)	1.384(8)
C(5C)-H(5C)	0.9500
C(6C)-H(6C)	0.9500
C(7C)-O(9C)	1.385(9)
C(7C)-O(8C)	1.425(9)
C(7C)-H(6)	1.0000
O(8C)-H(8C)	0.8400
O(9C)-C(11C)	1.397(11)
C(11C)-H(11A)	0.9800
C(11C)-H(11B)	0.9800
C(11C)-H(11C)	0.9800
N(1D)-C(2D)	1.328(7)
N(1D)-C(6D)	1.339(7)
C(2D)-C(3D)	1.381(8)
C(2D)-H(11)	0.9500
C(3D)-C(4D)	1.385(8)
C(3D)-H(18)	0.9500
C(4D)-C(5D)	1.399(8)
C(4D)-C(7D)	1.513(8)
C(5D)-C(6D)	1.368(8)
C(5D)-H(17)	0.9500
C(6D)-H(16)	0.9500
C(7D)-O(8D)	1.392(7)
C(7D)-O(9D)	1.407(7)

C(7D)-H(7D)	1.0000
O(8D)-H(8D)	0.8400
O(9D)-C(10D)	1.406(8)
C(10D)-H(10)	0.9800
C(10D)-H(15)	0.9800
C(10D)-H(14)	0.9800
O(2W)-O(4W)	1.364(12)
N(1A)-Co(1)-N(1B)	179.38(15)
N(1A)-Co(1)-N(1C)	90.42(16)
N(1B)-Co(1)-N(1C)	89.86(16)
N(1A)-Co(1)-N(1D)	90.36(15)
N(1B)-Co(1)-N(1D)	89.35(16)
N(1C)-Co(1)-N(1D)	178.80(15)
N(1A)-Co(1)-Cl(2)	90.90(11)
N(1B)-Co(1)-Cl(2)	89.64(12)
N(1C)-Co(1)-Cl(2)	90.71(12)
N(1D)-Co(1)-Cl(2)	90.19(12)
N(1A)-Co(1)-Cl(1)	87.81(11)
N(1B)-Co(1)-Cl(1)	91.65(12)
N(1C)-Co(1)-Cl(1)	89.43(12)
N(1D)-Co(1)-Cl(1)	89.69(12)
Cl(2)-Co(1)-Cl(1)	178.70(5)
C(2A)-N(1A)-C(6A)	117.8(4)
C(2A)-N(1A)-Co(1)	120.6(3)
C(6A)-N(1A)-Co(1)	121.6(3)
N(1A)-C(2A)-C(3A)	123.2(5)
N(1A)-C(2A)-H(30)	118.4
C(3A)-C(2A)-H(30)	118.4
C(2A)-C(3A)-C(4A)	118.9(5)
C(2A)-C(3A)-H(35)	120.5
C(4A)-C(3A)-H(35)	120.5
C(3A)-C(4A)-C(5A)	118.1(5)
C(3A)-C(4A)-C(7A)	119.1(4)
C(5A)-C(4A)-C(7A)	122.8(4)
C(6A)-C(5A)-C(4A)	119.6(4)
C(6A)-C(5A)-H(5A)	120.2
C(4A)-C(5A)-H(5A)	120.2
N(1A)-C(6A)-C(5A)	122.3(4)
N(1A)-C(6A)-H(34)	118.8
C(5A)-C(6A)-H(34)	118.8

O(8A)-C(7A)-O(10A)	110.3(7)
O(8A)-C(7A)-O(9A)	95.4(6)
O(10A)-C(7A)-O(9A)	118.6(6)
O(8A)-C(7A)-C(4A)	113.7(7)
O(10A)-C(7A)-C(4A)	107.1(4)
O(9A)-C(7A)-C(4A)	111.6(5)
C(7A)-O(8A)-H(8A)	109.5
C(7A)-O(9A)-H(9A)	109.5
C(7A)-O(10A)-C(11A)	113.8(5)
O(10A)-C(11A)-H(32)	109.5
O(10A)-C(11A)-H(33)	109.5
H(32)-C(11A)-H(33)	109.5
O(10A)-C(11A)-H(31)	109.5
H(32)-C(11A)-H(31)	109.5
H(33)-C(11A)-H(31)	109.5
C(2B)-N(1B)-C(6B)	116.6(5)
C(2B)-N(1B)-Co(1)	123.4(3)
C(6B)-N(1B)-Co(1)	120.0(3)
N(1B)-C(2B)-C(3B)	123.1(5)
N(1B)-C(2B)-H(2B)	118.4
C(3B)-C(2B)-H(2B)	118.4
C(2B)-C(3B)-C(4B)	119.8(5)
C(2B)-C(3B)-H(21)	120.1
C(4B)-C(3B)-H(21)	120.1
C(3B)-C(4B)-C(5B)	118.3(5)
C(3B)-C(4B)-C(7B)	120.9(5)
C(5B)-C(4B)-C(7B)	120.8(5)
C(6B)-C(5B)-C(4B)	118.4(5)
C(6B)-C(5B)-H(29)	120.8
C(4B)-C(5B)-H(29)	120.8
N(1B)-C(6B)-C(5B)	123.8(5)
N(1B)-C(6B)-H(19)	118.1
C(5B)-C(6B)-H(19)	118.1
O(8B)-C(7B)-O(9B)	94.3(6)
O(8B)-C(7B)-O(10B)	115.9(7)
O(9B)-C(7B)-O(10B)	116.4(6)
O(8B)-C(7B)-C(4B)	112.8(7)
O(9B)-C(7B)-C(4B)	109.2(5)
O(10B)-C(7B)-C(4B)	107.8(4)
C(7B)-O(8B)-H(8B)	109.5

C(7B)-O(9B)-H(9B)	109.5
C(7B)-O(10B)-C(11B)	114.7(5)
O(10B)-C(11B)-H(26)	109.5
O(10B)-C(11B)-H(28)	109.5
H(26)-C(11B)-H(28)	109.5
O(10B)-C(11B)-H(27)	109.5
H(26)-C(11B)-H(27)	109.5
H(28)-C(11B)-H(27)	109.5
C(6C)-N(1C)-C(2C)	116.7(5)
C(6C)-N(1C)-Co(1)	120.1(4)
C(2C)-N(1C)-Co(1)	123.1(4)
N(1C)-C(2C)-C(3C)	123.5(5)
N(1C)-C(2C)-H(9)	118.2
C(3C)-C(2C)-H(9)	118.2
C(2C)-C(3C)-C(4C)	119.4(5)
C(2C)-C(3C)-H(10C)	120.3
C(4C)-C(3C)-H(10C)	120.3
C(3C)-C(4C)-C(5C)	117.4(5)
C(3C)-C(4C)-C(7C)	123.1(6)
C(5C)-C(4C)-C(7C)	119.6(6)
C(6C)-C(5C)-C(4C)	119.2(6)
C(6C)-C(5C)-H(5C)	120.4
C(4C)-C(5C)-H(5C)	120.4
N(1C)-C(6C)-C(5C)	123.7(5)
N(1C)-C(6C)-H(6C)	118.1
C(5C)-C(6C)-H(6C)	118.1
O(9C)-C(7C)-O(8C)	111.4(7)
O(9C)-C(7C)-C(4C)	105.8(5)
O(8C)-C(7C)-C(4C)	111.0(6)
O(9C)-C(7C)-H(6)	109.5
O(8C)-C(7C)-H(6)	109.5
C(4C)-C(7C)-H(6)	109.5
C(7C)-O(8C)-H(8C)	109.5
C(7C)-O(9C)-C(11C)	112.1(7)
O(9C)-C(11C)-H(11A)	109.5
O(9C)-C(11C)-H(11B)	109.5
H(11A)-C(11C)-H(11B)	109.5
O(9C)-C(11C)-H(11C)	109.5
H(11A)-C(11C)-H(11C)	109.5
H(11B)-C(11C)-H(11C)	109.5

C(2D)-N(1D)-C(6D)	117.3(5)
C(2D)-N(1D)-Co(1)	122.5(4)
C(6D)-N(1D)-Co(1)	120.2(3)
N(1D)-C(2D)-C(3D)	123.0(5)
N(1D)-C(2D)-H(11)	118.5
C(3D)-C(2D)-H(11)	118.5
C(2D)-C(3D)-C(4D)	119.7(5)
C(2D)-C(3D)-H(18)	120.2
C(4D)-C(3D)-H(18)	120.2
C(3D)-C(4D)-C(5D)	117.4(5)
C(3D)-C(4D)-C(7D)	122.4(5)
C(5D)-C(4D)-C(7D)	120.2(5)
C(6D)-C(5D)-C(4D)	118.7(5)
C(6D)-C(5D)-H(17)	120.6
C(4D)-C(5D)-H(17)	120.6
N(1D)-C(6D)-C(5D)	123.9(5)
N(1D)-C(6D)-H(16)	118.0
C(5D)-C(6D)-H(16)	118.0
O(8D)-C(7D)-O(9D)	113.8(5)
O(8D)-C(7D)-C(4D)	112.4(5)
O(9D)-C(7D)-C(4D)	105.9(4)
O(8D)-C(7D)-H(7D)	108.2
O(9D)-C(7D)-H(7D)	108.2
C(4D)-C(7D)-H(7D)	108.2
C(7D)-O(8D)-H(8D)	109.5
C(10D)-O(9D)-C(7D)	114.1(5)
O(9D)-C(10D)-H(10)	109.5
O(9D)-C(10D)-H(15)	109.5
H(10)-C(10D)-H(15)	109.5
O(9D)-C(10D)-H(14)	109.5
H(10)-C(10D)-H(14)	109.5
H(15)-C(10D)-H(14)	109.5
O(2W)-O(4W)-O(2W)#1	180.0

Symmetry transformations used to generate equivalent atoms:

#1 -x+4/3,-y+2/3,-z+2/3

Table S7. Torsion angles [°] for 4-pyridinecarboxaldehyde with $CoCl_2/CH_3OH$ (**AC35_2_0m**).

C(6A)-N(1A)-C(2A)-C(3A)	-0.9(8)
Co(1)-N(1A)-C(2A)-C(3A)	180.0(4)
N(1A)-C(2A)-C(3A)-C(4A)	0.5(8)
C(2A)-C(3A)-C(4A)-C(5A)	-1.2(7)
C(2A)-C(3A)-C(4A)-C(7A)	179.5(5)
C(3A)-C(4A)-C(5A)-C(6A)	2.3(7)
C(7A)-C(4A)-C(5A)-C(6A)	-178.4(5)
C(2A)-N(1A)-C(6A)-C(5A)	2.0(7)
Co(1)-N(1A)-C(6A)-C(5A)	-178.8(4)
C(4A)-C(5A)-C(6A)-N(1A)	-2.8(7)
C(3A)-C(4A)-C(7A)-O(8A)	62.2(8)
C(5A)-C(4A)-C(7A)-O(8A)	-117.0(8)
C(3A)-C(4A)-C(7A)-O(10A)	-59.9(6)
C(5A)-C(4A)-C(7A)-O(10A)	120.8(5)
C(3A)-C(4A)-C(7A)-O(9A)	168.8(5)
C(5A)-C(4A)-C(7A)-O(9A)	-10.5(7)
O(8A)-C(7A)-O(10A)-C(11A)	49.5(8)
O(9A)-C(7A)-O(10A)-C(11A)	-58.9(7)
C(4A)-C(7A)-O(10A)-C(11A)	173.8(5)
C(6B)-N(1B)-C(2B)-C(3B)	0.1(8)
Co(1)-N(1B)-C(2B)-C(3B)	178.6(4)
N(1B)-C(2B)-C(3B)-C(4B)	0.5(8)
C(2B)-C(3B)-C(4B)-C(5B)	-0.1(8)
C(2B)-C(3B)-C(4B)-C(7B)	-179.7(5)
C(3B)-C(4B)-C(5B)-C(6B)	-0.9(8)
C(7B)-C(4B)-C(5B)-C(6B)	178.7(5)
C(2B)-N(1B)-C(6B)-C(5B)	-1.2(8)
Co(1)-N(1B)-C(6B)-C(5B)	-179.7(4)
C(4B)-C(5B)-C(6B)-N(1B)	1.6(9)
C(3B)-C(4B)-C(7B)-O(8B)	9.5(8)
C(5B)-C(4B)-C(7B)-O(8B)	-170.1(6)
C(3B)-C(4B)-C(7B)-O(9B)	-93.9(7)
C(5B)-C(4B)-C(7B)-O(9B)	86.5(7)
C(3B)-C(4B)-C(7B)-O(10B)	138.8(5)
C(5B)-C(4B)-C(7B)-O(10B)	-40.8(7)
O(8B)-C(7B)-O(10B)-C(11B)	-49.6(9)
O(9B)-C(7B)-O(10B)-C(11B)	60.0(9)
C(4B)-C(7B)-O(10B)-C(11B)	-177.1(6)

C(6C)-N(1C)-C(2C)-C(3C)	1.3(8)
Co(1)-N(1C)-C(2C)-C(3C)	-180.0(4)
N(1C)-C(2C)-C(3C)-C(4C)	-1.5(8)
C(2C)-C(3C)-C(4C)-C(5C)	1.0(8)
C(2C)-C(3C)-C(4C)-C(7C)	-178.2(5)
C(3C)-C(4C)-C(5C)-C(6C)	-0.6(9)
C(7C)-C(4C)-C(5C)-C(6C)	178.7(6)
C(2C)-N(1C)-C(6C)-C(5C)	-0.8(9)
Co(1)-N(1C)-C(6C)-C(5C)	-179.6(5)
C(4C)-C(5C)-C(6C)-N(1C)	0.5(11)
C(3C)-C(4C)-C(7C)-O(9C)	119.2(8)
C(5C)-C(4C)-C(7C)-O(9C)	-60.0(9)
C(3C)-C(4C)-C(7C)-O(8C)	-119.8(7)
C(5C)-C(4C)-C(7C)-O(8C)	61.0(8)
O(8C)-C(7C)-O(9C)-C(11C)	68.5(14)
C(4C)-C(7C)-O(9C)-C(11C)	-170.7(12)
C(6D)-N(1D)-C(2D)-C(3D)	-2.2(8)
Co(1)-N(1D)-C(2D)-C(3D)	175.5(4)
N(1D)-C(2D)-C(3D)-C(4D)	1.4(9)
C(2D)-C(3D)-C(4D)-C(5D)	0.5(8)
C(2D)-C(3D)-C(4D)-C(7D)	-178.8(5)
C(3D)-C(4D)-C(5D)-C(6D)	-1.4(7)
C(7D)-C(4D)-C(5D)-C(6D)	177.8(5)
C(2D)-N(1D)-C(6D)-C(5D)	1.1(8)
Co(1)-N(1D)-C(6D)-C(5D)	-176.6(4)
C(4D)-C(5D)-C(6D)-N(1D)	0.7(8)
C(3D)-C(4D)-C(7D)-O(8D)	16.8(7)
C(5D)-C(4D)-C(7D)-O(8D)	-162.4(5)
C(3D)-C(4D)-C(7D)-O(9D)	141.7(5)
C(5D)-C(4D)-C(7D)-O(9D)	-37.5(7)
O(8D)-C(7D)-O(9D)-C(10D)	-66.3(8)
C(4D)-C(7D)-O(9D)-C(10D)	169.6(6)

Symmetry transformations used to generate equivalent atoms:

#1 -x+4/3,-y+2/3,-z+2/3

Table S8. Hydrogen bonds for 4-pyridinecarboxaldehyde with $CoCl_2/CH_3OH$ (**AC35_2_0m).** [pm and °].

D-H...A	d(D-H)	d(H...A)	d(D...A)	<(DHA)
C(6A)-H(34)...Cl(2)	0.95	2.97	3.415(5)	110.4
C(6A)-H(34)...O(10A)#2	0.95	2.59	3.417(6)	145.7
O(8A)-H(8A)...Cl(2)#2	0.84	2.13	2.912(13)	155.1
O(9A)-H(9A)...O(1W)	0.84	2.03	2.777(7)	147.7
C(11A)-H(33)...Cl(2)#3	0.98	2.89	3.789(8)	152.5
C(2B)-H(2B)...Cl(2)	0.95	2.80	3.315(6)	115.2
C(6B)-H(19)...Cl(1)	0.95	2.80	3.358(6)	118.6
C(11B)-H(28)...Cl(1)#4	0.98	2.99	3.830(9)	144.8
C(2C)-H(9)...Cl(2)	0.95	2.89	3.385(6)	113.7
C(6C)-H(6C)...Cl(1)	0.95	2.81	3.324(6)	114.8
C(6C)-H(6C)...O(10B)#5	0.95	2.56	3.261(7)	130.8
O(8C)-H(8C)...O(8B)#6	0.84	1.73	2.552(10)	165.5
C(11C)-H(11B)...Cl(2)#5	0.98	2.83	3.730(11)	152.9
C(2D)-H(11)...Cl(2)	0.95	2.89	3.356(6)	111.8
C(6D)-H(16)...Cl(1)	0.95	2.77	3.328(5)	118.1
O(8D)-H(8D)...O(8A)#7	0.84	1.71	2.528(11)	164.7

Symmetry transformations used to generate equivalent atoms:

#1 -x+4/3,-y+2/3,-z+2/3 #2 y-1/3,-x+y+1/3,-z+1/3

#3 x-y+2/3,x+1/3,-z+1/3 #4 x-y+1/3,x-1/3,-z+2/3

#5 y+1/3,-x+y+2/3,-z+2/3 #6 -y+4/3,x-y+2/3,z-1/3

#7 -y+2/3,x-y+1/3,z+1/3

***Co(II)-3-pyridinecarboxaldehyde (CIF FILE: C5_0m, CCDC 2047189)**

Crystal system is monoclinic, $P2_1/c$, unit cell a: 7.7191(15), b: 36.122(7), c: 8.4932(17) Å β:92.41°. 18330 reflections were collected, 4016 independents, Rint: 0.15. The structure was solved using program SHELXS-97[1] and refined using the full-matrix LS procedure with SHELXL-2014/7.[2] Anisotropic displacement parameters were employed for non-hydrogen atoms. All H atoms were located at the expected positions and they were refined using a riding model. H atoms for water molecule were located in the Fourier difference density map. LS weights of the form $w=1/[\sigma^2(F_o^2) + (0.0313\,P)^2 + 6.1654\,P]$ where $P = (F_o^2 + 2F_c^2)/3$, were employed. $R[F^2>2\sigma(F^2)] = 0.065$, $wR(F^2) = 0.115$.

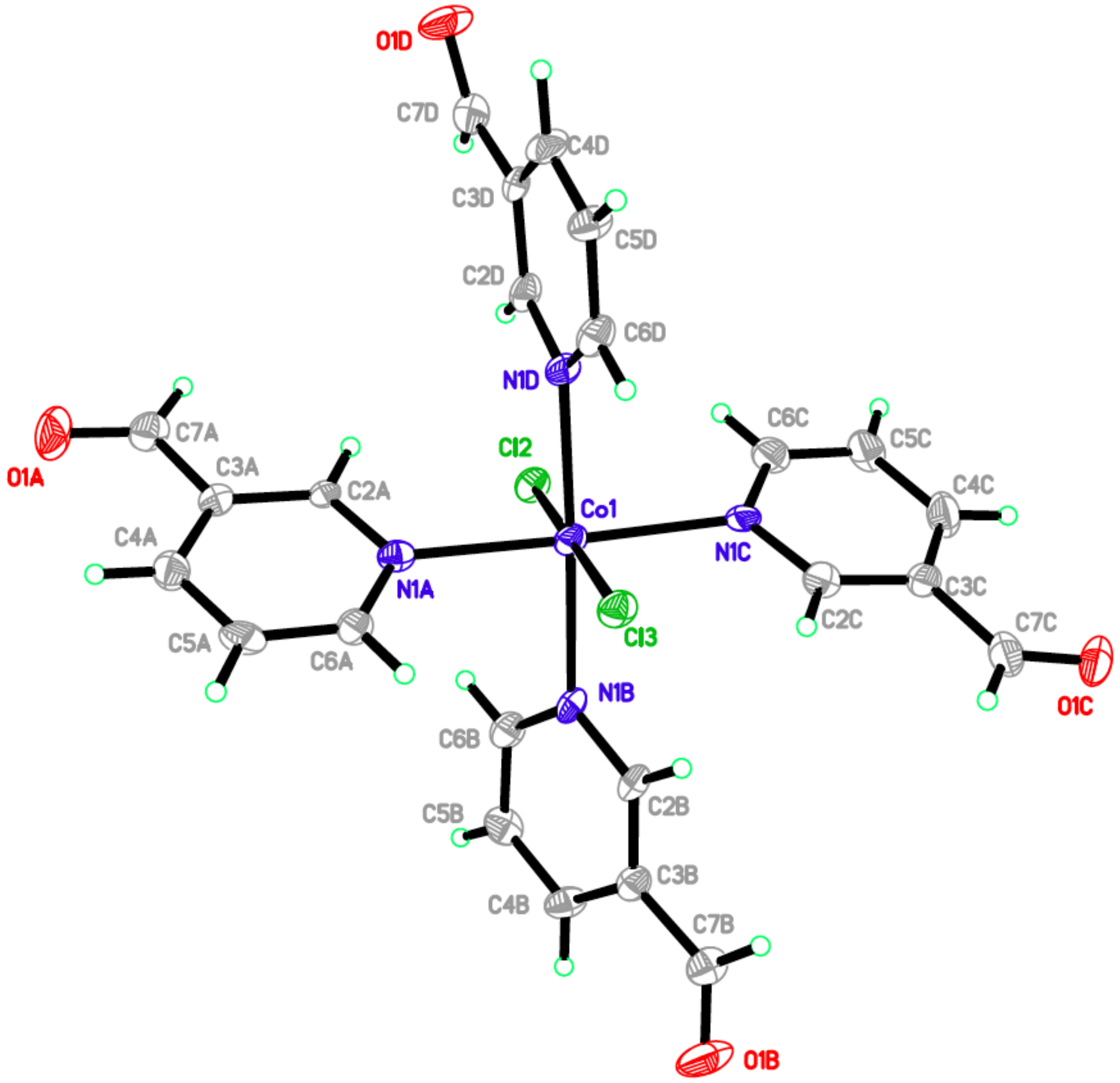

Figure S5. Crystal structure and numbering scheme for the cobalt complex for the 3-pyridinecarboxaldehyde with $CoCl_2/CH_3OH$. The displacement ellipsoids for the non-H atoms in the figure were drawn at the 50% probability level.

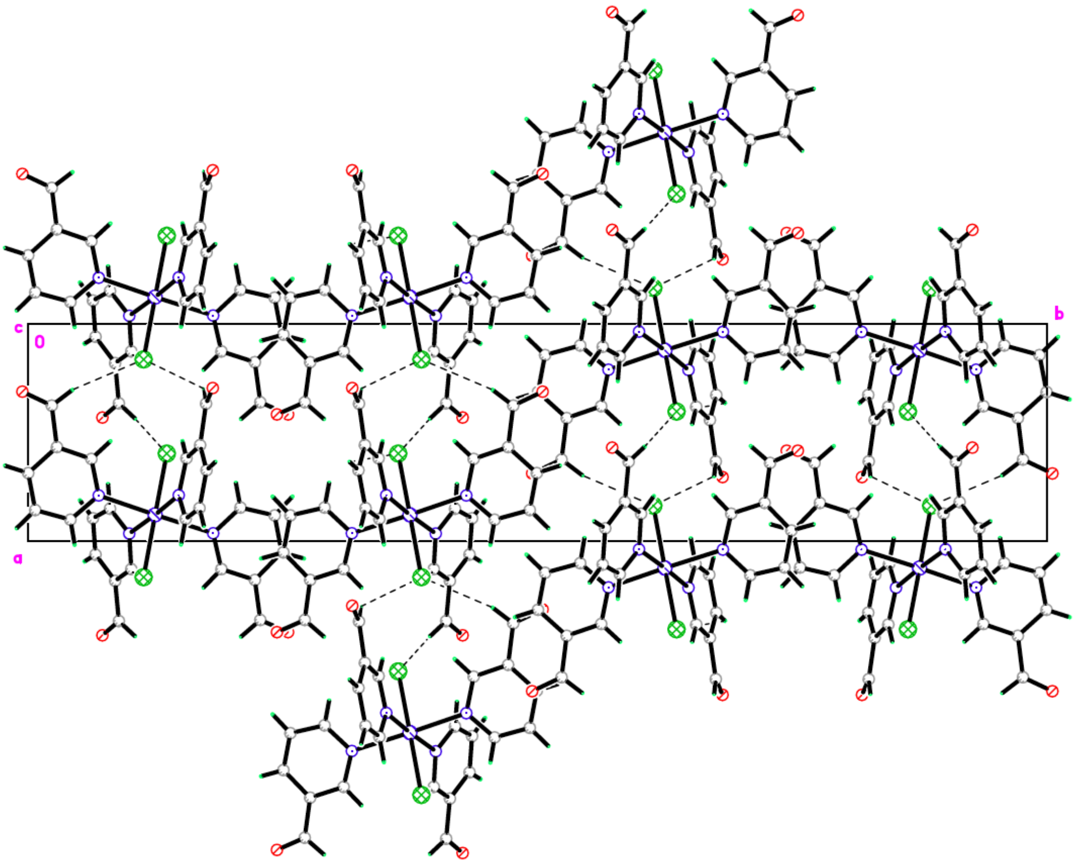

Figure S6. Crystal packing structure for the cobalt complex for the 3-pyridinecarboxaldehyde with $CoCl_2$/CH_3OH. The displacement ellipsoids for the non-H atoms in the figure were drawn at the 50% probability level.

Table S9. Crystal data and structure refinement for 3-pyridinecarboxaldehyde with $CoCl_2/CH_3OH$ (**C5_0m)**

Identification code	**C5_0m (CCDC 2047189)**	
Empirical formula	C24 H20 Cl2 Co N4 O4	
Formula weight	558.27	
Temperature	293(2) K	
Wavelength	0.71073 Å	
Crystal system	Monoclinic	
Space group	P 21/c	
Unit cell dimensions	a = 7.7191(15) Å	α= 90°.
	b = 36.122(7) Å	β= 92.41(3)°.
	c = 8.4932(17) Å	γ= 90°.
Volume	2366.0(8) $Å^3$	
Z	4	
Density (calculated)	1.567 Mg/m^3	
Absorption coefficient	0.991 mm^{-1}	
F(000)	1140	
Crystal size	0.25 x 0.2 x 0.15 mm^3	
Theta range for data collection	2.466 to 24.724°.	
Index ranges	-9<=h<=9, -42<=k<=42, -9<=l<=9	
Reflections collected	18330	
Independent reflections	4016 [R(int) = 0.1501]	
Completeness to theta = 24.724°	99.8 %	
Absorption correction	Semi-empirical from equivalents	
Refinement method	Full-matrix least-squares on F^2	
Data / restraints / parameters	4016 / 0 / 316	
Goodness-of-fit on F^2	1.106	
Final R indices [I>2sigma(I)]	R1 = 0.0650, wR2 = 0.1151	
R indices (all data)	R1 = 0.1276, wR2 = 0.1303	
Extinction coefficient	n/a	
Largest diff. peak and hole	0.427 and -0.650 e.$Å^{-3}$	

Table S10. Bond lengths [Å] and angles [°] for 3-pyridinecarboxaldehyde with $CoCl_2/CH_3OH$ (**C5_0m)**

Co(1)-N(1A)	2.213(5)
Co(1)-N(1D)	2.222(5)
Co(1)-N(1B)	2.234(5)
Co(1)-N(1C)	2.245(5)
Co(1)-Cl(3)	2.4077(18)
Co(1)-Cl(2)	2.4185(19)
O(1A)-C(7A)	1.205(7)
N(1A)-C(6A)	1.331(7)
N(1A)-C(2A)	1.357(7)
C(2A)-C(3A)	1.372(8)
C(3A)-C(4A)	1.387(8)
C(3A)-C(7A)	1.504(9)
C(4A)-C(5A)	1.374(8)
C(5A)-C(6A)	1.380(8)
O(1B)-C(7B)	1.202(7)
N(1B)-C(6B)	1.341(8)
N(1B)-C(2B)	1.341(7)
C(2B)-C(3B)	1.384(8)
C(3B)-C(4B)	1.387(9)
C(3B)-C(7B)	1.487(8)
C(4B)-C(5B)	1.367(9)
C(5B)-C(6B)	1.385(9)
O(1C)-C(7C)	1.202(8)
N(1C)-C(6C)	1.349(8)
N(1C)-C(2C)	1.350(7)
C(2C)-C(3C)	1.380(8)
C(3C)-C(4C)	1.387(9)
C(3C)-C(7C)	1.478(9)
C(4C)-C(5C)	1.387(9)
C(5C)-C(6C)	1.373(9)
O(1D)-C(7D)	1.201(8)
N(1D)-C(6D)	1.329(8)
N(1D)-C(2D)	1.355(7)
C(2D)-C(3D)	1.387(8)
C(3D)-C(4D)	1.369(9)
C(3D)-C(7D)	1.489(9)
C(4D)-C(5D)	1.386(9)
C(5D)-C(6D)	1.394(9)

N(1A)-Co(1)-N(1D)	89.07(18)
N(1A)-Co(1)-N(1B)	88.72(18)
N(1D)-Co(1)-N(1B)	177.8(2)
N(1A)-Co(1)-N(1C)	178.23(19)
N(1D)-Co(1)-N(1C)	89.28(18)
N(1B)-Co(1)-N(1C)	92.92(18)
N(1A)-Co(1)-Cl(3)	91.40(13)
N(1D)-Co(1)-Cl(3)	92.12(14)
N(1B)-Co(1)-Cl(3)	88.24(14)
N(1C)-Co(1)-Cl(3)	89.32(14)
N(1A)-Co(1)-Cl(2)	89.81(13)
N(1D)-Co(1)-Cl(2)	90.53(14)
N(1B)-Co(1)-Cl(2)	89.16(14)
N(1C)-Co(1)-Cl(2)	89.54(14)
Cl(3)-Co(1)-Cl(2)	177.11(7)
C(6A)-N(1A)-C(2A)	116.1(5)
C(6A)-N(1A)-Co(1)	121.3(4)
C(2A)-N(1A)-Co(1)	122.5(4)
N(1A)-C(2A)-C(3A)	122.7(6)
C(2A)-C(3A)-C(4A)	120.1(6)
C(2A)-C(3A)-C(7A)	117.8(6)
C(4A)-C(3A)-C(7A)	122.2(6)
C(5A)-C(4A)-C(3A)	117.7(6)
C(4A)-C(5A)-C(6A)	118.8(6)
N(1A)-C(6A)-C(5A)	124.6(6)
O(1A)-C(7A)-C(3A)	123.8(6)
C(6B)-N(1B)-C(2B)	116.9(6)
C(6B)-N(1B)-Co(1)	119.8(4)
C(2B)-N(1B)-Co(1)	123.2(4)
N(1B)-C(2B)-C(3B)	123.3(6)
C(2B)-C(3B)-C(4B)	118.6(6)
C(2B)-C(3B)-C(7B)	118.6(6)
C(4B)-C(3B)-C(7B)	122.7(6)
C(5B)-C(4B)-C(3B)	118.7(6)
C(4B)-C(5B)-C(6B)	119.2(6)
N(1B)-C(6B)-C(5B)	123.3(6)
O(1B)-C(7B)-C(3B)	124.2(6)
C(6C)-N(1C)-C(2C)	115.7(5)
C(6C)-N(1C)-Co(1)	120.7(4)

C(2C)-N(1C)-Co(1)	122.9(4)
N(1C)-C(2C)-C(3C)	123.9(6)
C(2C)-C(3C)-C(4C)	118.4(6)
C(2C)-C(3C)-C(7C)	118.9(6)
C(4C)-C(3C)-C(7C)	122.6(6)
C(5C)-C(4C)-C(3C)	119.2(6)
C(6C)-C(5C)-C(4C)	117.9(6)
N(1C)-C(6C)-C(5C)	124.8(6)
O(1C)-C(7C)-C(3C)	124.2(7)
C(6D)-N(1D)-C(2D)	116.3(5)
C(6D)-N(1D)-Co(1)	121.7(4)
C(2D)-N(1D)-Co(1)	122.1(4)
N(1D)-C(2D)-C(3D)	123.3(6)
C(4D)-C(3D)-C(2D)	119.3(6)
C(4D)-C(3D)-C(7D)	123.1(6)
C(2D)-C(3D)-C(7D)	117.6(6)
C(3D)-C(4D)-C(5D)	118.7(6)
C(4D)-C(5D)-C(6D)	118.3(6)
N(1D)-C(6D)-C(5D)	124.2(6)
O(1D)-C(7D)-C(3D)	124.3(7)

Symmetry transformations used to generate equivalent atoms:

Table S11. Torsion angles [°] for 3-pyridinecarboxaldehyde with $CoCl_2/CH_3OH$ (**C5_0m)**

Table 3. Torsion angles [°] for c5_0m.

C(6A)-N(1A)-C(2A)-C(3A)	-0.3(9)
Co(1)-N(1A)-C(2A)-C(3A)	-176.9(5)
N(1A)-C(2A)-C(3A)-C(4A)	2.0(10)
N(1A)-C(2A)-C(3A)-C(7A)	-177.9(6)
C(2A)-C(3A)-C(4A)-C(5A)	-2.5(9)
C(7A)-C(3A)-C(4A)-C(5A)	177.3(6)
C(3A)-C(4A)-C(5A)-C(6A)	1.5(9)
C(2A)-N(1A)-C(6A)-C(5A)	-0.8(9)
Co(1)-N(1A)-C(6A)-C(5A)	175.9(5)
C(4A)-C(5A)-C(6A)-N(1A)	0.2(10)
C(2A)-C(3A)-C(7A)-O(1A)	176.3(6)
C(4A)-C(3A)-C(7A)-O(1A)	-3.5(10)

C(6B)-N(1B)-C(2B)-C(3B)	1.0(9)
Co(1)-N(1B)-C(2B)-C(3B)	179.7(4)
N(1B)-C(2B)-C(3B)-C(4B)	-0.7(9)
N(1B)-C(2B)-C(3B)-C(7B)	-179.5(5)
C(2B)-C(3B)-C(4B)-C(5B)	0.1(9)
C(7B)-C(3B)-C(4B)-C(5B)	178.8(6)
C(3B)-C(4B)-C(5B)-C(6B)	0.2(9)
C(2B)-N(1B)-C(6B)-C(5B)	-0.7(9)
Co(1)-N(1B)-C(6B)-C(5B)	-179.4(5)
C(4B)-C(5B)-C(6B)-N(1B)	0.1(10)
C(2B)-C(3B)-C(7B)-O(1B)	170.8(6)
C(4B)-C(3B)-C(7B)-O(1B)	-7.9(10)
C(6C)-N(1C)-C(2C)-C(3C)	-1.8(9)
Co(1)-N(1C)-C(2C)-C(3C)	168.7(5)
N(1C)-C(2C)-C(3C)-C(4C)	-0.5(10)
N(1C)-C(2C)-C(3C)-C(7C)	-178.5(6)
C(2C)-C(3C)-C(4C)-C(5C)	2.8(10)
C(7C)-C(3C)-C(4C)-C(5C)	-179.3(6)
C(3C)-C(4C)-C(5C)-C(6C)	-2.7(10)
C(2C)-N(1C)-C(6C)-C(5C)	1.9(10)
Co(1)-N(1C)-C(6C)-C(5C)	-168.8(5)
C(4C)-C(5C)-C(6C)-N(1C)	0.3(10)
C(2C)-C(3C)-C(7C)-O(1C)	178.8(7)
C(4C)-C(3C)-C(7C)-O(1C)	0.9(11)
C(6D)-N(1D)-C(2D)-C(3D)	-1.3(9)
Co(1)-N(1D)-C(2D)-C(3D)	178.0(4)
N(1D)-C(2D)-C(3D)-C(4D)	0.4(9)
N(1D)-C(2D)-C(3D)-C(7D)	-177.8(5)
C(2D)-C(3D)-C(4D)-C(5D)	0.7(9)
C(7D)-C(3D)-C(4D)-C(5D)	178.8(6)
C(3D)-C(4D)-C(5D)-C(6D)	-0.9(9)
C(2D)-N(1D)-C(6D)-C(5D)	1.1(9)
Co(1)-N(1D)-C(6D)-C(5D)	-178.2(5)
C(4D)-C(5D)-C(6D)-N(1D)	0.0(10)
C(4D)-C(3D)-C(7D)-O(1D)	-8.6(10)
C(2D)-C(3D)-C(7D)-O(1D)	169.6(6)

Symmetry transformations used to generate equivalent atoms:

Table S12. Hydrogen bonds for 3-pyridinecarboxaldehyde with $CoCl_2/CH_3OH$ (**C5_0m)** [Å and °].

D-H...A	d(D-H)	d(H...A)	d(D...A)	<(DHA)
C(2A)-H(2A)...Cl(2)	0.93	2.75	3.316(6)	119.7
C(6A)-H(6A)...Cl(3)	0.93	2.71	3.297(6)	122.0
C(7A)-H(7A)...Cl(3)#1	0.93	2.77	3.677(7)	165.9
C(2B)-H(2B)...Cl(3)	0.93	2.76	3.288(7)	117.3
C(6B)-H(6B)...Cl(2)	0.93	2.73	3.277(6)	118.7
C(7B)-H(7B)...Cl(2)#2	0.93	2.73	3.621(7)	159.9
C(2C)-H(2C)...Cl(3)	0.93	2.72	3.325(7)	123.2
C(6C)-H(6C)...Cl(2)	0.93	2.68	3.240(7)	119.1
C(7C)-H(7C)...Cl(2)#2	0.93	2.93	3.830(7)	163.5
C(2D)-H(2D)...Cl(2)	0.93	2.57	3.239(7)	128.8
C(6D)-H(6D)...Cl(3)	0.93	2.61	3.277(7)	129.5
C(7D)-H(7D)...Cl(3)#1	0.93	2.86	3.577(7)	134.8

Symmetry transformations used to generate equivalent atoms:

#1 x+1,y,z   #2 x-1,y,z

Solution-state NMR studies:

Solution-state NMR experiments were performed in order to understand the complex chemical functionalization observed in the X-ray structure of these systems. NMR spectra for 4-, 3- and 2-pyridinecarboxaldehyde were acquired in CD_3OD to match the solvent used during complex preparation. In each case, the presence of *gem*-diol ($\delta(^1H)$ = 5.50−5.70 ppm) and aldehyde forms ($\delta(^1H)$ = 10.00−10.20 ppm) was observed. Integration of the signals provided their relative proportions. In a previous work, we reported that the addition of water molecules to the aldehyde group in pyridinecarboxaldehyde isomers is determined by the position of the group in the aromatic ring.[3] The most reactive compound for the addition of water is the 4-pyridinecarboxaldehyde, followed by 2- and 3-pyridinecarboxaldehyde, with the fraction of *gem*-diol forms being 50, 40 and 10%, respectively, as determined by ^1H solution-state NMR in D_2O. This phenomenon is due to the π-deficient character of the pyridine ring, with positions 2, 4 and 6 being the most electron-deficient, which explains the low degree of hydration of 3-pyridinecarboxaldehyde. Interestingly, the *gem*-diol content reached 95% for 4-pyridinecarboxaldehyde in CD_3OD. However, the NMR spectrum in $CDCl_3$ only showed the aldehyde form (Figure S8), indicating that the residual water molecules present in the deuterated methanol (water content ≤ 250 ppm) were added to the aldehyde group with higher reactivity than in D_2O. Corresponding results were observed for the 3-pyridinecarboxaldehyde, where the *gem*-diol content was 75%.

A) 4-pyridinecarboxaldehyde:

***4-pyridinecarboxaldehyde in $CDCl_3$: aldehyde (100%)**

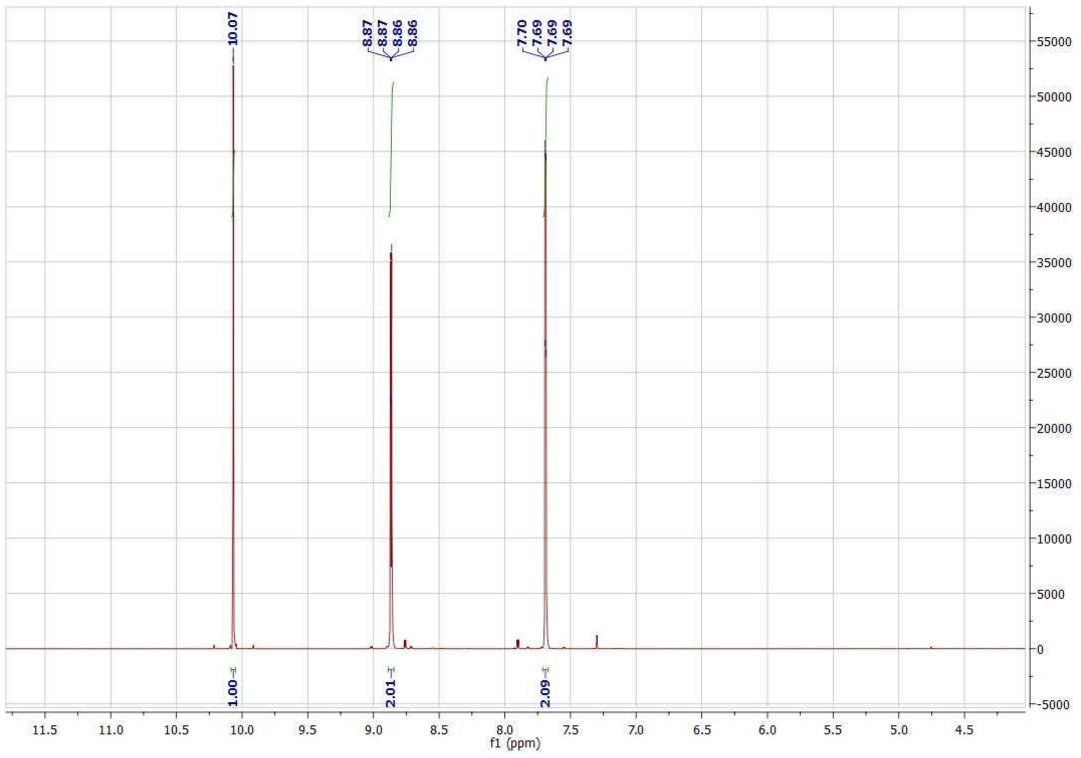

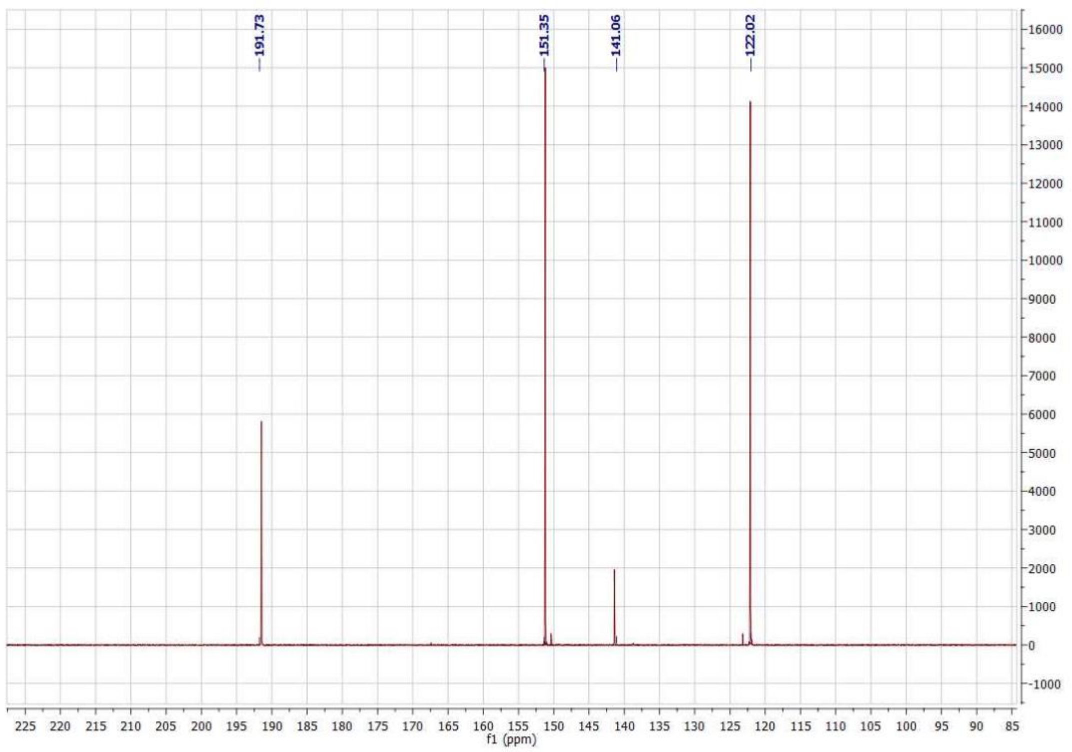

Figure S7. NMR spectra for the 4-pyridinecarboxaldehyde in $CDCl_3$. Aldehyde form (100%): ^{1}H NMR (600 MHz, CD_3Cl) δ (ppm) 10.06 (s, 1H, -C**H**O), 8.86 (d, J = 6.0 Hz, 2H, $H_{2,6}$), 7.68 (m, 2H, $H_{3,5}$). ^{13}C NMR (151 MHz, CD_3Cl) δ (ppm) 191.55 (-**C**HO), 151.30 ($C_{2,6}$), 141.52 (C_4, CHO), 122.22 ($C_{3,5}$).

***4-pyridinecarboxaldehyde in CD_3OD: aldehyde (4.70%), *gem*-diol (95.30%)**

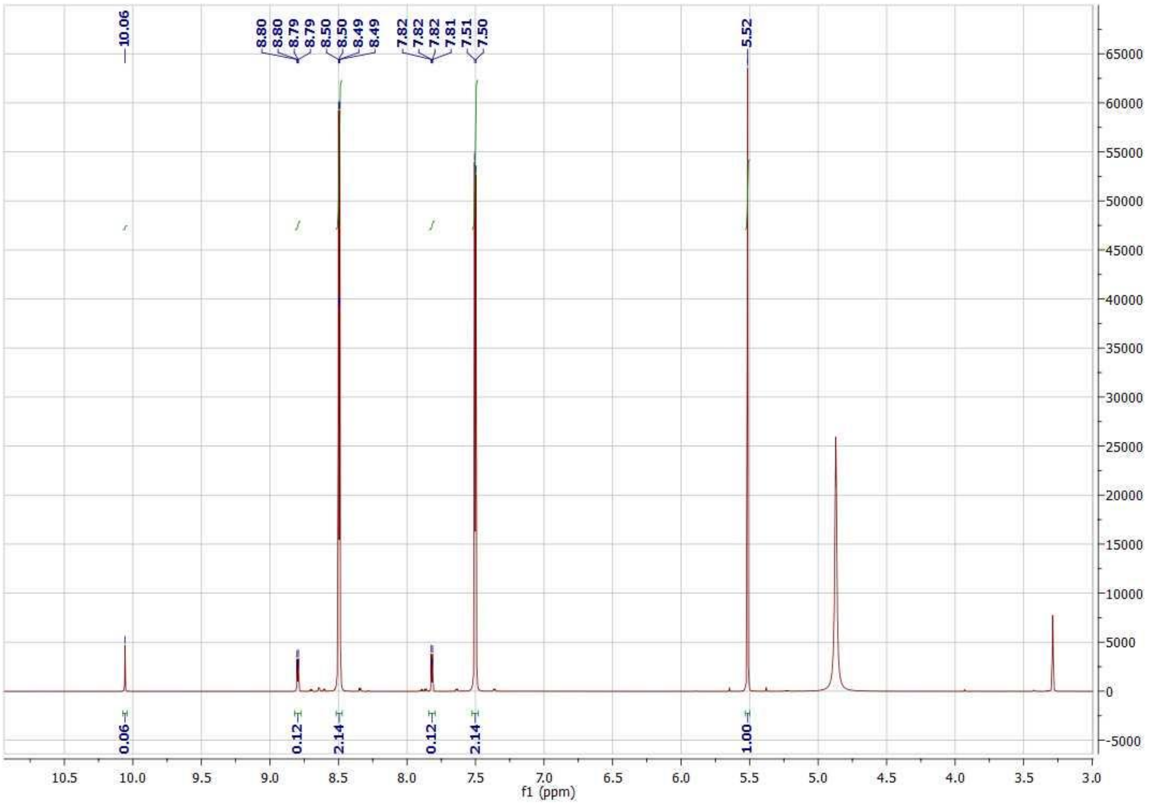

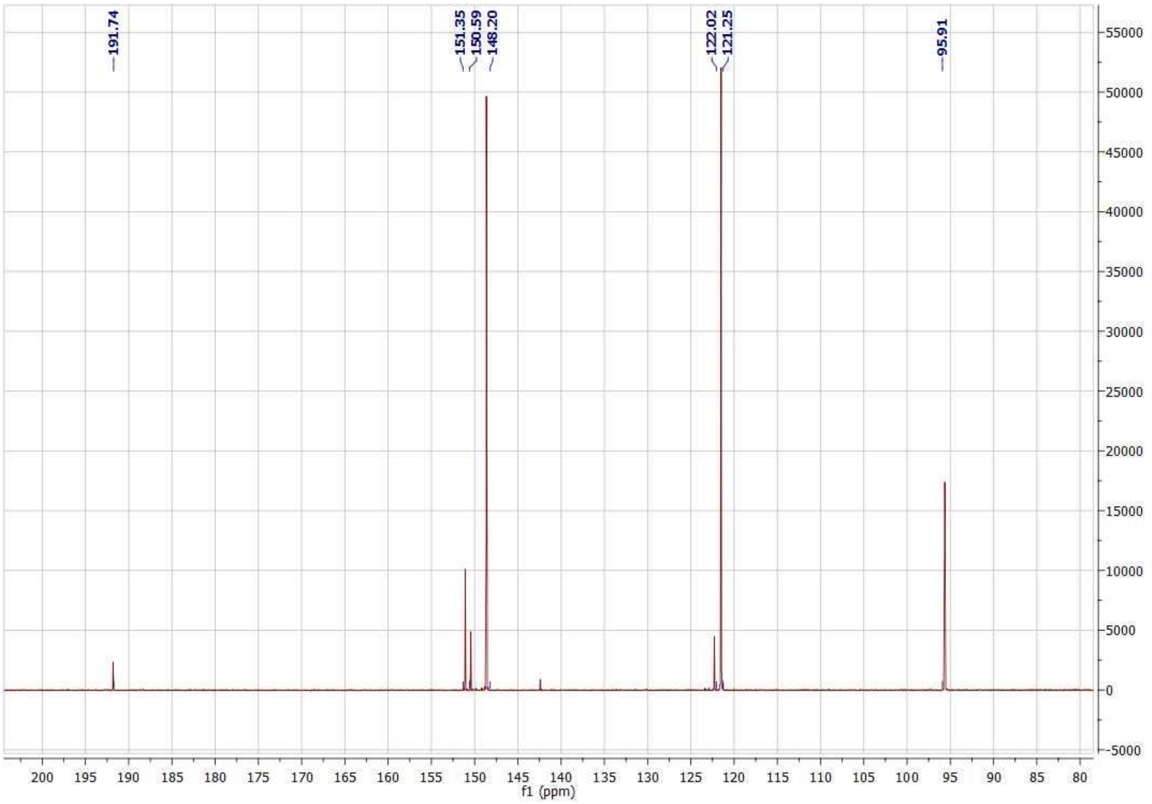

Figure S8. NMR spectra for the 4-pyridinecarboxaldehyde in CD_3OD. ^{1}H NMR (600 MHz, CD_3OD) **Aldehyde form (4.70%):** δ (ppm) 10.08 (s, 1H, (-C**H**O)), 8.82 (d, J = 6.0 Hz, 2H, $H_{2,6}$), 7.84 (d, J = 6.0 Hz, 2H, $H_{3,5}$). ***gem*-diol form (95.30%):** 8.52 (d, J = 6.2 Hz, 2H, $H_{2,6}$), 7.52 (d, J = 6.1 Hz, 2H, $H_{3,5}$), 5.54 (s, 1H, (-C**H**$(OH)_2$). ^{13}C NMR (151 MHz, CD_3OD) **Aldehyde form**

(4.70%): δ (ppm) 193.15 (-**C**HO), 151.79 ($C_{2,6}$), 143.77 (C_4), 123.64 ($C_{3,5}$), ***gem*-diol form (95.30%):** 152.44 (C_4), 150.00 ($C_{2,6}$), 122.85 ($C_{3,5}$), 97.00 (-**C**H$(OH)_2$).

***Solution-state NMR experiments with 4-pyridindecarboxaldehyde and $CuCl_2/CD_3OD$:**

Addition of copper ions to the deuterated methanolic solution of both 4- and 3-pyridinecarboxaldehyde produced a broadening of the NMR signals due to the relaxation effect of paramagnetic copper ions (Figure S9). In particular, the resonance of the *gem*-diol (RC**H**$(OH)_2$) (δ^1H: 5.54 and 5.61 ppm for the 4- and 3-pyridinecarboxaldehyde, respectively), shifted to low-frequency values as the copper concentration increased. These peaks then fuse with that of water to produce a peak at 4.88 ppm. However, the chemical shift of the aldehyde proton (RC**H**O) remained unchanged, even at the highest copper concentration (δ^1H: 10.08 and 10.11 ppm for the 4- and 3-pyridinecarboxaldehyde, respectively). The chemical shifts of the pyridine ring protons were highly affected by the addition of copper ions at any concentration and in both systems. Moreover, ^{13}C NMR spectra showed that the chemical shifts of pyridine carbon atoms were highly affected and that the peaks totally disappeared at high copper concentrations (Figure S10). The same spectral changes were observed for cobalt complexes (Figures S21 and S22).

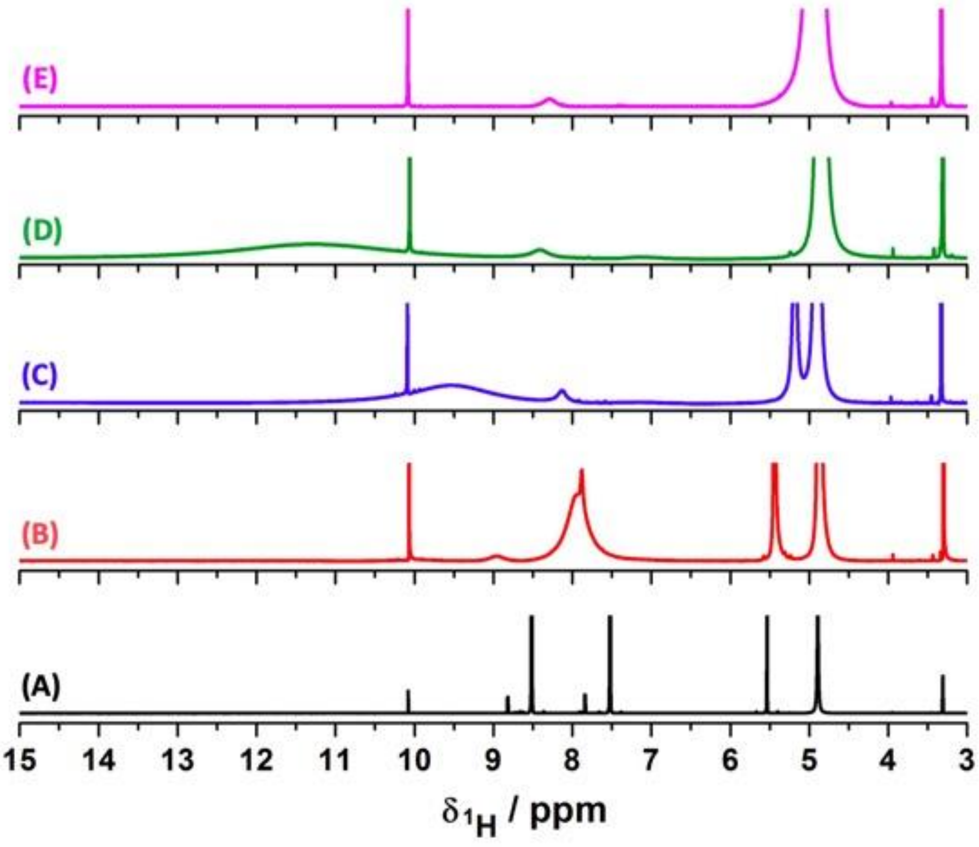

Figure S9. ^{1}H solution-state NMR for 4-pyridinecarboxaldehyde (0.28 mmol) in CD_3OD and different $CuCl_2$ additions: 0 (A), 1.21 (B), 4.86 (C), 7.29 (D) and 9.72 μmoles (E).

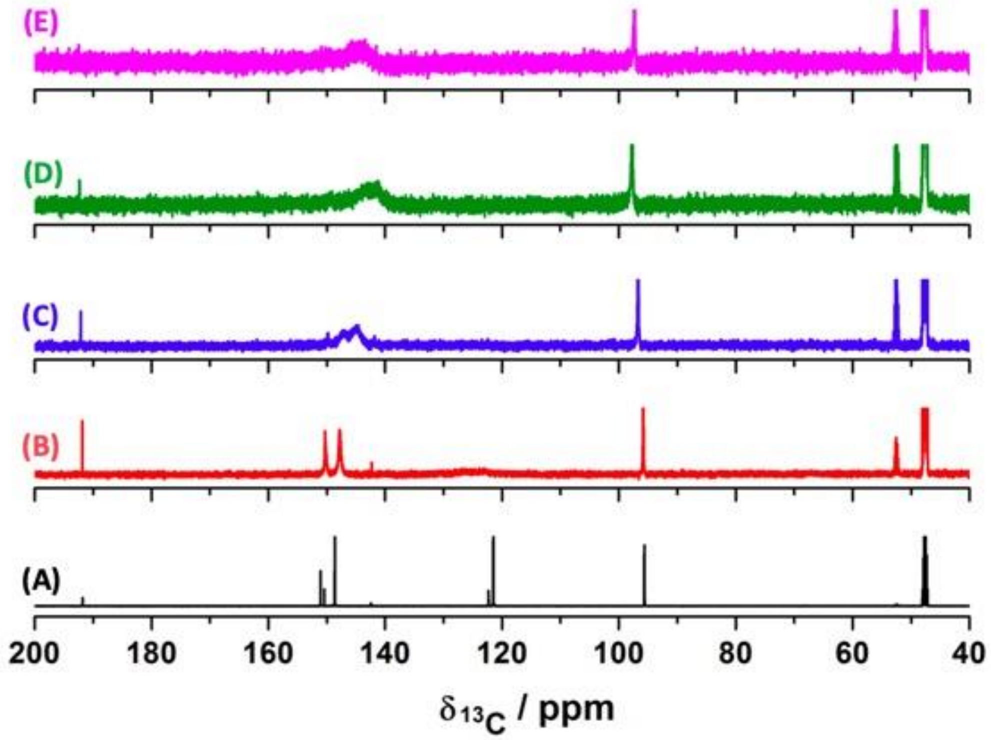

Figure S10. ^{13}C solution-state NMR spectra for 4-pyridinecarboxaldehyde (0.28 mmol) in CD_3OD and different $CuCl_2$ additions: 0 (A), 1.21 (B), 4.86 (C), 7.29 (D) and 9.72 μmol (E).

***Solution-state NMR experiments for the solid complex obtained for the 4-pyridindecarboxaldehyde and $CuCl_2/CH_3OH$ dissolved in different solvents:**

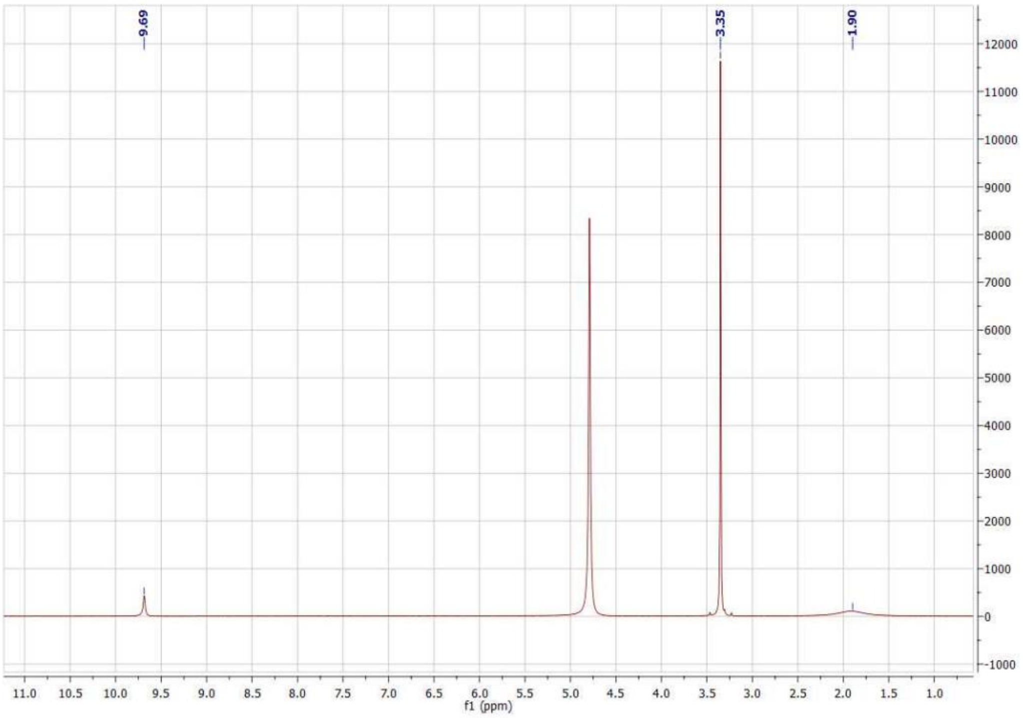

Figure S11. ^{1}H-NMR spectrum in D_2O.

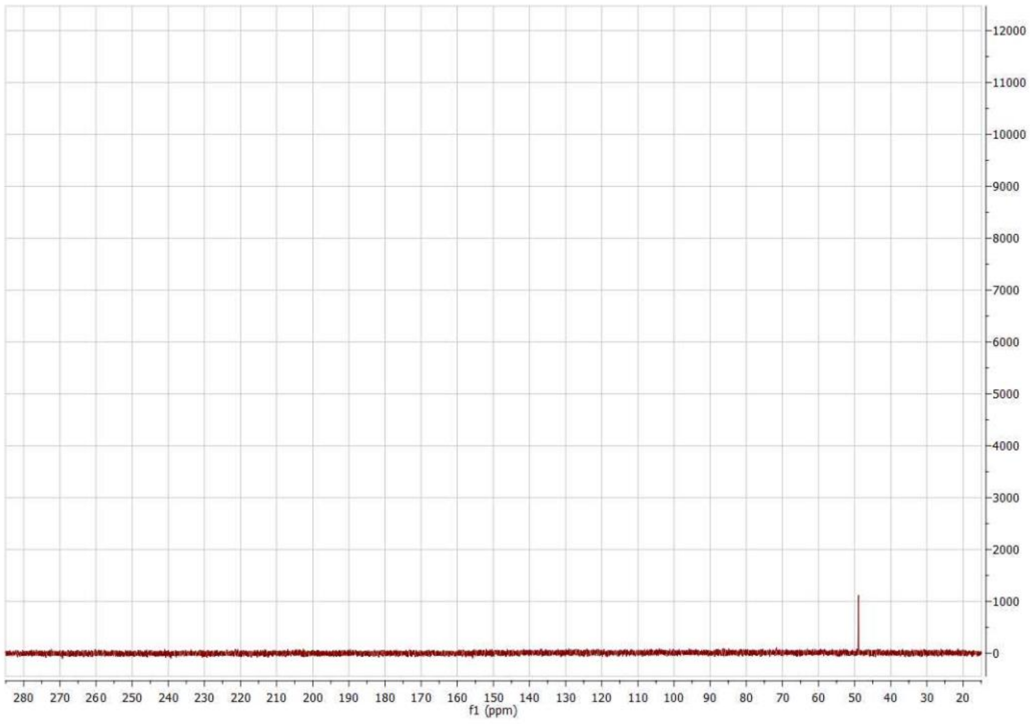

Figure S12. ^{13}C-NMR spectrum in D_2O.

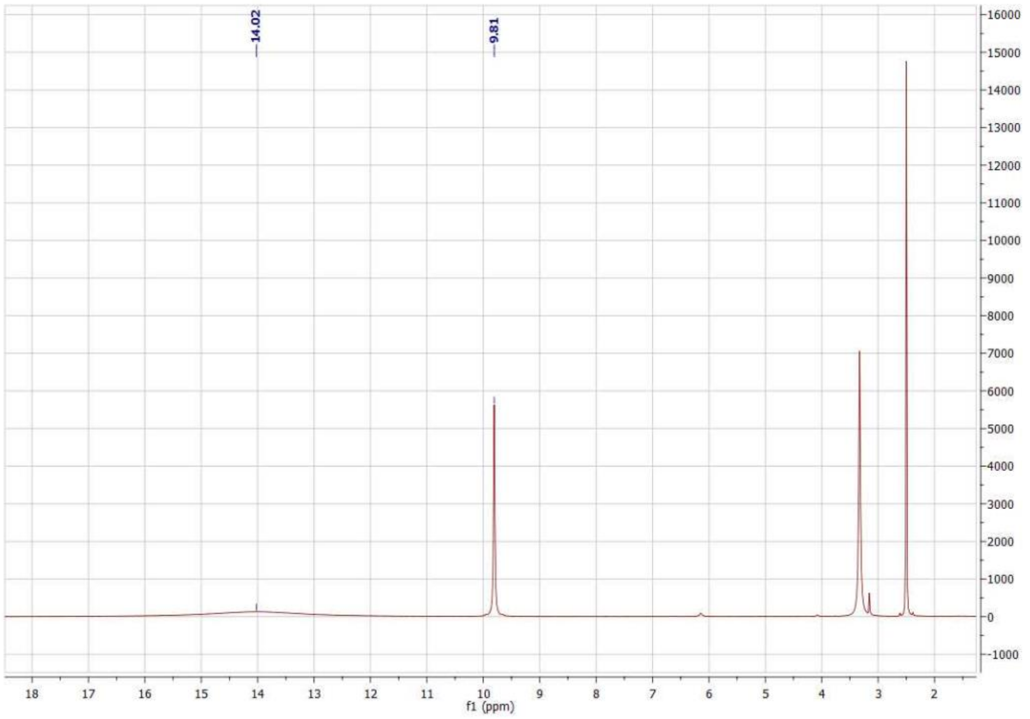

Figure S13. ^{1}H-NMR spectrum in DMSO-d_6.

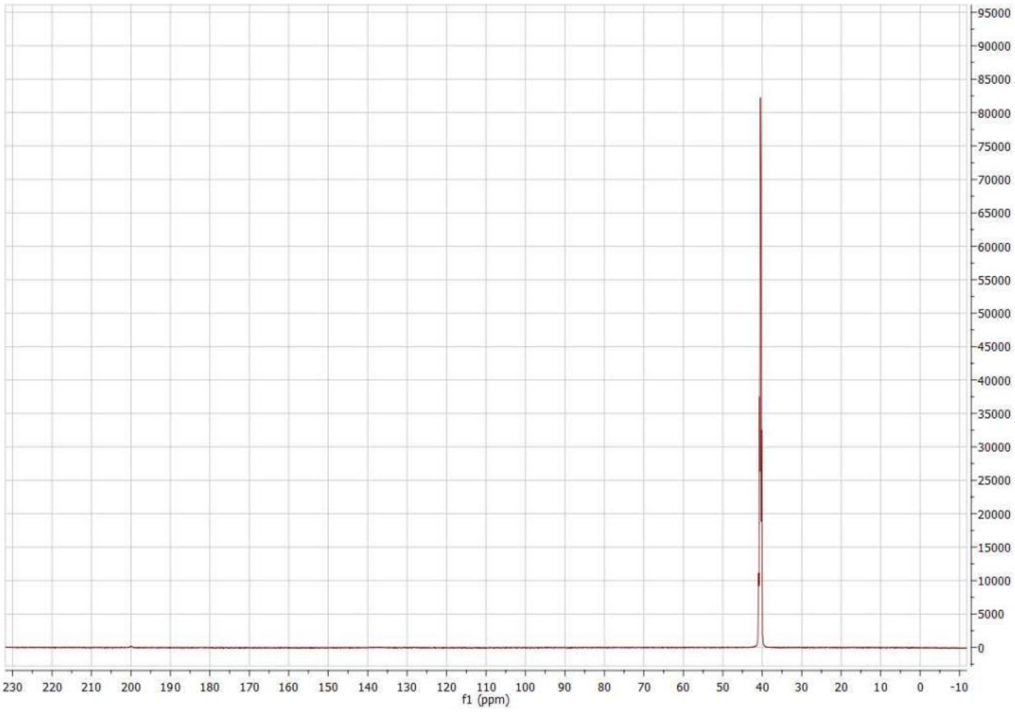

Figure S14. ^{13}C-NMR spectrum in DMSO-d_6.

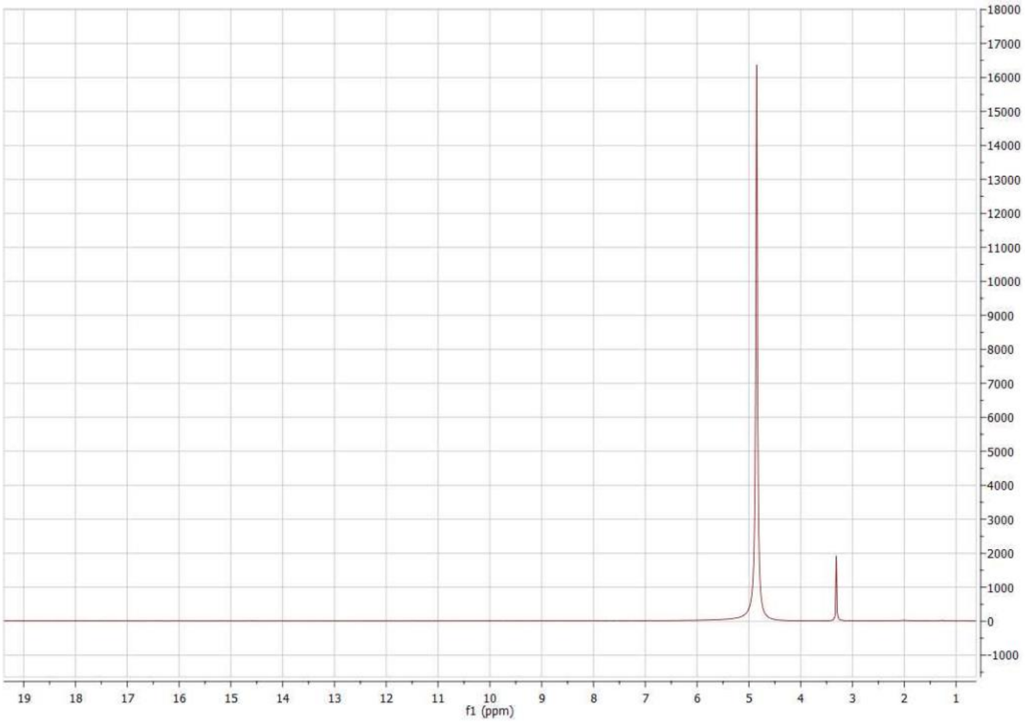

Figure S15. ^{1}H-NMR spectrum in CD_3OD.

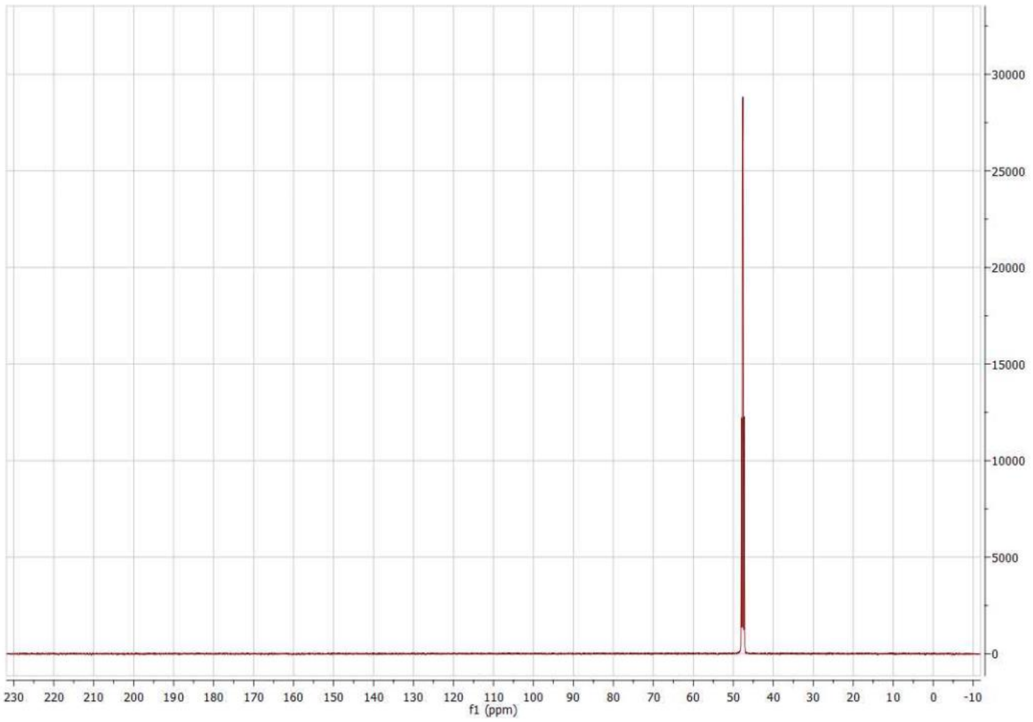

Figure S16. ^{13}C-NMR spectrum in CD_3OD.

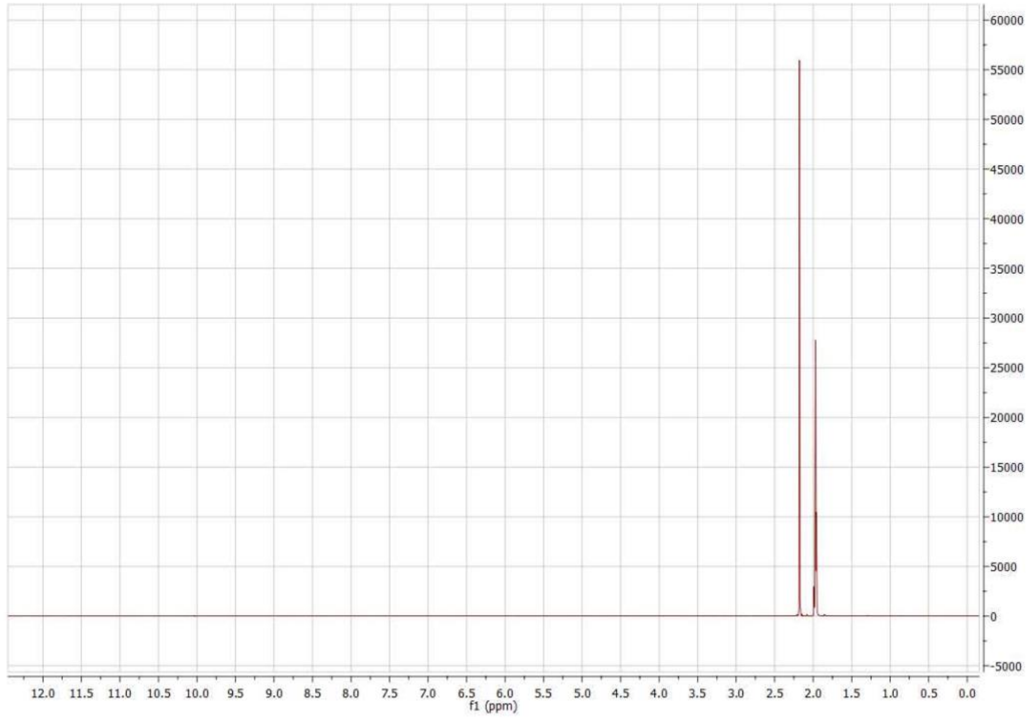

Figure S17. ^{1}H-NMR spectrum in CD_3CN.

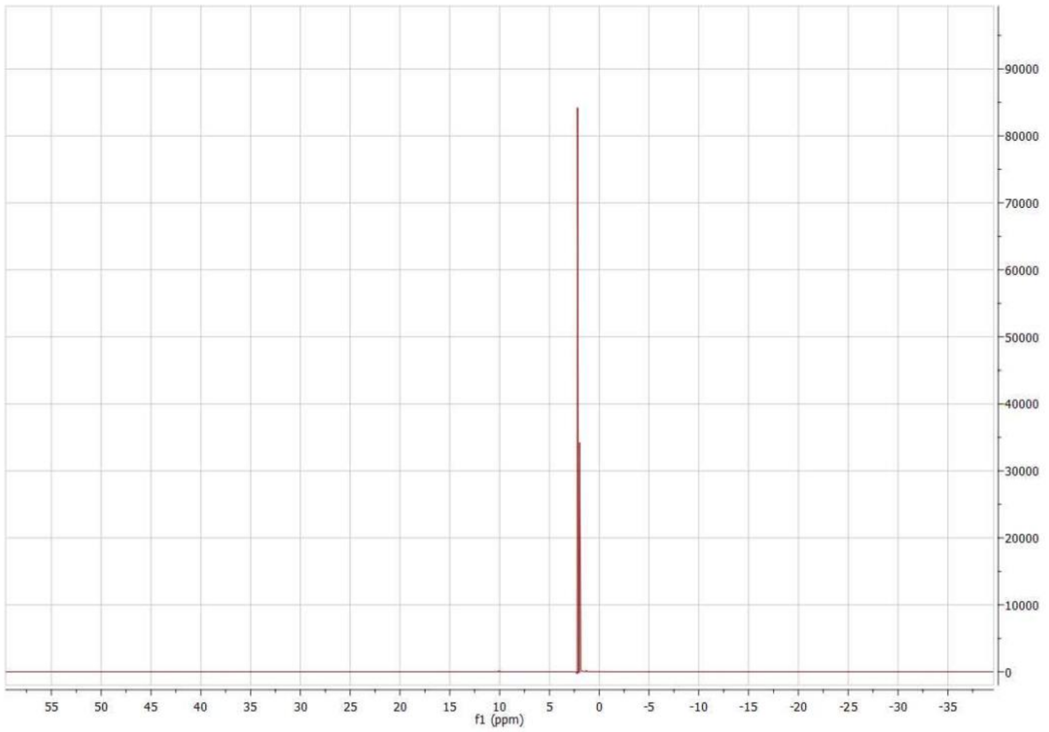

Figure S18. ^{13}C-NMR spectrum in CD_3CN.

B) 3-pyridinecarboxaldehyde:

***3-pyridinecarboxaldehyde in $CDCl_3$: aldehyde (100%)**

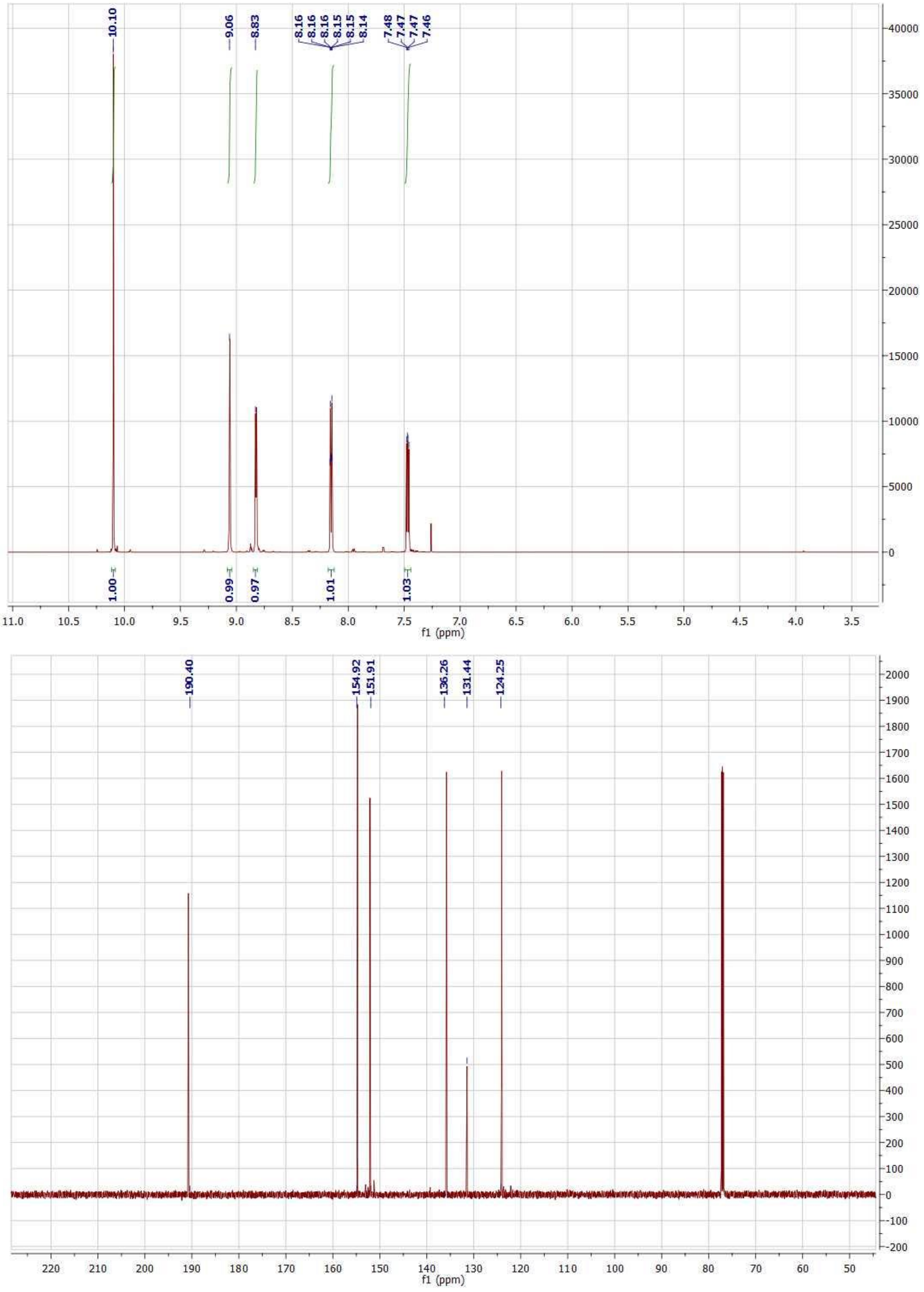

Figure S19. NMR spectra for the 3-pyridinecarboxaldehyde in $CDCl_3$. Aldehyde form (100%): ^{1}H NMR (600 MHz, $CDCl_3$) δ (ppm) 10.10 (s, 1H, -C**H**O), 9.06 (d, J = 1.5 Hz, H_2), 8.82 (dd, J = 4.8, 1.7 Hz, H_6), 8.15 (d, J = 7.9 Hz, H_4), 7.47 (dd, J = 7.8, 4.8 Hz, H_5). ^{13}C NMR (151 MHz, $CDCl_3$) δ (ppm) 190.74 (-**C**HO), 154.69 (C_6), 152.17 (C_2), 135.80 (C_4), 131.49 (C_3), 124.07 (C_5).

***3-pyridinecarboxaldehyde in CD_3OD: aldehyde (25%), *gem*-diol (75%)**

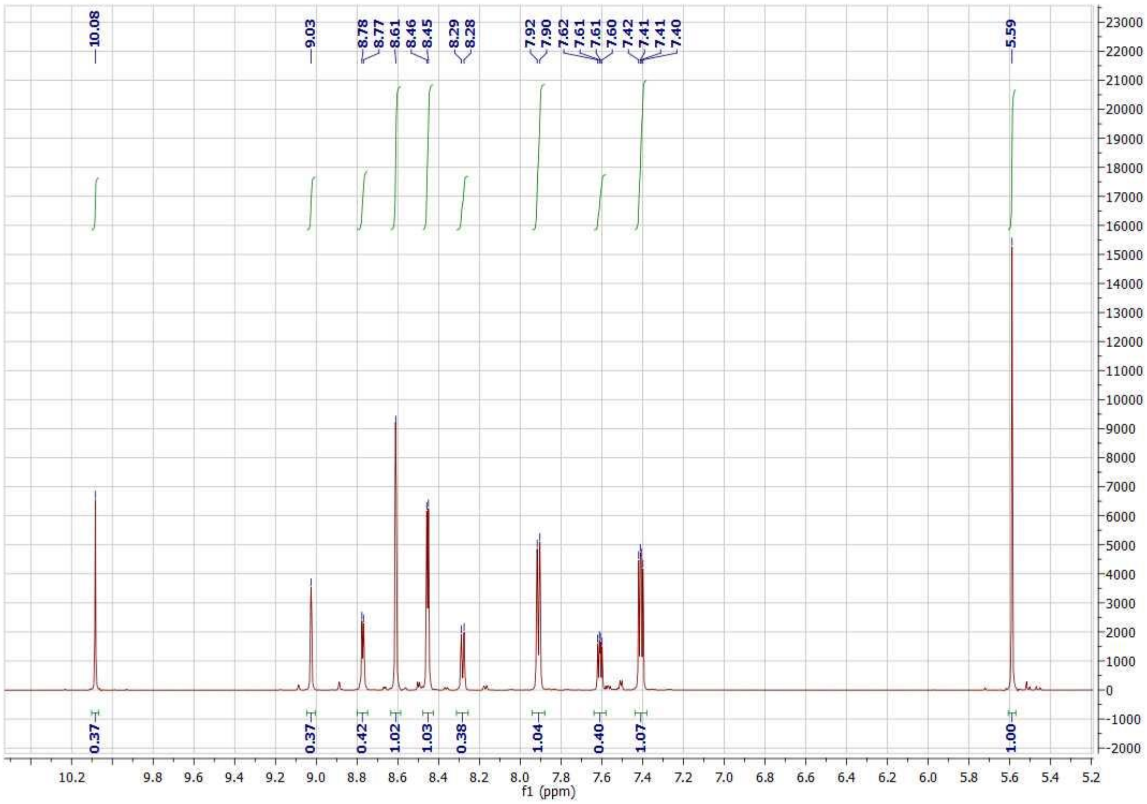

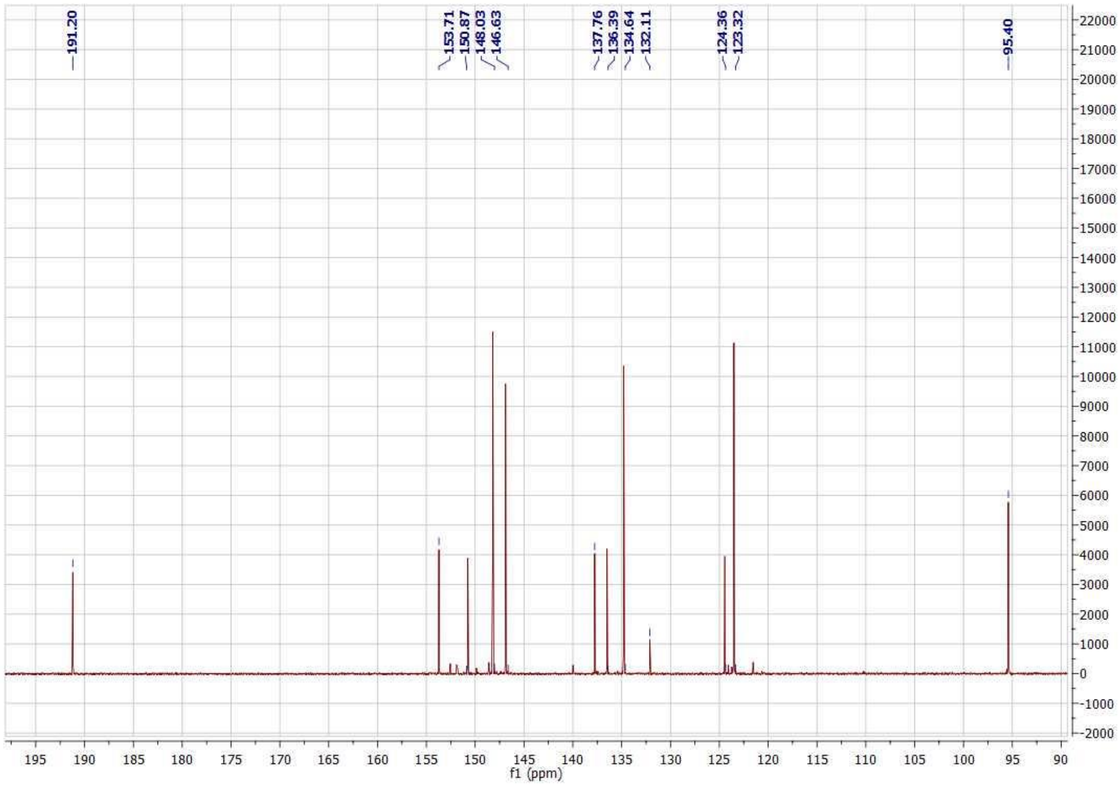

Figure S20. NMR spectra for the 3-pyridinecarboxaldehyde in CD_3OD. ^1H NMR (600 MHz, CD_3OD) **Aldehyde form (25.0%):** δ (ppm) 10.11 (s, 1H, (-C**H**O)), 9.05 (s, 1H, H_2), 8.79 (d, J = 3.6 Hz, 1H, H_6), 8.30 (d, J = 7.9 Hz, 1H, H_4), 7.63 (dd, J = 7.9 Hz, J = 5.0 Hz, 1H, H_5). ***gem*-diol**

form (75.0%): 8.63 (s, 1H, H_2), 8.48 (d, *J* = 4.9 Hz, 1H, H_6), 7.93 (d, *J* = 7.9 Hz, 1H, H_4), 7.43 (dd, *J* = 7.9 Hz, *J* = 4.9 Hz, 1H, H_5), 5.61 (s, 1H, (-C**H**(OH)$_2$). ^{13}C NMR (151 MHz, CD_3OD) **Aldehyde form (25.0%):** δ (ppm) 191.20 (-**C**HO), 153.71 (C_6), 150.75 (C_2), 137.76 (C_3), 132.06 (C_4), 124.44 (C_5) ***gem*-diol form (75.0%):** 148.20 (C_6), 146.89 (C_2), 136.50 (C_3), 134.79 (C_4) 123.50 (C_5), 95.32 (-**C**H(OH)$_2$).

***Solution-state NMR experiments with 3-pyridindecarboxaldehyde and $CoCl_2/CD_3OD$:**

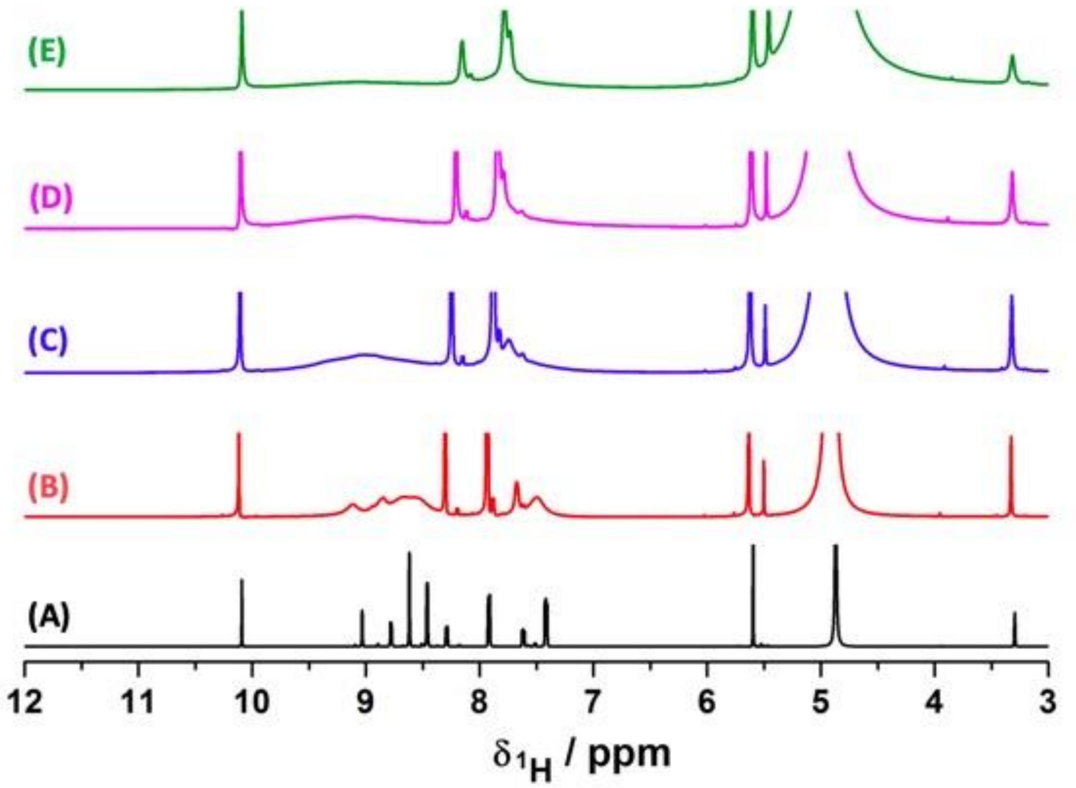

Figure S21. ^{1}H solution-state NMR for 3-pyridinecarboxaldehyde (0.28 mmol) in CD_3OD and different $CoCl_2$ additions: 0 (A), 1.83 (B), 5.49 (C), 7.32 (D) and 9.0 μmoles (E).

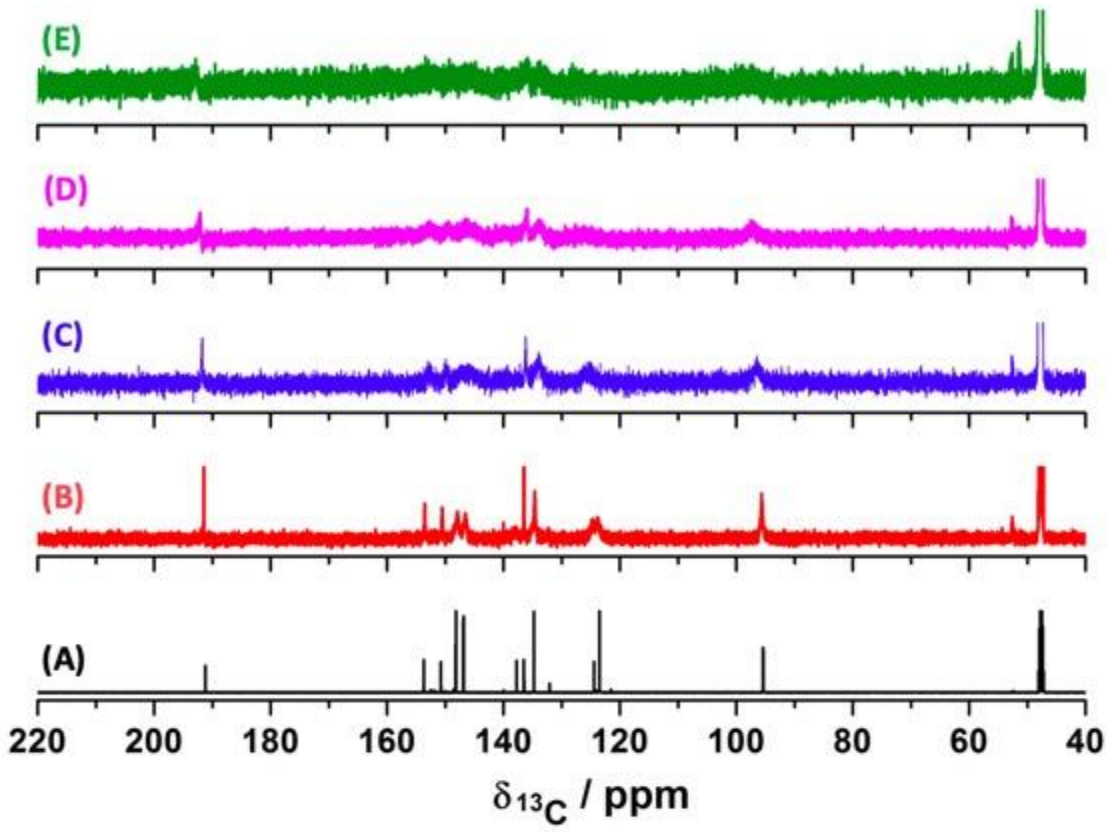

Figure S22. ^{13}C solution-state NMR spectra for 3-pyridinecarboxaldehyde (0.28 mmol) in CD_3OD and different $CoCl_2$ additions: 0 (A), 1.83 (B), 5.49 (C), 7.32 (D) and 9.0 μmoles (E).

***Solution-state NMR experiments with 3-pyridindecarboxaldehyde and $CuCl_2$/CD_3OD:**

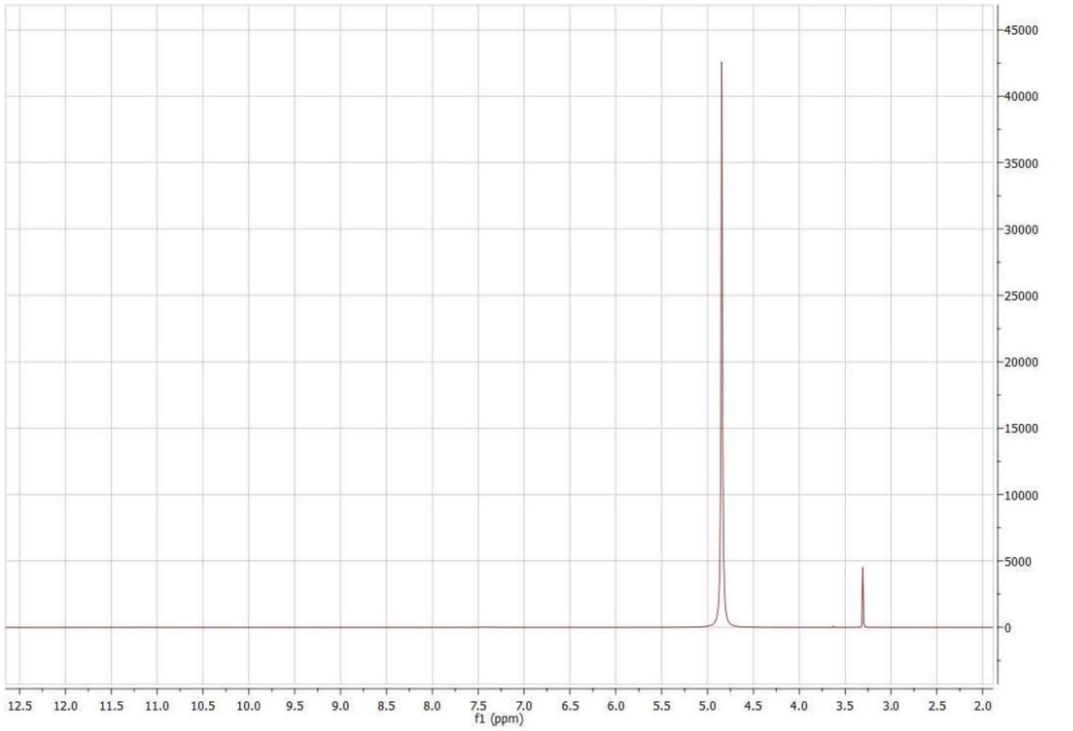

Figure S23. ^{1}H-NMR spectrum in CD_3OD.

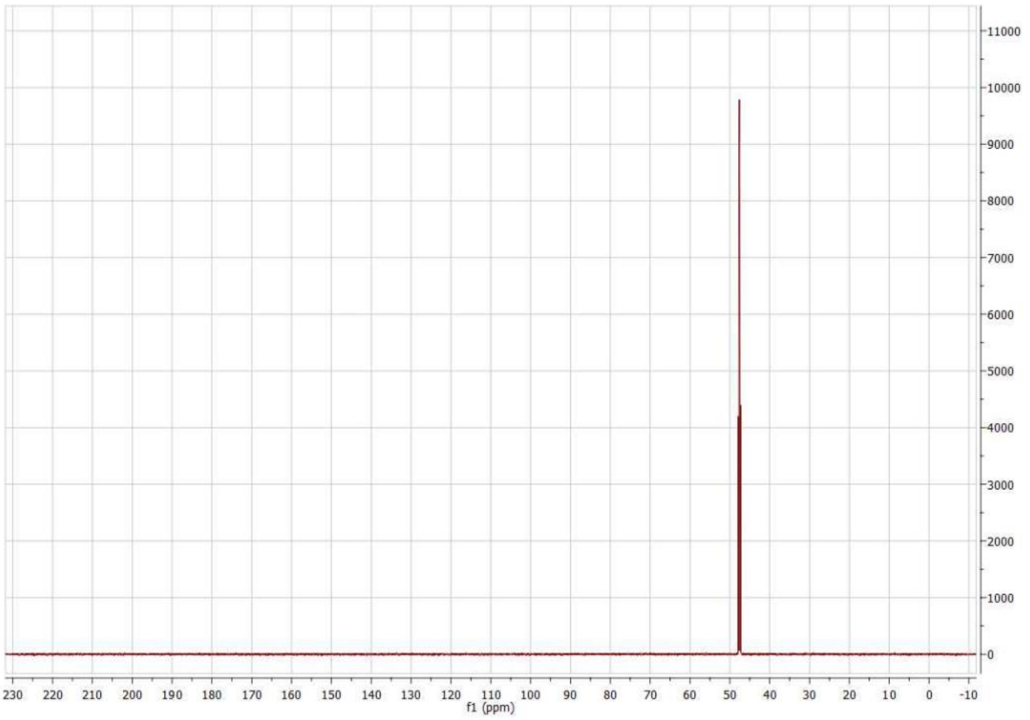

Figure S24. ^{13}C-NMR spectrum in CD_3OD.

Coordinates x,y,z for the DFT calculations:

***Cu(II)-4-piridinecarboxaldehyde**

Cu, 0,-0.0132942578,-0.10134342,0.0455736234
C, 0,2.6868933239,0.5743059052,1.1280228658
C, 0,2.7857349022,-0.5623989964,-0.8795918029
C, 0,4.0689518609,0.6366230684,1.2072072674
H, 0,2.0397191946,0.967618745,1.9063790086
C, 0,4.1746611669,-0.5514871418,-0.865976889
H, 0,2.215888519,-0.9824284486,-1.7034532151
H, 0,4.547559302,1.1294752392,2.0457441735
H, 0,4.7325561626,-0.9960117766,-1.6812409605
C, 0,-0.3745888812,-2.8931693693,-0.9678324406
C, 0,0.6034547521,-2.8168792008,1.1212456941
C, 0,-0.3324362168,-4.2799372119,-0.9828453073
H, 0,-0.7430584452,-2.3117174865,-1.8085137803
C, 0,0.6901332573,-4.2005933114,1.1763266733
H, 0,0.9231993019,-2.1794683738,1.9409056804
H, 0,-0.6999387947,-4.8286003281,-1.8425545859
H, 0,1.132061488,-4.6865959173,2.0382512904
C, 0,-2.7615778967,0.298632749,-1.0665194781
C, 0,-2.7750623137,-0.7403251248,0.9954951288
C, 0,-4.1450343258,0.2552353234,-1.1416918704
H, 0,-2.1441883675,0.6945629868,-1.8679045847
C, 0,-4.1611687601,-0.8313785907,0.9884280896
H, 0,-2.1732837142,-1.0788014348,1.8343770691
H, 0,-4.6585541156,0.6566626119,-2.0085645283
H, 0,-4.6857384486,-1.2883721618,1.8185488359
C, 0,0.4731641173,2.6588048101,-0.9714978583
C, 0,-0.7398090387,2.6330486184,0.9929528828
C, 0,0.428750967,4.0451982409,-1.0295934236
H, 0,0.9464632523,2.0599891202,-1.7437877209
C, 0,-0.840543425,4.0157744289,0.9975666189
H, 0,-1.141505534,2.0167388102,1.7918353432
H, 0,0.9230160001,4.5732395916,-1.8371520926
H, 0,-1.3600202828,4.5255937148,1.8005138308
C, 0,4.8325540695,0.0588847291,0.1962994803
C, 0,-4.8628748123,-0.3251274769,-0.0991925178
C, 0,-0.2461957631,4.74080982,-0.0307985211
C, 0,0.211273165,-4.9531043123,0.108102656
N, 0,-0.1012669859,1.9683256067,0.0203226164
N, 0,-2.0906598426,-0.19050907,-0.0144172254
N, 0,0.0796043471,-2.1762604329,0.068477874
N, 0,2.0585776016,-0.0170579786,0.1018973036
C, 0,0.2313995876,-6.4605014385,0.1608192862
H, 0,-0.7116104466,-6.8181891827,0.6188545622
O, 0,1.3118808261,-6.8703477458,0.9517537892
H, 0,1.1046161056,-7.7332769036,1.3312028118
O, 0,0.2945836873,-6.9341649337,-1.1506785027
C, 0,0.1769887996,-8.3304928624,-1.245849073
H, 0,0.1196249564,-8.5750181217,-2.3082279621

H, 0,1.0477770516,-8.8424881804,-0.814314275
H, 0,-0.7371709069,-8.6926419382,-0.7468502118
C, 0,6.3458304654,0.049246069,0.3258368034
O, 0,6.8604447128,-0.3373787976,-0.9068815011
O, 0,6.7910112774,1.2945067388,0.7340899679
H, 0,7.4963182558,1.134128415,1.3788578197
O, 0,6.7524367324,-0.8374428131,1.341201898
H, 0,6.4848556898,-1.7281455983,1.0718720664
C, 0,8.2657694812,-0.235883017,-1.0209504014
H, 0,8.5234143221,-0.6617760744,-1.9920032916
H, 0,8.586555437,0.8109712819,-0.986095375
H, 0,8.7739883821,-0.8021990032,-0.2306300527
C, 0,-0.4206326122,6.2481211131,-0.089767798
O, 0,0.613598696,6.7440194894,-0.8764569329
O, 0,-0.4315480463,6.7807292972,1.1871888788
H, 0,-1.1320670974,7.4497861307,1.1977650411
O,0,-1.6751663468,6.579356458,-0.6377845046
H, 0,-1.6935001523,6.2398209472,-1.5443528892
C, 0,0.70130522,8.1545048174,-0.9194274428
H, 0,1.4898221345,8.3880020594,-1.6366369136
H, 0,-0.2405962777,8.6047021881,-1.2563529394
H, 0,0.9704998221,8.559247868,0.0622331183
C, 0,-6.3805670656,-0.3588688195,-0.1723159011
O, 0,-6.8095266732,-1.3355278291,0.7227951785
O, 0,-6.8682518125,0.9040289101,0.122210074
H, 0,-7.6233421185,1.0499269401,-0.4677345195
O, 0,-6.8376653268,-0.6403275464,-1.471247253
H, 0,-6.5759930963,-1.5497960528,-1.6767031952
C, 0,-8.2014532541,-1.3411052037,0.971766595
H, 0,-8.7747058914,-1.4459852566,0.0419826796
H, 0,-8.3920477367,-2.2053970796,1.6100878253
H, 0,-8.5108625272,-0.4282264637,1.491900968
Cl, 0,-0.0908317569,-0.0658916217,2.723958145
Cl, 0,0.0621749732,-0.1181227944,-2.643683777

***Complex Cu(II)-3-pyridinecarboxaldehyde**

C, 0,-2.6855194309,0.0008259339,-0.1629425028
C, 0,-4.0566678722,0.0011953424,-0.402833769
C, 0,-3.5815402985,0.0010230854,-2.7486033729
C, 0,-2.2258103968,0.0006602437,-2.4292599142
H, 0,-2.2865009892,0.0007518851,0.8579398064
H, 0,-3.8835414368,0.0010882364,-3.7905289159
H, 0,-1.445710289,0.0004388969,-3.1872214027
Cu, 0,0.2251934006,0.0000768917,-0.7441511263
C, 0,3.1747194307,-0.0005813888,-0.499122174
C, 0,2.1294886401,-0.0002177216,1.5633244345
C, 0,4.4334706065,-0.0007972134,0.0936915725
H, 0,3.0398480276,-0.0006236894,-1.5804486434
C, 0,3.3540062549,-0.0004236461,2.2294189593
H, 0,1.1820744642,0.0000344197,2.1104575859
H, 0,3.3641660197,-0.0003458419,3.3141594965

C, 0,-0.2800271997,-2.534469075,0.6981029296
C, 0,0.4693854253,-2.8774739859,-1.4627107628
C, 0,-0.3608185621,-3.9048507517,0.9276870269
H, 0,-0.5521740426,-1.8108535432,1.475084792
C, 0,0.4140606084,-4.2606844926,-1.3086934212
H, 0,0.7917248296,-2.4039798672,-2.387332761
H, 0,0.7001966537,-4.900782205,-2.1364477134
C, 0,-0.2787622468,2.5350449485,0.6977156338
C, 0,0.4709152764,2.8774343009,-1.4631129723
C, 0,-0.3588404765,3.9054920017,0.9271526669
H, 0,-0.5513099085,1.8116536468,1.4747623795
C, 0,0.4163114656,4.2606889665,-1.3092422965
H, 0,0.7930279529,2.4036121347,-2.3876483798
H, 0,0.7028122484,4.9005573497,-2.137047607
N, 0,0.1288521145,-2.0352675806,-0.4769002374
N, 0,2.0473012552,-0.0003026704,0.2232868007
N, 0,-1.7909540347,0.0005606708,-1.1612501485
N, 0,0.1299023244,2.0355150646,-0.4772243557
Cl, 0,1.0557880575,-0.000197962,-3.0445629193
Cl, 0,-1.0536353424,0.0005992018,2.7980813727
C, 0,-0.0038634871,4.7874589375,-0.0966529214
H, 0,-0.0652123699,5.8576800971,0.0807580701
C, 0,-0.8169970335,4.3914819183,2.2509202561
H, 0,-1.0737449385,3.6006725216,2.9827329046
C, 0,-4.5151887433,0.0012948189,-1.7226144628
H, 0,-5.5851449551,0.0015829183,-1.9118248087
C, 0,-4.9923527663,0.0014810216,0.7473365011
H, 0,-4.514543553,0.0012809672,1.7466560362
C, 0,-0.0063459978,-4.7871142081,-0.0960361872
H, 0,-0.0682508039,-5.8572844934,0.0814869714
C, 0,-0.8191831615,-4.3904515338,2.251525459
H, 0,-1.0755705695,-3.5994262662,2.9832307847
C, 0,5.6410002943,-0.0011015751,-0.7611627868
H, 0,5.4478235686,-0.0011209007,-1.8531750999
C, 0,4.5255065922,-0.0007195388,1.4891272694
H, 0,5.5070077455,-0.0008902435,1.9550823391
O, 0,6.7711650902,-0.001299439,-0.3236264065
O, 0,-0.9110729918,-5.5676902443,2.5272312008
O, 0,-6.1979355287,0.0016915763,0.6176427495
O, 0,-0.9081889203,5.56879908,2.5265210701

Magnetic shielding constant $\sigma_{(orb)}$ for the standard TMS (in ppm)

Nuclei	Value (PBE0-D_3, 6-31G**(d,p)
^{13}C	185

Table S13. g/A tensors obtained by computational calculations. The experimental g/A-values are included for comparison.

	Experimental	Computational Calculations	
	**g/A**	**g/A**	**g/A eigenvectors**
**Cu(II)-4-pyridinecarboxaldehyde**	2.246 / n.d.	2.255 / -565.7	[0.0067683, 0.0246772, 0.9996726]<br>[0.0238158, -0.0059625, 0.9996986]
	2.067 / n.d.	2.082 / 51.1	[-0.9952482, -0.0972739, 0.0043371]<br>[-0.9963194, -0.0822241, 0.0242257]
	2.030 / n.d.	2.079 / 57.4	[0.0971350, -0.9949517, -0.0252183]<br>[0.0823437, -0.9965960, -0.0039823]
**Cu(II)-3-pyridinecarboxaldehyde**	2.215 / 117	2.237 / -578.3	[0.0000099, -0.3457802, 0.9383156]<br>[0.3454985, -0.0000016, 0.9384193]
	2.084 / n.d.	2.073 / 15.3	[-1.0000000, 0.0001710, 0.0000735]<br>[-0.9384193, -0.0002015, 0.3454985]
	2.055 / 161	2.068 / 33.1	[-0.0001858, -0.9383155, -0.3457801]<br>[0.0001885, -1.0000000, -0.0000711]

n.d., non-detectable.
A-values in MHz

Atom coordinates of the copper(II) centers of Cu(II)-4- pyridinecarboxaldehyde complex:
Cu - 0.01254000 -0.09639600 0.06301300
N - 0.19266900 1.96738300 0.04643400
N - 2.08408100 -0.27776400 0.00991000
N - 0.17273600 -2.16519000 0.07720700
N - 2.05371700 0.07986600 0.11244900
Cl -0.08176200 -0.07483100 2.74177300
Cl - 0.05374500 -0.09935700 -2.62653700

Atom coordinates of the copper(II) centers of Cu(II)-3-pyridinecarboxaldehyde complex:
Cu - 0.22514800 -0.00005500 -0.73600600
N - 0.12783400 -2.03542900 -0.46932900
N - 2.04311900 -0.00036400 0.23918200
N - 1.78920500 0.00029900 -1.16168800
N - 0.12855600 2.03535400 -0.46932700
Cl - 1.06553100 -0.00008000 -3.03286000
Cl - 1.06875500 0.00008300 2.80074800

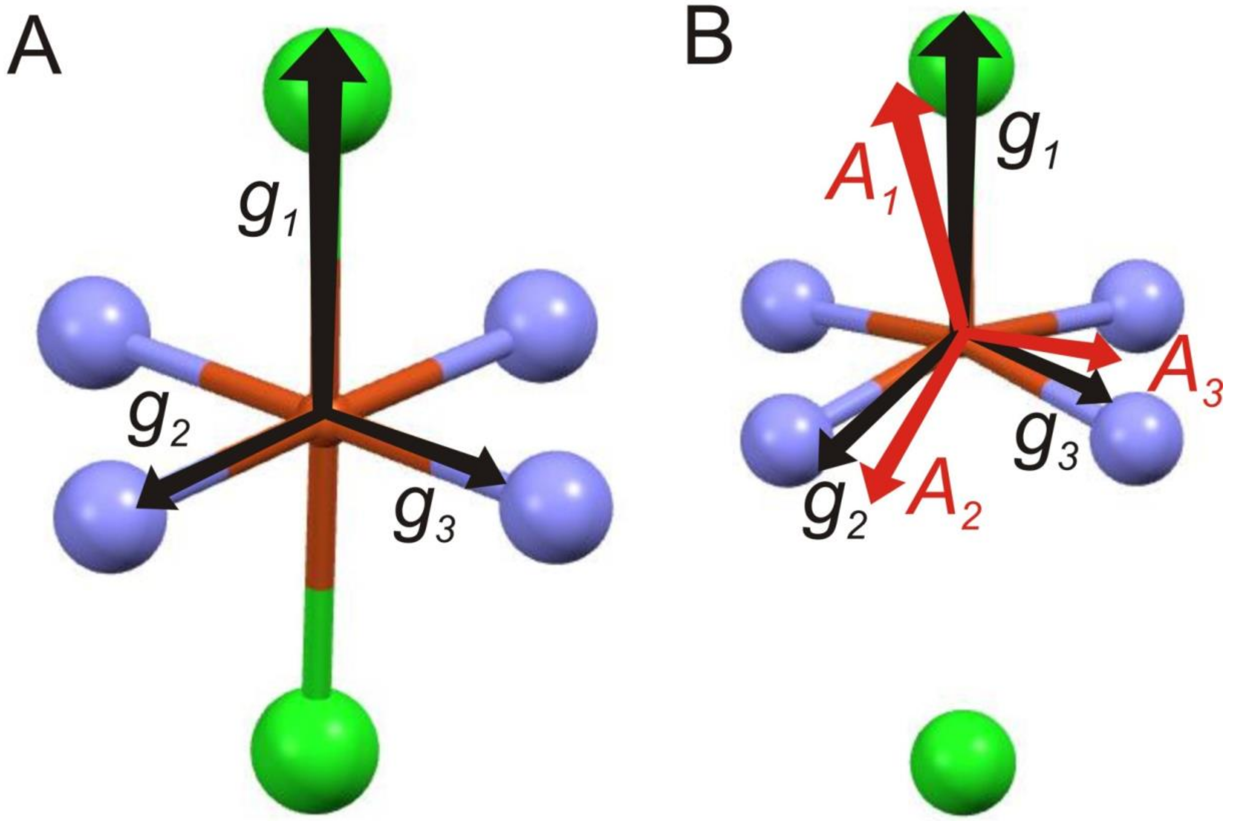

Figure S25. g/A-tensor orientations of the copper(II) centers of Cu(II)-4- pyridinecarboxaldehyde (A) and Cu(II)-3-pyridinecarboxaldehyde (B) in the molecular frame A-eigenvectors in panel A are omitted for clarity, but as shown in table S13, they are nearly coincidental to those of g-eigenvectors.

Table S14. EPR parameters and its contribution to pNMR shielding tensor for the Cu(II)-4-pyridinecarboxaldehyde (Complex 1) and Cu(II)-3-pyridinecarboxaldehyde (Complex 2) (*g* is dimensionless, and A is in MHz).

Nucleus	Property	Complex 1 (Orthoester)	Complex 1 (Hemiacetal)	Complex 2
	g_{iso}	2.1387221		2.1258158
C_2	σ_{iso}(orb)	27.6	28.2	22.5
	A_{iso}	-0.8413	-0.7058	-0.1142
	$g_e.A_{FC}$	-1.685	-1.413	-0.2286
	$\Delta g_{iso}.A_{FC}$	-0.1148	-0.09627	-0.01409
	$\Delta g_{aniso}.A_{dip}$	0.0	0.0	0.0
C_3	σ_{iso}(orb)	64.3	64.3	50.9
	A_{iso}	3.488	3.474	3.573
	$g_e.A_{FC}$	6.983	6.957	7.155
	$\Delta g_{iso}.A_{FC}$	0.4757	0.4740	0.4413
	$\Delta g_{aniso}.A_{dip}$	0	0	0
C_4	σ_{iso}(orb)	31.5	33.0	46.0
	A_{iso}	-0.8651	-0.8644	-0.9075
	$g_e.A_{FC}$	-1.732	-1.731	-1.817
	$\Delta g_{iso}.A_{FC}$	-0.1180	-0.1179	-0.1121
	$\Delta g_{aniso}.A_{dip}$	0	0	0
C_5	σ_{iso}(orb)	64.8	64.5	59.8
	A_{iso}	3.449	3.461	3.704
	$g_e.A_{FC}$	6.907	6.930	7.416
	$\Delta g_{iso}.A_{FC}$	0.4705	0.4721	0.4574
	$\Delta g_{aniso}.A_{dip}$	0	0	0
C_6	σ_{iso}(orb)	27.6	27.8	25.6

	**A_{iso}**	-0.8506	-0.8094	-1.258
	**$g_e.A_{FC}$**	-1.703	-1.621	-2.519
	**$\Delta g_{iso}.A_{FC}$**	-0.1160	-0.1104	-0.1553
	**$\Delta g_{aniso}.A_{dip}$**	0	0	0
-**C**H(OH)(OCH_3) ($C_{7'}$)	**σ_{iso}(orb)**	//////////////////////////	90.6	//////////////////
	**A_{iso}**	//////////////////////////	0.2248	//////////////////
	**$g_e.A_{FC}$**	//////////////////////////	0.4501	//////////////////
	**$\Delta g_{iso}.A_{FC}$**	//////////////////////////	0.03066	//////////////////
	**$\Delta g_{aniso}.A_{dip}$**	//////////////////////////	0	//////////////////
-**C**$(OH)_2$(OCH_3) (C_7)	**σ_{iso}(orb)**	78.5	//////////////////////////	//////////////////
	**A_{iso}**	0.2425	//////////////////////////	//////////////////
	**$g_e.A_{FC}$**	0.4855	//////////////////////////	//////////////////
	**$\Delta g_{iso}.A_{FC}$**	0.03307	//////////////////////////	//////////////////
	**$\Delta g_{aniso}.A_{dip}$**	0	//////////////////////////	//////////////////
-O**C**H_3 ($C_{8/8'}$)	**σ_{iso}(orb)**	142.4	134.9	//////////////////
	**A_{iso}**	0.0038	0.0033	//////////////////
	**$g_e.A_{FC}$**	0.007609	0.006608	//////////////////
	**$\Delta g_{iso}.A_{FC}$**	0.0005183	0.0004501	//////////////////
	**$\Delta g_{aniso}.A_{dip}$**	0	0	//////////////////
-**C**HO (C_7)	**σ_{iso}(orb)**	//////////////////////////	//////////////////////////	7.1
	**A_{iso}**	//////////////////////////	//////////////////////////	0.300
	**$g_e.A_{FC}$**	//////////////////////////	//////////////////////////	0.600
	**$\Delta g_{iso}.A_{FC}$**	//////////////////////////	//////////////////////////	0.03704
	**$\Delta g_{aniso}.A_{dip}$**	//////////////////////////	//////////////////////////	0

Table S15. Observed and calculated distances with a variety of functionals and basis set for 4-piridinecarboxaldehyde-Cu(II).

X-ray N-Cu	B3LYP/ 6-31**G(d,p)/ LANL2DZ N-Cu	B3LYP/ cc-pVTZ/ LANL2DZ N-Cu	PBE0-D3/ 6-31**G(d,p)/ extrabasis for Cu(II) N-Cu	B3PW91/ 6-31**G(d,p)/ LANL2DZ N-Cu	Two step optimization([a]) N-Cu
2.0	2.1	2.1	2.1	2.1	2.1
2.0	2.6	2.5	2.9	2.5	2.1
2.0	2.1	2.1	2.3	2.1	2.1
2.0	2.5	2.6	2.0	2.5	2.1

Table S16. Observed and calculated distances with a variety of functionals and basis set for 3-piridynecarboxaldehyde-Cu(II).

X-ray N-Co	B3LYP/ 6-31**G(d,p)/ LANL2DZ N-Cu	B3LYP/ cc-pVTZ/ LANL2DZ N-Cu	PBE0-D3/ 6-31**G(d,p)/ extrabasis for Cu(II) N-Cu	B3PW91/ 6-31**G(d,p)/ LANL2DZ N-Cu	Two step optimization([a]) N-Cu
2.2	2.1	2.1	2.1	2.1	2.1
2.1	2.6	2.4	3.0	2.6	2.1
2.1	2.5	2.1	2.5	2.2	2.1
2.2	2.1	2.6	2.0	2.5	2.1

[a]*See Experimental Section 4.5 in the manuscript.*

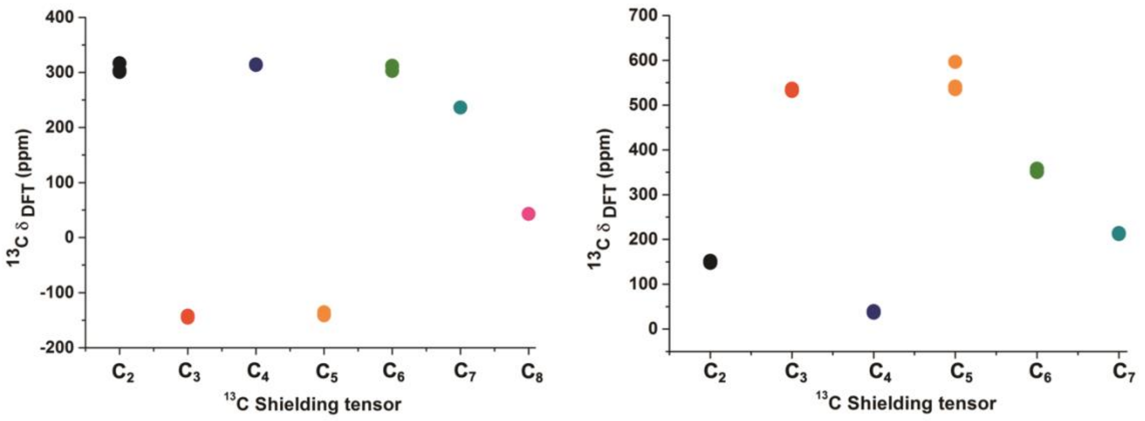

Figure S26. Dispersion of values obtained from DFT calculations. Values obtained for the orthoester moiety in the copper complex for the 4-pyridinecarboxaldehyde (left) and for Cu(II)-3-pyridinecarboxaldehyde (right). The numbering corresponds to those in Figures 1 and 2 and the color represents each of the nuclei.

Solid-state NMR results.

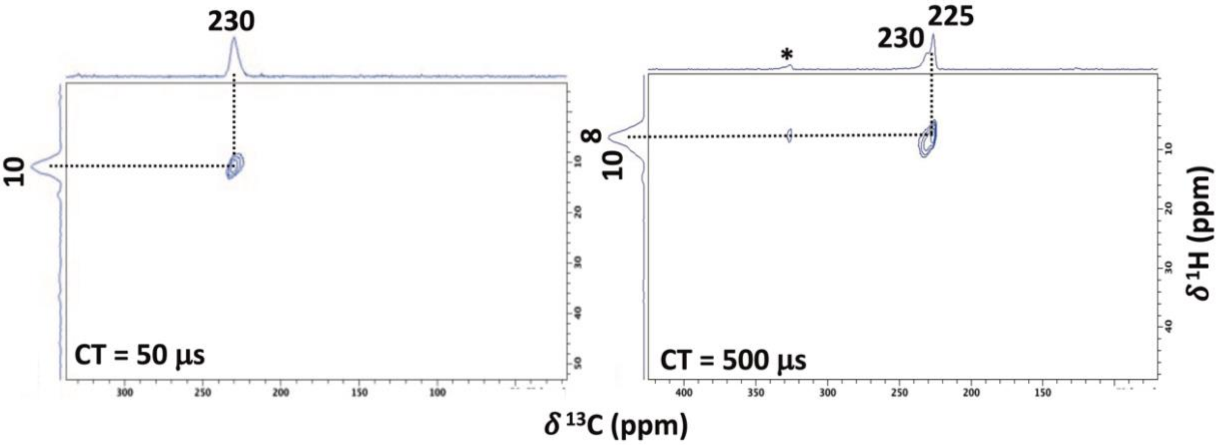

Figure S27. 2D ^{1}H-^{13}C HETCOR spectra for the single crystals obtained with 4-pyridinecarboxaldehyde and $CuCl_2$ in methanol with a contact time of 50 (left) or 500 µs (right) (MAS rate: 15 kHz).

References:

(1) Sheldrick, G. M. Phase Annealing in SHELX-90: Direct Methods for Larger Structures. *Acta Crystallogr. Sect. A* **1990**, *46*, 467–473. https://doi.org/10.1107/S0108767390000277.

(2) Sheldrick, G. M. A Short History of SHELX. *Acta Crystallogr. Sect. A Found. Crystallogr.* **2007**, *64*, 112–122. https://doi.org/10.1107/S0108767307043930.

(3) Crespi, A. F.; Vega, D.; Chattah, A. K.; Monti, G. A.; Buldain, G. Y.; Lázaro-Martínez, J. M. Gem-Diol and Hemiacetal Forms in Formylpyridine and Vitamin-B6-Related Compounds: Solid-State NMR and Single-Crystal X-Ray Diffraction Studies. *J. Phys. Chem. A* **2016**, *120*, 7778–7785. https://doi.org/10.1021/acs.jpca.6b07898.
